# The contribution of common and rare genetic variants to variation in metabolic traits in 288,137 East Asians

**DOI:** 10.1038/s41467-022-34163-2

**Published:** 2022-11-04

**Authors:** Young Jin Kim, Sanghoon Moon, Mi Yeong Hwang, Sohee Han, Hye-Mi Jang, Jinhwa Kong, Dong Mun Shin, Kyungheon Yoon, Sung Min Kim, Jong-Eun Lee, Anubha Mahajan, Hyun-Young Park, Mark I. McCarthy, Yoon Shin Cho, Bong-Jo Kim

**Affiliations:** 1 Division of Genome Science, Department of Precision Medicine, National Institute of Health, Cheongju-si, Republic of Korea; 2 DNALink, Seoul, Republic of Korea; 3 Genentech, 1 DNA Way, South San Francisco, CA USA; 4 Wellcome Centre for Human Genetics, University of Oxford, Oxford, UK; 5 Department of Precision Medicine, National Institute of Health, Cheongju-si, Republic of Korea; 6 Biomedical Science, Hallym University, Chuncheon, Republic of Korea

**Keywords:** Genome-wide association studies, Rare variants, Biomarkers, Endocrine system and metabolic diseases

## Abstract

Metabolic traits are heritable phenotypes widely-used in assessing the risk of various diseases. We conduct a genome-wide association analysis (GWAS) of nine metabolic traits (including glycemic, lipid, liver enzyme levels) in 125,872 Korean subjects genotyped with the Korea Biobank Array. Following meta-analysis with GWAS from Biobank Japan identify 144 novel signals (MAF ≥ 1%), of which 57.0% are replicated in UK Biobank. Additionally, we discover 66 rare (MAF < 1%) variants, 94.4% of them co-incident to common loci, adding to allelic series. Although rare variants have limited contribution to overall trait variance, these lead, in carriers, substantial loss of predictive accuracy from polygenic predictions of disease risk from common variant alone. We capture groups with up to 16-fold variation in type 2 diabetes (T2D) prevalence by integration of genetic risk scores of fasting plasma glucose and T2D and the I349F rare protective variant. This study highlights the need to consider the joint contribution of both common and rare variants on inherited risk of metabolic traits and related diseases.

## Abstract (web summary)

Metabolic traits are heritable intermediate phenotypes widely used in assessing the risk of various diseases. By conducting a genome-wide meta-analysis for metabolic traits in 288,137 East Asians, the authors highlight the interplay of common and rare variants on inherited risk of metabolic traits.

## Introduction

Metabolic traits available from routine biochemical tests represent intermediate phenotypes widely-used in assessing disease risk. Glycemic traits such as levels of fasting plasma glucose (FPG), 2-h glucose after a 75-g oral glucose tolerance test, and hemoglobin A1c (HbA1c) are used as diagnostic tests for type 2 diabetes (T2D)^1^; dyslipidemia, an abnormal level of lipid (high lipoprotein cholesterol (HDL), low density lipoprotein cholesterol (LDL), triglyceride (TG), and total cholesterol (TC)) in the blood, represents a major risk factor for coronary artery disease and stroke^2^; and increased levels of liver enzymes (alanine aminotransferase (ALT), aspartate aminotransferase (AST), and γ-glutamyl transferase (GGT)) reflect liver injury and disease^3,4^. Given the heritable nature of these metabolic traits^5–7^, there is potential to use individual genetic information as an additional tool to stratify disease risk and provide clinical decision support^8^, as well as to provide inference about disease biology.

Previous large-scale genetic association data have overwhelmingly been derived from studies of European ancestry individuals^9^. This Eurocentric bias in variant discovery has been shown to lead to an inaccurate inference of genetic risk in individuals of non-European ancestry^10^. Recently, large-scale biobanks, such as UK Biobank (UKB)^11,12^, Million Veteran Program^13^, BioBank Japan (BBJ)^14^, as well as a number of international consortia^15–17^ have begun to demonstrate the value of generating large-scale trans-ethnic genetic association data for medically-relevant metabolic traits. This warrants efforts to generate GWAS data across populations that can, collectively, provide a more diverse ancestral background, and take account of differences in genetic architecture and allele frequencies between populations^10^.

Recent studies have demonstrated the clinical potential offered by aggregating individual measures of genetic risk in the form of polygenic risk scores (PRS) across a growing range of diseases: these PRS can define substantial tranches of the population who differ markedly with respect to disease prevalence and incidence^8,18,19^. Most of these PRS focus on common variants (typically, MAF > 1%). Although sequencing and customized microarray-based studies are now identifying a growing number of rare functional variants (typically in coding regions)^8,16,17,20–30^, the contribution of rare variants to population trait variance and the value of their inclusion within PRS remain poorly characterized. Recent studies have reported that background polygenic risk contributes to the variable penetrance of rare pathogenic mutations in genes such as *LDLR*, *APOB*, and *PCSK9* for coronary artery disease^31^, *BRCA1* and *BRCA2* for breast cancer^29^, and *MYOC* for glaucoma^32^.

The Korea National Institute of Health launched the Korea Biobank Array (KBA) project^33^ in 2014 to characterize genetic variation influencing complex traits such as T2D and obesity in the Korean population. The project involved analyzing cohorts of the population-based Korean Genome and Epidemiology Study (KoGES)^34^ using a customized SNP microarray of ~830 K variants. This array was designed to offer optimal tagging of common variants in East Asian populations, together with large-scale evaluation of 208 K functional variants (70% of them with MAF < 1%) retrieved from 2576 sequenced Korean subjects^33^.

Here, we focus on analysis of nine metabolic traits with clear medical relevance, including two glycemic traits (FPG and HbA1c), four lipid traits (HDL, LDL, TG, and TC) and three liver enzymes (ALT, AST, and GGT). We use the KBA to assess association of these traits with both common and rare functional variants in 125,872 Korean subjects aged 40–69 years, and extend these insights by analyses in both the Biobank of Japan and UK Biobank. As a result, we identified over 1000 common and rare variants associated with nine metabolic traits. These large-scale analyses are further utilized to explore the contribution of common and rare variants to variation of metabolic trait measures from both mechanistic and clinical perspectives. We demonstrate that the rare variants, in carriers, lead substantial loss of predictive accuracy from common variants based polygenic predictions of metabolic traits and T2D.

## Results

### Discovery of metabolic traits associated common variants in 126 K Korean individuals

**Fig. 1:**
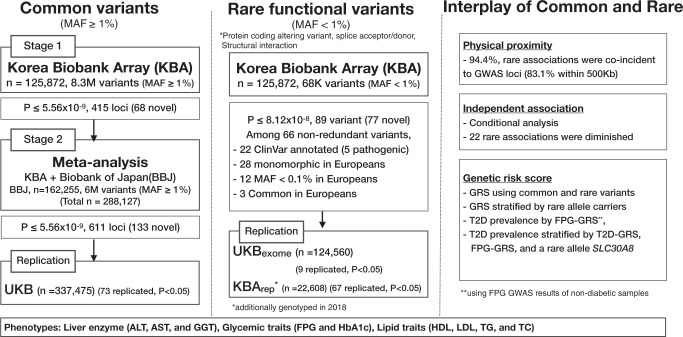
Overall analysis scheme. Flow chart of the overall analysis including summarized results of common variants, rare functional variants, and interplay of common and rare variants.

The study scheme is summarized in Fig. 1. A total of 134,721 KoGES samples were genotyped with the KBA and after quality control, 125,872 of these were taken forward for imputation (Methods section). A merged reference panel, combining whole genome sequencing data from 2504 1000Genomes Phase 3 participants and 397 samples from the Korean Reference Genome^33,35^ was used for imputation. Imputed data was filtered to retain 8.3 M high quality common variants (info ≥ 0.8 and MAF ≥ 1%). Demographic characteristics of this “126 K” sample set are provided in Supplementary Data 1.

Single variant association analysis (linear regression) of the nine metabolic traits was performed using EPACTS v3.4.6, assuming an additive mode of inheritance. A variant was considered associated if the variant met a Bonferroni corrected threshold of *P* < 5.56 × 10^−9^ (i.e. the standard 5 × 10^−8^ adjusted for nine traits): this threshold is, given the phenotypic correlations between several of the traits, somewhat conservative (Supplementary Data 2). For variants meeting the threshold, a locus was defined as ‘known’ if located within 500 kb of a signal previously associated with the respective trait, and considered ‘novel’ otherwise (Methods section). Overall, these analyses yielded 415 loci meeting genome wide significance: of these 68 loci were newly identified (Supplementary Data 2).

### Common variant meta-analysis of metabolic traits in 288 K East Asians

**Fig. 2:**
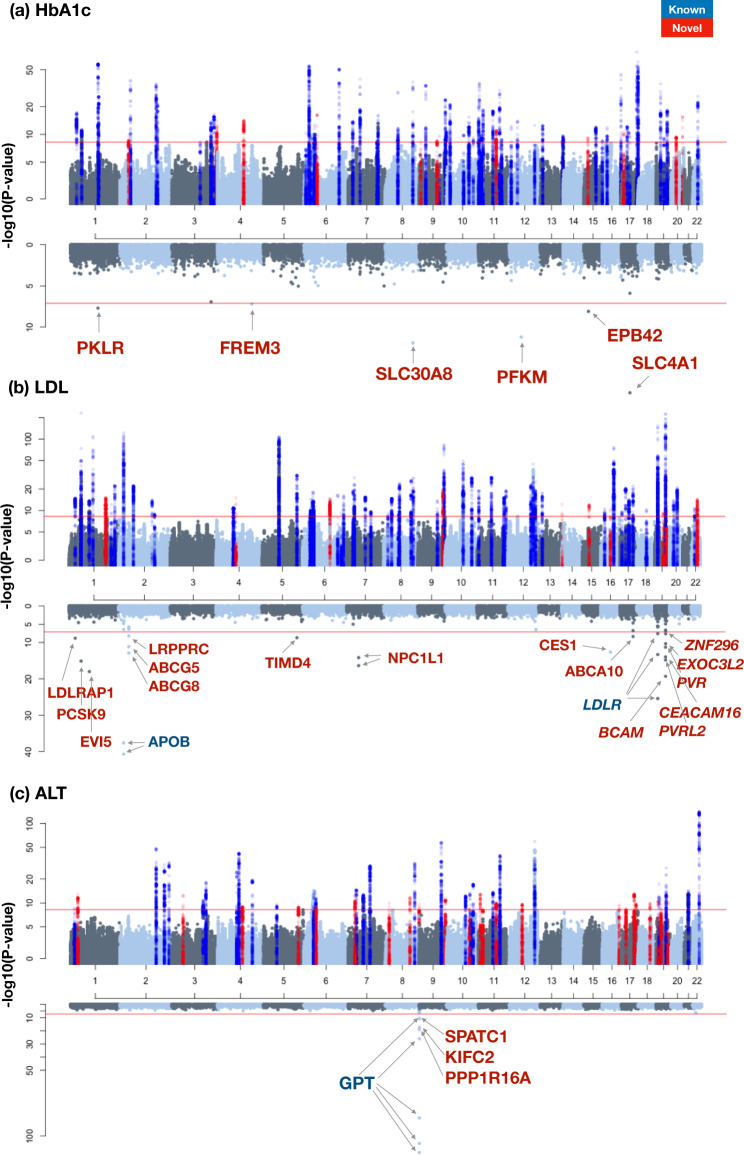
Miami plot of common and rare associations (HbA1c, LDL, and ALT). Miami plot shows linear regression analysis results of common (upper panel) and rare variants (lower panel). Red horizontal line indicates −log10(5.56e-9) and −log10(7.61e-8) for upper and lower panels, respectively. Previously known loci were colored in blue for ±250 kb of the lead variant and colored in red for ±250 kb of new associations of this study. **a** HbA1c, **b** LDL, and **c** ALT.

To boost power and seek replication, the common variant discovery was extended by combining the Korean data with summary-level information from a GWAS (6M variants after imputation) previously conducted in 162,255 individuals from BioBank Japan^14^, resulting in the largest GWAS for continuous metabolic traits in East Asians to date (*N* = 288,127). Among East Asian groups, Korean and Japan are geographically close neighbors and genetically closely related. Indeed, KBA and BBJ showed high genetic correlations (0.765 for HbA1c–0.885 for HDL; Supplementary Data 3). The meta-analysis extended the number of common variant loci from 415 to 611 (using *P* < 5.56 × 10^−9^; Fig. 2 and Supplementary Figs. 1–3), with the number of associated loci per trait ranging from 51 (ALT) to 91 (TC). Of these, 478 loci had been previously reported, leaving 133 that were novel (Supplementary Data 4). Conditional analysis of the set of 611 loci revealed a further 332 independent signals within these loci (also at *P* < 5.56 × 10^−9^) (Supplementary Data 5 and 6) for a total of 943 signals, 144 of them were novel. The calculated meta-analysis LD score regression intercept showed slight inflations ranging from *λ* = 1.02 for AST to 1.09 for HDL (Supplementary Data 6), suggesting an acceptable control on population stratification considering a large number of samples used and polygenic inheritance of the metabolic traits^36,37^. When correcting *p*-values based on genomic inflation factors, the number of common variant loci was 473 and 77 of them were novel (Supplementary Data 4).

The 144 novel signals included five instances where the lead SNP was a nonsynonymous coding variant providing a direct route to new biological inference (Supplementary Data 4). For example, a missense SNP rs1047781 (I129F) in *fucosyltransferase 2 (FUT2)* was newly associated with ALT level (MAF = 49.6%; *P* = 1.83 × 10^−12^; Supplementary Data 4). The enzyme *FUT2* is responsible for the addition of fucose to sugar moieties of glycolipids and glycoproteins by a-1,2-fucosylation^38^. Previously, Chambers et al. reported that a synonymous variant (rs281377) and an intronic variant (rs516246) in *FUT2* were associated with alkaline phosphatase (ALP) and γ-glutamyl transferase (GGT), respectively^4^. The coding rs1047781 variant has been reported to display a marginally significant association with an indicator of liver damage (AST/ALT ratio), but the association with absolute levels of AST or ALT is novel^39^. In mice, abrogation of *Fut2*^–/–^ function leads to acute liver damage and increased alkaline phosphatase, AST, and ALT levels^38^.

We further performed enrichment analysis to describe more comprehensive shape of 144 novel signals using FUMA-GWAS^40^. Enrichment of candidate genes in differentially expressed gene sets was mostly similar between known and novel loci (Supplementary Fig. 4). One notable feature was a difference in tissue specificity of known and novel loci of ALT based on enrichment analysis of differentially expressed genes in various tissues (Supplementary Fig. 4). Candidate genes from known ALT loci showed enrichment in differentially expressed gene sets in tissues of liver and small intestine. However, genes from novel loci of ALT showed enrichment in differentially upregulated gene sets of kidney medulla and cortex. Among the candidate genes of novel loci of ALT, *FUT2* secretor status was associated with self-reported kidney disease^41^. *SOX6* was reported as a modulator of renin expression in the kidney^42^. The amount of *FABP3* in urine of patients with acute kidney injury was suggested as diagnostic/prognosis marker for renal replacement therapy^43^. Patients with liver disease often complicated with kidney disease^44^. The relationship of kidney and liver is complex and underlying pathophysiology of kidney disease comorbid with liver disease is still not fully understood^44^. The functional enrichment analysis of ALT loci highlighted the possible shared genetic components of liver and kidney diseases.

We further extended replication using data from UK Biobank that had become available in the interim. UK Biobank data from 337,475 European participants were available for 121 of the 144 novel signals at the 133 novel loci. Seventy-three signals (57.0%) with consistent effect direction were nominally replicated (*P* < 0.05) in the European datasets and showed high correlation of genetic effects (overall *r* = 0.737; Supplementary Data 4 and Supplementary Fig. 5).

Some of the apparent disparities between the East Asian and European findings are likely to reflect differences in effect allele frequencies (EAF). Across the 943 signals seen in the combined KBA/BBJ analysis, EAF was highly correlated (*r* = 0.71) between EAS and EUR based on 1000 Genomes Phase 3 or gnomAD database (Supplementary Fig. 6). As might be expected, variants first identified in East Asian samples tended to show higher EAF in EAS compared to EUR (Supplementary Fig. 6). Among the 144 novel signals, 99 signals (68.8%) showed at least a 20% (relative) increase in EAF in EAS than EUR populations. Notably, there were 15 common signals from the EAS analysis (EAS MAF > 1%) that had a MAF < 0.1% in EUR, and 22 signals with EAS MAF ≥ 5% and EUR MAF < 1% in EUR (including 12 signals that were entirely monomorphic in EUR). Amongst the 48 non-replicated SNPs at the 121 novel signals for which UK Biobank data were available, 7 showed higher MAF (>5%) in EAS than Europeans (MAF < 1%).

Consistency of genetic effects and allele frequency difference among populations indicate that many of the novel associations in this study resulted from the increased statistical power offered by higher EAF in EAS including several instances of population specific alleles.

### Rare variants associated with metabolic traits in 126K Koreans

Rare variants provide an additional source of heritability for complex biomedical traits. Although numerous rare variants with large genetic effects have been described^30,45,46^, rare variant discovery efforts have tended to be underpowered (compared to common variant discovery by GWAS), and the contribution of functional rare variants to trait variance remains unclear^47^. The KBA was designed to allow genotyping of 208 K putatively functional variants (including missense, frameshift, start/stop gain or lost, splice site donor or acceptor variants) retrieved from 2572 Korean sequenced samples^33^. Of these, 68,431 of the variants with genotypes passing quality control (Methods section) were rare (MAF < 1%), and ~95% of these (64,991) were listed in the gnomAD database^48^. Association analysis of these 68,431 single rare variants (by linear regression) revealed 66 variants with significant associations (at a threshold of *P* < 8.12 × 10^−8^, that is, 0.05 adjusted for 68,431 variants and nine traits) for a total of 89 variant-trait pairs (Supplementary Datas 6 and 7). A rare variant was regarded as ‘known’ if the specific rare variant was previously reported to be associated with the same trait, and ‘novel’ otherwise. Only twelve of these associations had been described previously for the same trait (Methods section; Supplementary Data 7). Differences in MAF underlie some of these novel findings: 28 of the 66 rare variants identified in Koreans were monomorphic in Europeans (Supplementary Data 7).

For 52 of the 89 rare variant associations, previous association analyses, using exome array and/or sequencing data, have revealed variant-trait associations that implicate different coding alleles in the same genes^12,13,21,22,24,49–55^ (Supplementary Data 7 and 8). Some of the novel variant-trait associations involved variants previously discovered from sequencing based studies on related traits: for example, a variant rs730882109 (H583Y at *LDLR*; MAF = 0.02%) which was significantly associated with LDL-cholesterol in the present study, had previously been reported in a subject with hypercholesterolemia^53^. Variants rs199689137 and rs147194762, leading to missense coding changes in *ABCG5* and *ABCG8* respectively were recently discovered from sequencing data of nine Japanese families with sitosterolemia^54^.

External replication of the rare variant associations seen in the Korean study was complicated by the fact that the publicly accessible summary statistics of these traits lacked equivalent rare variant coverage: data from BBJ were limited to variants with MAF > 1% and only 21 of the 66 rare variants were present in UK Biobank exome sequencing data (*N* = 138,032) (Supplementary Data 7 and Supplementary Fig. 7). Among 89 rare associations discovered in this study, 9 associations including 4 novel were replicated (*P* < 0.05 with consistent direction of effect). Genetic effects of 21 rare variants between KBA and UK Biobank were highly correlated (*r* = 0.83; Supplementary Fig. 7). To gain further understanding of the reliability of the rare variant associations detected in KBA, we genotyped 22,608 further samples from KoGES (KBA_rep_). Overall, effect sizes were highly correlated between the discovery and replication studies (*r* = 0.97; Supplementary Fig. 7A), and 67 of the 89 variant/trait associations detected in the far larger discovery sample were replicated at *P* < 0.05 in KBA_rep_ with consistent direction of effect (Supplementary Data 7).

In all, 84 of the 89 rare variant/trait-associations mapped within 1 Mb of a previously-known or newly-associated common variant signal (74 of them within 500 kb). These findings are consistent with previous reports demonstrating that many rare variant associations occur at loci already implicated by common variant GWAS^16,17,25,56^. To exclude non-independent associations generated by closely located common and rare signals^17,56,57^, sets of common and rare associations within 1 Mb apart (i.e., within the same “co-incident” locus (CL), see Methods section) were jointly analyzed by multiple linear regression. Across a total of 46 such CLs, there were 125 common lead and rare variants to be considered (Supplementary Data 9), and, for most, (86 of 125 [68.8%]) conditional analyses indicated independence (<10% reduction in effect size on conditional). Fourteen variants (one common; 13 rare) showed a 10–30% reduction in effect size when conditioned on other nearby lead variants, and for a further 16 (implicated in 22 rare variant associations) the reduction exceeded 30% (Supplementary Data 10). Amongst the latter group of 16 unique rare variants, 8 were annotated as damaging, and 8 as benign (dbNSFP v2.9) (Supplementary Data 10). The *APOE* region (CL#25) provides an example of such dependent associations across a set of 2 common (rs429358 and rs7412) and 6 rare lead variants: the signals for all six rare variants were drastically diminished after conditional analyses (Supplementary Datas 8 and 10), an inference supported by haplotype analysis (Supplementary Data 11 and 12).

We explored known clinical consequences of the rare variant associations detected using ClinVar database^58^, finding entries for 22 variants, nine annotated as benign (or likely benign), six as of uncertain significance, and five pathogenic (two returned conflicting interpretations of pathogenicity; Table 1). The rare variant associations observed in our study can provide additional evidence to support ClinVar interpretation. For example, ClinVar considers that a rare variant rs104894487 (A142T at *EPB42)* may be related to hereditary spherocytosis based on ‘uncertain significance’ annotated by one submitter and ‘pathogenic’ by two others^59^. The rare allele association at *EPB42* in this study involved reduced levels of HbA1c, consistent with the reduced red cell half-life seen in patients with hereditary spherocytosis^60^.

**Table 1:** Rare associations annotated in ClinVar database

CHR	POS	Ref/Alt	Gene	MAF	Trait	Effect	SE	*P*-value	Clinvar var. ID	Protein change	Molecular consequence	Condition	Class
1	55,523,798	A/G	PCSK9	0.0032	LDL	−0.2933	0.0364	7.26E-16	630597	I424V	Missense	Familial hypercholesterolemias	Benign
					TC	−0.2642	0.0358	1.69E-13					
1	155,261,697	G/A	PKLR	0.0055	TG	0.1489	0.0277	7.58E-08	292806	R490W	Missense	Pyruvate kinase deficiency of red cells	Uncertain significance
1	155,263,025	A/G	PKLR	0.0019	HbA1c	−0.4154	0.074	1.97E-08	225440	V460A	Missense	Pyruvate kinase deficiency of red cells	Uncertain significance
2	21,228,437	A/G	APOB	0.001	LDL	−0.8857	0.0657	1.97E-41	630249	I3768T	Missense	Familial hypercholesterolemias	Uncertain significance
					TC	−0.7885	0.0651	1.00E-33					
2	44,050,063	G/A	ABCG5	0.0012	LDL	0.4076	0.0594	6.80E-12	30485	R446*	Nonsense	Sitosterolemia	Pathogenic
					TC	0.3758	0.0588	1.63E-10					
2	44,100,999	A/G	ABCG8	0.0074	LDL	0.1784	0.0241	1.25E-13	499929	M429V	Missense	–	Uncertain significance
					TC	0.1518	0.0238	1.81E-10					
**2**	**44,116,923**	**C/T**	**LRPPRC**	**0.0091**	**LDL**	**0.1261**	**0.0217**	**6.07E-09**	**746339**	**A1360T**	**Missense**	–	**Benign**
**9**	**107,560,803**	**C/T**	**ABCA1**	**0.0076**	**HDL**	**−0.1447**	**0.0235**	**7.50E-10**	**364396**	**V1674I**	**Missense**	**Tangier disease, Familial High Density Lipoprotein Deficiency**	**Benign**
					**TC**	**−0.1317**	**0.0235**	**2.14E-08**					
9	107,584,945	C/A	ABCA1	0.0061	HDL	−0.2315	0.0266	3.64E-18	225290	C887F	Missense	Familial hypercholesterolemia 1	Uncertain significance
					TC	−0.1815	0.0265	7.96E-12					
10	101,165,607	T/C	GPT	0.0032	AST	−0.2246	0.04	1.90E-08	709106	E183G	Missense	–	Benign
11	116,701,560	G/A	APOC3	0.0008	HDL	0.8077	0.0755	1.02E-26	139561	A43T	Missense	Apolipoprotein C-III deficiency, Coronary heart disease	Pathogenic
					TG	−0.8409	0.0761	2.39E-28					
11	116,703,580	A/G	APOC3	0.0017	TG	−0.3203	0.0492	7.73E-11	17902	T74A	Missense	Apolipoprotein c-iii, nonglycosylated	Pathogenic
15	43,507,389	C/T	EPB42	0.0028	HbA1c	−0.3386	0.0587	7.88E-09	13233	A142T	Missense	Spherocytosis type 5	Pathogenic
16	56,917,997	C/T	SLC12A3	0.0022	HDL	0.2593	0.0443	4.97E-09	225468	A569V	Missense	Familial hypokalemia-hypomagnesemia	Uncertain significance
16	56,918,023	G/A	SLC12A3	0.0047	HDL	−0.1608	0.0297	5.91E-08	225469	V578M	Missense	Familial hypokalemia-hypomagnesemia	Benign
16	57,016,150	G/A	CETP	0.0005	HDL	1.3379	0.0877	1.60E-52	17524	-	Splice donor	Hyperalphalipoproteinemia 1	Pathogenic
17	41,246,724	C/T	BRCA1	0.0023	TG	0.3234	0.0429	4.72E-14	55726	G275D	Missense	Breast-ovarian cancer, familial 1, Hereditary cancer-predisposing syndrome, Neoplasm of the breast, AllHighlyPenetrant	Benign
19	11,217,315	C/T	LDLR	0.0002	LDL	1.0777	0.1429	4.59E-14	251446	R257W	Missense	Familial hypercholesterolemia	Conflicting interpretation
					TC	0.9784	0.1374	1.07E-12					
19	11,227,576	C/T	LDLR	0.0002	LDL	1.5104	0.1428	3.80E-26	200921	H583Y	Missense	Familial hypercholesterolemia	Conflicting interpretation
					TC	1.2383	0.1428	4.29E-18					
19	11,241,988	C/T	LDLR	0.0014	LDL	−0.3384	0.0607	2.49E-08	374957	A860V	Missense	Familial hypercholesterolemia	Benign
**19**	**45,207,444**	**C/T**	**CEACAM16**	**0.009**	**LDL**	**−0.1694**	**0.0219**	**1.02E-14**	**226509**	**S180F**	**Missense**	**–**	**Benign**
20	43,042,364	C/T	HNF4A	0.0084	HDL	−0.1451	0.0228	2.07E-10	129240	T114I	Missense	Maturity onset diabetes mellitus in young, Hyperinsulinism, Mongenic diabetes	Benign

Chromosomal positions are based hg19. Effect size is based on alternative allele. ClinVar database was assessed at 1st May 2020. From the conditional analysis results, attenuated rare variants are bold-faced.

*CHR* chromosome, *POS* position, *MAF* minor allele frequency.

### The interplay of common and rare variants in relation to the genetic risk score

Genetic risk scores (GRS) summarize the contribution of genome-wide association signals on individual phenotypic variance^8^, and have potential for preventive intervention, lifestyle modification, and clinical decision making^8^. For each metabolic trait, we calculated CV-GRSs using the sets of common lead variants significantly associated with each trait from the KBA/BBJ meta-analysis (and taking effect sizes from the same). To reduce overfitting of CV-GRS when applied to discovery samples, the evaluation of CV-GRS performance was restricted to the 23 K samples from the KBA_rep_ replication cohort. As expected, trait CV-GRS showed strong associations with their respective phenotypes (Supplementary Data 13 and 14): trait variance explained increased by 1.5% (for ALT) to 10.2% (for HDL) when CV-GRS was added to a model using only covariates including age, sex, and recruitment area (Supplementary Data 13). Individuals with GRS measures at the upper end of the distribution had metabolic trait values consistent with future health risk. For example, mean HbA1c of the top 1% of HbA1c GRS was 5.75%. The top 10% risk group prefigured future diabetes considering prediabetic condition defined with FPG measures of 110–125 mg/dL, HbA1c of 5.7–6.4%^61^. Also top 1% of lipids GRS showed an elevated mean level of lipids close to dyslipidemia (satisfying one of the following: TC ≥ 240 mg/dL, LDL > 160 mg/dL, TG > 200 mg/dL, or decreased HDL < 40 mg/dL)^2^, an indicative of elevated cardiovascular risk (Supplementary Data 15).

Rare variants with comparatively large effects on trait measures have the potential to improve the performance of GRS, in some individuals at least. We generated ALL-GRS scores by adding, to the CV-GRS, only those rare variants that had been demonstrated, based on conditional analysis, to be independent of nearby common variants (Supplementary Data 7 and 9).

The performance of the ALL-GRS was only marginally better than the equivalent CV-GRS in both discovery and replication studies (Supplementary Data 14 and 16): in KBA_rep_, the increase in trait variance explained was <1% (Supplementary Data 14). This reflects the relatively small proportion of individuals who carry trait-associated rare alleles (for example, for HbA1c, 0.54% of the 125,872 individuals in the discovery sample: for LDL, 7.96%). This limits the impact of the rare alleles on GRS performance even though associated rare variants had effect sizes that were on average nine times greater than common variants overall (and five times greater when compared to common alleles at the same locus; Supplementary Data 4 and 7).

An obvious limitation of population-level comparisons between the performance of the CV-GRS and ALL-GRS is that coverage of the rare variant space was, for a variety of reasons including pre-defined array content and sample size, far less comprehensive than that of the common variant contribution to trait variation. An alternative approach for gauging the impact of rare variants concentrates on their impact on common variant polygenic risk in the subset of individuals that are carriers^8^. To study the interplay of rare alleles and common variant polygenic effects (as measured by the CV-GRS), samples were grouped into four categories based on the direction of rare allele effects (Supplementary Data 17 and Supplementary Fig. 8). The first group included individuals from KBA who carried one or more rare alleles associated with improved health (that is, decreasing levels of traits other than HDL): the proportion of the sample ranged from 1.0% for AST to 3.5% for TC (there were no such carriers for GGT). The second group comprised KBA individuals carrying only one or more rare alleles associated with reduced health: these constituted from 0.3% for FPG to 5.8% for LDL (none for HbA1c, ALT, and AST). The third group of individuals carried a mixture of rare alleles which (for a given trait) had opposing effects: this was a small group constituting 0.01% for FPG to 0.15% for HDL (and none for HbA1c, ALT, AST, and GGT). The remaining group carried no rare associated alleles: this reference group ranged from 92.04% of the sample for LDL to 99.29% for GGT).

Trait levels were decreased (or, in the case of HDL, increased) between 2% (HbA1c) and 28% (ALT) in the first group (as compared to the reference group, and increased (HDL, decreased) between 3% (FPG) and 22% (TG) in the second group (Supplementary Data 17). Similar patterns were observed in the KBA_rep_ dataset (23 K samples) (Supplementary Data 17). These effects resulted in redistribution of some individuals assigned high disease risk on the basis of their CV-GRS measures. For example, the proportion of dyslipidemia based on TG level (TG > 200 mg/dL) for individuals in the top decile (mean TG = 170.2 mg/dL) of the CV-GRS for TG was 26.1% while the proportion was increased to 35.7% in the subset with TG-raising rare alleles (mean TG = 236.2 mg/dL; Supplementary Data 17). This illustrates the impact of rare alleles (that typically go unmeasured using array based approaches) on the performance of GRS that are based on common variants alone.

### Inherited risk of glycemic traits and relation to T2D

Individuals with GRS measures associated with adverse metabolic profiles (e.g., high glucose or cholesterol) are likely to show increased susceptibility to trait-related diseases such as diabetes or coronary artery disease. We explored this further in the KBA data, focusing on the relationship between glycemic traits (FPG; HbA1c) and T2D (Methods section).

**Fig. 3:**
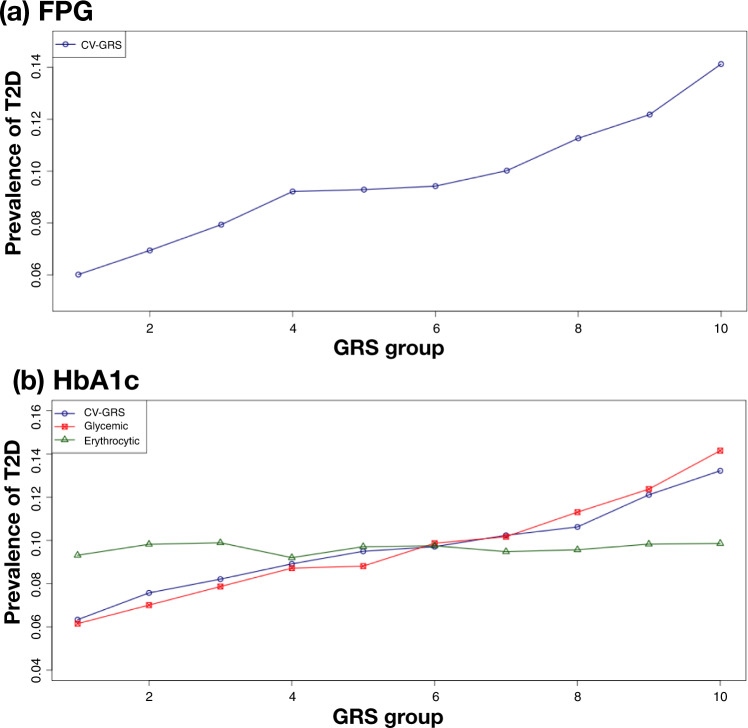
Prevalence of type 2 diabetes by GRS group. Samples were grouped into 10 groups based on GRS scores in an increasing order. CV-GRS indicates GRS using common lead variants identified in this study. For each GRS bin, T2D prevalence was calculated as # of T2D samples divided by # of samples in the GRS bin. **a** FPG and **b** HbA1c.

In the KBA GWAS, glycemic trait analyses had been restricted to individuals without diabetes, allowing us to examine the impact of the glycemic GRS (Methods section; Supplementary Data 1) on T2D prevalence across the entire 126 K samples of KBA (which included 12,135 cases of T2D). Both the FPG and HbA1c GRSs were strongly associated with T2D (FPG-GRS: OR (per SD of the GRS) = 1.46, *P* = 3.21 × 10^−300^; HbA1c-GRS: OR = 1.35, *P* = 4.95 × 10^−194^, Supplementary Data 18). Previous evidence indicates that the genetic contribution to variation in HbA1c can be decomposed into glycemic and erythrocytic components, the latter acting through effects on red-cell longevity^15^: as expected, only variants implicated in the former contributed to T2D prevalence (OR = 1.43, *P* = 1.03 × 10^−268^; Methods section, Supplementary Data 19 and Fig. 3). Moreover, classification performance by area under the curve (AUC) of glycemic and erythrocytic components further support contribution of glycemic components to T2D prevalence. The AUC was 0.60 and 0.51 for glycemic and erythrocytic components, respectively. When mean levels of glycemic traits were plotted along with GRS, there were little change of mean FPG level among bins of HbA1c GRS using only erythrocytic components (Supplementary Fig. 9).

**Fig. 4:**
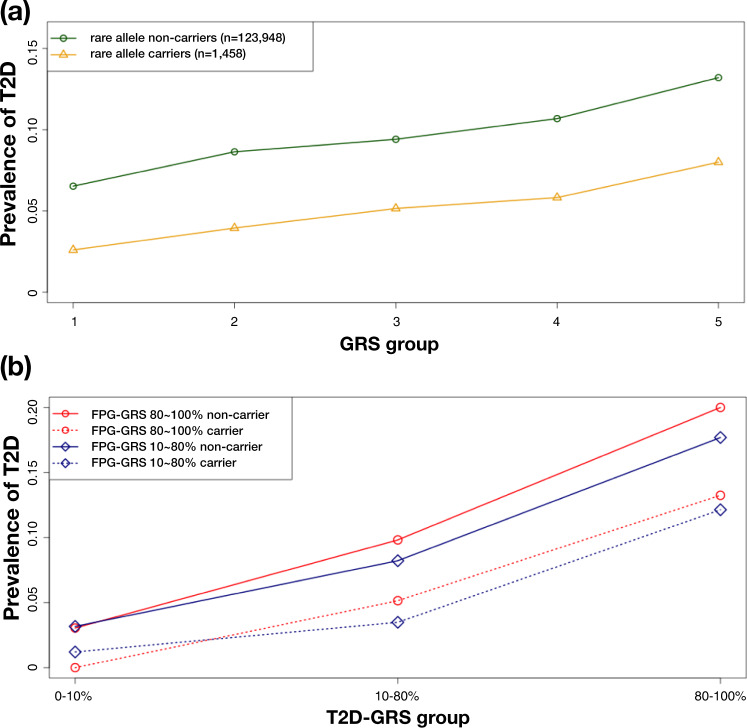
Interplay of common and rare variants in inherited risk of T2D. After sorting CV-GRS scores in an increasing order, CV-GRS bins were categorized as 1st bin (1–20%), 2nd bin (21–40%), 3rd bin (41–60%), 4th bin (61–80%), and 5th bin (81–100%). For rare allele carriers and non-carriers, all samples of KBA were categorized into five CV-GRS bins and T2D prevalence was calculated for rare allele carriers and non-carriers, separately. **a** T2D prevalence by FPG-GRS and a rare allele of *SLC30A8*, **b** T2D prevalence by T2D-GRS, FPG-GRS, and a rare allele of *SLC30A8*.

To gain further insights we focused on the CV-GRS for FPG (hereafter, FPG-GRS), grouping all 126 K genotyped KBA subjects into 10 bins based on their FPG-GRS. T2D prevalence ranged from 6.0% in the lowest FPG-GRS bin, to 14.1% in the highest (Fig. 3a). We considered the impact of adding genotype data for the four rare variants with a significant association with FPG in this population (Supplementary Data 7). These variants were tested for an association with T2D (12 K cases and 94 K controls). Of these, only the coding variant at rs770224130 (I349F in *SLC30A8*) (MAF = 0.6% in KBA, monomorphic in Europeans from gnomAD) was associated with T2D (OR = 0.403, *P* = 1.11 × 10^−16^; Supplementary Data 20). Carriers of the protective rare allele at this variant had a T2D prevalence in KBA of 4.9% (compared to 9.6% among the full set of 126 K samples in KBA), equivalent to that of the lowest FPG-GRS group. Not surprisingly therefore, adding the *SLC30A8* variant genotype to the FPG-GRS predictions had a marked impact (Table 2 and Fig. 4a): for example, the T2D prevalence for the top decile of the FPG-GRS fell from 14.2% overall to 7.3% in carriers; and from 6.1% to 3.7% in the bottom decile (Table 2 and Supplementary Fig. 10).

**Table 2:** Prevalence of T2D in GRS groups stratified by the presence of a rare protective allele

GRS type	Group	Non-carrier		Carrier of a rare protective allele		Odds ratio	95% CI	*P*-value
		*N*	T2D prevalence (%)	*N*	T2D prevalence (%)			
–	All samples	123,948	9.7	1458	4.87	0.398	0.31-0.51	1.42E-13
FPG	Top 10%	12,451	14.18	96	7.29	0.338	0.15-0.76	8.16E-03
	Bottom 10%	12,367	6.05	164	3.66	0.512	0.22-1.17	1.13E-01

For GRS groups, T2D prevalence was calculated for non-carriers and carriers of a rare protective allele. For each GRS group, a logistic regression model was used to test an association between T2D and a rare protective variant adjusted for age and sex.

We next generated a T2D-GRS from the KBA data using previously reported variants^17,62^ and applying effect sizes estimated in this study (Supplementary Data 21). This T2D-GRS was, as expected, more predictive of T2D prevalence than the FPG-GRS (though this may in part reflect overfitting of the score; Supplementary Data 22). The combination of FPG-GRS and T2D-GRS was more powerful than either alone, with individuals in the top 10% of both scores showing ~5-fold increase in T2D prevalence compared to median group (40–60%) in both FPG-GRS and T2D-GRS, and those in the top percentile showing ~16-fold increase (Supplementary Data 22). Amongst individuals in the top quintile for both FPG-GRS and T2D-GRS, T2D prevalence was 20%, but only 12% in carriers of the protective *SLC30A8* allele (Fig. 4b). Thus, analyses based on this single rare protective variant, present in about 1% of Koreans, illustrate how the performance of GRS constituted from common variants alone cannot be relied upon to provide robust disease prediction in carriers of impactful rare alleles^8,32^ (many of whom, of course, will not be identified as such based on common variant focused analysis).

## Discussion

In this study of 288,127 East Asian subjects from Korea and Japan, we identified over 1000 common and rare variant associations across nine metabolic traits (Supplementary Datas 2–7), many of them novel. Since most GWAS data have been generated in individuals of European descent, these data build understanding of variants influential in East Asians, and contribute to efforts to develop clinical genomic tools that can be used in diverse populations^10^.

We used these data to explore the contributions of common and rare variants to trait variance (defined using a threshold MAF of 1%), demonstrating that whilst GWAS-captured common variants dominate at the population level, the greater effect size evident from some trait-associated rare variants can translate into marked impact in a subset of individuals. We illustrate this interplay between common variant polygenic scores and rare variants through the effects of a protective rare allele at *SLC30A8* on T2D risk across strata of polygenic risk predictions.

We also tested the transferability of GRS by employing genetic effect sizes derived from East Asians and Europeans using commonly available variants of KBA, BBJ, and UKB (Supplementary Data 23). Our study demonstrated that CV-GRS using effect sizes from KBA showed greater variance explained over those of KBA + BBJ meta-analysis and Europeans when applied to KBA yet CV-GRS based on KBA + BBJ and trans-ethnic meta-analysis showed comparable performance. This result suggested that local ancestry within East Asia would affect the performance of CV-GRS more than an increase of sample size in meta-analysis comprising genetically closely related local ancestries. The variance explained of CV-GRS when applied to UKB was the largest using effect sizes from UKB followed by the comparable performance from trans-ethnic CV-GRS, further supporting the use of the results from genetically close samples. Taken together, these results implied that GRS can be reliably constructed based on summary statistics from close ancestries or a large-scale trans-ethnic meta-analysis.

The PRS approach using genome-wide variants would increase the variance explained for traits. We performed PRS analysis for nine metabolic traits and T2D and compared the performance of PRS compared to those of CV-GRS. For metabolic traits, PRS showed increased variance explained of ~4% (14.3%) for HDL compared to CV-GRS (10.2%) in the replication study (Supplementary Data 24). However, PRSs for other traits showed comparable performance to those of CV-GRS (Supplementary Data 14). In addition, when T2D PRS was applied to the analysis summarized in Table 2 and Fig. 4b, the results of T2D PRS showed similar results to those of T2D-GRS (Supplementary Data 24 and Supplementary Fig. 11). Although limited increment has been shown in performance of PRS compared to those of GRS using top signals^63^, a thorough PRS analysis is warranted for various traits and analytic methods as shown a large increment in variance explained for HDL in this study.

The heterodimer of *ABCG5* and *ABCG8* is known to play a crucial role in cholesterol secretion^64^. Recent crystal structure of *ABCG5*/*ABCG8* heterodimer suggested that interaction between the extracellular domain helices of both proteins is important for sterol exit from the transmembrane domains^65^. A nonsense mutation R446Ter in *ABCG5* detected for association with LDL and TC in our study generates truncated protein resulting in the missing of the extracellular domain helix. We believe that this mutation disrupts the heterodimer formation between *ABCG5* and *ABCG8*, ultimately resulting in the accumulation of cholesterol. The functional relevance of a missense mutation M429V in *ABCG8* to the cholesterol secretion is not clear on the basis of *ABCG5*/*ABCG8* heterodimeric structure as exemplified in the ClinVar record, in which this mutation indicated uncertain significance on the certain clinical condition (Table 1). However, this mutation strongly associated with LDL and TC in our study and also replicated in UKB exome sequencing data, implying certain steric alteration in the heterodimer caused by this amino acid substitution.

One of the obvious limitations of this study is that our ability to survey rare variation across the genome was constrained both by the content of the array, and the sample size. The latter restricts rare variant association discovery to alleles of relatively large effect. Moreover, haplotype phasing using common and rare variants in this study would be less accurate considering sparse number of genotyped variants and limited number of rare variants compared to those of sequencing data. Therefore, careful interpretation is required. Whole genome sequencing approaches deployed on cohorts of this scale (or larger) are required to extend the range of rare variants that can be robustly implicated in trait variation. In addition, the KBA study was best-suited to the study of quantitative traits, given case numbers for most diseases were relatively low. The future of studies like this will rely on the integration of data across multiple large biobanks, a process we were able to initiate through combining data from Korea with similar data from Japan and the UK.

Taken together, the present study provides new insights into the architecture of trait variation in East Asian populations, documents the interplay of common and rare variants that contribute to genetic predisposition to disease, and highlights the value of rare functional variants to promote novel therapeutic strategies.

## Methods

### Study subjects

This study was approved by the institutional review board of the Korea Disease Control and Prevention Agency, Republic of Korea. The Korean Genome and Epidemiology Study (KoGES) was initiated in 2001 to investigate the genetic and environmental factors responsible for complex diseases in Koreans. A detailed description of the KoGES has been previously reported^34^. In the three population-based cohorts, 10,030, 173,357, and 28,338 participants were independently recruited from the KoGES_Ansan and Ansung study, the KoGES_health examinee (HEXA) study and the KoGES_cardiovascular disease association study (CAVAS), respectively. All participants (aged 40–70 years) provided written informed consent and were examined through epidemiological surveys, physical examinations, and laboratory tests.

Blood biochemical quantitative traits were measured for glycemic traits (FPG, HbA1c, and a-2h on oral glucose tolerance test(OGTT)), plasma lipids (HDL, LDL, TG, and TC), and liver enzymes (ALT, AST, and GGT). However, the OGTT trait was not analyzed because only ~5% of the total KBA samples (6,483 samples in the KoGES_Ansan and Ansung study) were available. Friedewald’s formula was used to calculate the LDL concentration^66^. Individuals receiving ongoing medication or therapy with a high probability of influencing metabolic traits, were excluded from the analysis (Supplementary Data 1). The basic characteristics of the traits are summarized in Supplementary Data 1.

### T2D phenotyping

T2D cases were defined based on the American Diabetes Association (ADA) criteria: a FPG concentration ≥ 126 mg/dL (7.0 mmol/L), OGTT ≥ 200 mg/dL (11.1 mmol/L), or a HbA1c ≥ 6.5% (48 mmol/mol). Participants with a past diagnosis based on self-report questionnaires were also included in the patient group. Based on the self-reported questionnaire, a control group was selected based on the following ADA criteria among subjects with no diagnosis of diabetes considering availability of variables among participants: a FPG concentration <100 mg/dL (5.6 mmol/L), a OGTT < 140 mg/dL (7.8 mmol/L), or a HbA1c level <6% (42 mmol/mol). There were 12,135 T2D cases and 94,636 controls.

### Genotyping and quality control

The Korea National Institute of Health launched the KBA project in 2014. Briefly, more than 95% of the KBA content consisted of ~600 K tagging variants for genome-wide coverage and ~208 K functional variants including missense variants, expression quantitative trait loci (eQTL), and indels retrieved from 2579 sequenced Korean samples consisting of 397 samples with whole genome sequencing and 2182 samples with exome sequencing data^33^.

All participant samples collected by KoGES and stored in the National Bank of Korea (NBK) were genotyped using KBA v1.0 (Kv1.0) and KBA v1.1 (Kv1.1). Kv1.0 (833 K SNPs) and Kv1.1 (827 K SNPs) share ~93% of its contents^33^. At the end of 2017, a total of 134,721 samples were produced: 51,963 (38.6%) for Kv1.0 and 82,758 (61.4%) for Kv1.1.

Considering the genotyping platform and enrollment information such as the year and site, ~3000–8000 samples were grouped into batches for genotype calling. Genotypes were called per each batch and quality control (QC) of the samples and SNPs was conducted in batches. Plink v1.9 software was used for handling binary formatted plink files^67^. Quality control was conducted as follows in a step-by-step manner: (1) samples QC: exclusion of gender inconsistency (*n* = 70, ~0.05% of initial 134,721 samples), low call rate (<97%) or excessive heterozygosity (HET) based on all variants on the array (HET < 0.17 or HET > 0.19 for Kv1.0 and HET < 0.15 or HET > 0.17 for Kv1.1; *n* = 1160), and outliers (PC1 > |0.1| or PC2 > |0.1|, *n* = 43) of the principle component analysis results using FlashPCA2^68^. Furthermore, by analyzing all the batches together, 2nd-degree relatives were removed to secure unrelated genotype data for further analysis (*n* = 7576). KING v2 was used to inferring 2^nd^-degree relatives using overlapped variants between Kv1.0 and Kv1.1^69^. All QCed batches were then combined in Kv1.0 (*n* = 48,005) and Kv1.1(*n* = 77,867). (2) SNP QC (per batch): exclusion of poorly clustered SNPs based on the SNPolisher analysis results, missing rate > 5%, and HWE failure *P* < 10^−6^.

For the combined Kv1.0 and Kv1.1 data, the QC of common (MAF ≥ 1%) and rare variants (MAF < 1%) was performed separately. For common variants, SNPs were further excluded if the missing rate was >10%, allele frequency difference was >0.2 when compared to 1000 Genomes Project Phase 3 East Asians (*n* = 504) or Korean Reference Genome (*n* = 397), MAF < 1%, and HWE failure *P* < 10^−6^. Consequently, 549 K SNPs (Kv1.0) and 518 K SNPs (Kv1.1) were retained for phasing and imputation analysis.

In SNP microarray, genotype calling of rare variants is challenging because only a small proportion of samples are heterozygous. Although KBA contains high quality rare variants with a high score of quality metrics from the genotype clustering analysis, poor genotype clusters may mislead the analysis results and impede following interpretation. Therefore, we further excluded putative poorly clustered rare variants based on allele frequencies from East Asians in the gnomAD database^48^ and 2579 sequenced Korean samples^33^. In total, 163,026 functional variants (missense, frameshift, start/stop gain or lost, splice site donor or acceptor, and structural interaction) were available based on the 48,005 samples of Kv1.0 dataset and 77,867 samples from the Kv1.1 dataset. After combining all 153 K variants of Kv1.0 and Kv1.1, the putative poorly clustered rare variants were further excluded in a step-by-step manner. First, allele frequencies of rare variants were calculated for each batch. Second, for each rare variant, genotypes of the samples in a batch were set to missing if the difference in the allele frequency of a rare variant in the batch was more than 0.005 (0.5%) compared with the mean allele frequency of the remaining batches. Variants were excluded based on the following criteria: MAF > 1%, minor allele count (MAC) < 30, HWE *P* < 10^−6^, or missing rate >30%. In our dataset, variants with a MAC < 30 threshold showed more unclear cluster plots with less than 30 points in the heterozygote cluster compared to the variants with MAC ≥ 30. For a missing rate of rare variants, the threshold was eased because the missing rate was mainly based on a batch effect and not by technical errors such as obscure genotype clustering. For the remaining rare variants, the MAF of rare variants was compared to that of 2579 sequenced Korean samples, 504 East Asians from the 1000 Genomes Project Phase 3, and 9435 East Asian samples from the gnomAD database. Finally, we selected only rare variants with MAF differences of <0.5% between the 125,872 KBA data samples and either of 2579 sequenced Korean samples, 9,435 East Asian samples from the gnomAD database, and 504 samples from the 1000 Genomes Project Phase 3 (Supplementary Fig. 12). As a result, 68,431 rare functional autosomal variants were included for further analysis. Overall, we observed a high correlation (*r* = 0.917) of the MAF for 68,431 rare variants between the 125,872 samples and 2579 sequenced Korean samples. Given the recent concerns over rare variants directly genotyped using microarray^70^, allele frequencies and cluster plots were reviewed prior to the post-association analysis. After performing association tests for rare variants, cluster plots per batches were visually inspected, batches with poor cluster plots were manually removed if needed (19 variants), an association analysis was performed for these additionally QCed variants. Among the associated rare variants, two variants showed poor cluster plots and were excluded from further analysis. The cluster plots of the associated rare variants are shown in Supplementary Fig. 13.

### Replication study (UK Biobank)

The UKB provided genotype data for over a half million samples with deep phenotyping and molecular data^11^. Related information on the genotyping, QC, and imputation analysis has been previously reported^11^. Among the QCed and imputed data, we removed individuals with non-European ancestry and non-independent samples using Data-Field 22006 and 22020. As a result, 337,475 individuals were included for further analysis. Samples with diseases or taking medications that likely influenced the biochemical traits were removed using Data-Field 2443, 4041, 6153, 6177, and 41202. Biochemical traits were filtered and transformed according to the methods of KBA described in Supplementary Data 1. For the association analyses, SNPTEST v2.5.2 was used for imputed variants with high imputation quality (INFO ≥ 0.8). All analyses were conducted under the UKB application 57705.

For replication study of rare variants, about 200 K exome sequencing data of UKB was analyzed^71^. Among 200 K samples, there were 138,032 samples available with any of nine metabolic traits among the genotyped samples (*n* = 337,475) used for replication study of common variants. In all, 21 of 66 rare variants discovered in this study were available after excluding variants with MAC ≤ 2, missing rate > 5%, and HWE *P* < 10^−6^. Associations between rare variants and the transformed traits were performed using EPACTS v3.4.6.

### Replication study (KBA_rep_)

In 2018, ~24,000 samples from the HEXA cohort were genotyped using the KBA. The QC procedures for samples and SNPs were were performed as described above for genotying and QC. As a result, 22,608 samples were remained and the variants discovered in this study were assessed for the replication analysis. Common variants were imputed if they were not directly genotyped. Cluster plots of rare variants were shown in Supplementary Fig. 14.

### Functional annotation

The functional category was annotated using SnpEff and SnpSift based on the dbNSFP v2.9 database^72–74^. Known associations for metabolic traits were retrieved from the GWAS Catalog (as of January 2021)^75^ and the recently published GWAS literatures.

### Genotype imputation

Eagle v2.3 was used for the phasing of the QCed data^76^. Impute v4 was used for imputation analysis using a merged reference panel from 2504 samples of 1000 Genomes Phase 3 and 397 samples from the Korean Reference Genome^11^, and QCTOOL v2 was used to calculate the imputation quality score and info values (see URLs). Imputed variants with info <0.8 or MAF < 1% were excluded and approximately 8.3 M variants were used for further analysis. The imputation output GEN formatted file was converted to VCF format with imputed dosages by using GEN2VCF^77^.

### Co-incident locus

Rare and common variants from the lead signals in this study or previously reported lead variants (*P* ≤ 10^−5^ in this study), which were used if a lead variant was not obtained from the discovery study, were clustered if they were located within 1 Mb window. As a result, 46 co-incident loci (CL) were defined, and they included 58 unique rare variants (81 associations) and 44 common variants. However, eight rare variants were not included in the CLs: (1) absence of nearby common or rare associations within 1 Mb (*n* = 4), (2) previously reported common signals were within 1 Mb yet not significant (*P* > 10^−5^; *n* = 3), and (3) known common lead signal from the previous GWAS with European ancestry (*n* = 1). For the *APOE* region, a common variant rs429358 was added along with the lead signal rs7412. These two variants are well known to produce three major *APOE* alleles^78^.

### Haplotype based association analysis

For the CL, all variants ±200 kb in the region were phased using Eagle v2.3^76^. Phased haplotypes were then parsed to extract information on the target variants of the region. The most frequent haplotype was regarded as a reference and less frequent haplotypes were tested for an association based on comparison with a reference. Multiple linear regression analysis was performed to test the independent association of haplotypes by jointly testing all the haplotype variables.

### Calculation of genetic risk score

Using the lead common variants and rare variants discovered in this study, the GRS was calculated for each sample based on the sum of the number of risk alleles weighted by the effect size of the associated variant. For each trait, the GRSs of all samples were transformed to follow the standard normal distribution.

### Calculation of polygenic risk score

For metabolic traits, we adopted a tenfold leave-one-group-out (LOGO) meta-analysis method^79^ since the variance explained from LOGO was greater in overall than those of PRS based on BBJ (BioBank Japan) summary statistics. For example, FPG PRS based on BBJ showed 1.9% of variance explained (VE) while the GRS from LOGO showed 5.5%, possibly caused by differences in the recruitment policy (hospital-based in BBJ and population-based in KBA). 126 K individuals of KBA were divided into ten subgroups to perform a GWAS for each subgroup on each trait. PRS-CS was used for PRS analysis using only HapMap phase 3 variants (about 970 K variants)^80^. Next, meta-analysis was performed using GWASs of nine subgroups and adjusted weights were obtained by PRS-CS from the meta-analysis results. Then PRS was calculated for one remaining group using the adjusted weights. These procedures were repeated to calculate PRS for all 126 K individuals for all traits. For T2D PRS, adjusted weights were estimated from BBJ T2D GWAS^62^. This T2D-PRS (VE = 9.6%) showed better performance than LOGO in KBA (VE = 7.9%). Since KBA was included in the recently published East Asian T2D GWAS^81^, we did not used summary statistics from Spracklen et al. to avoid overfitting problem.

### Classification of HbA1c associated variants into glycemic and erythrocytic variants

HbA1c associated variants were classified into three groups: (1) ‘glycemic’ if the variant was associated with FPG or T2D in this study or reported in previous studies (*P* < 1 × 10^−4^), (2) ‘erythrocytic’ if the variant was associated with hemoglobin, MCH, MCV, RBC, or MCHC (*P* < 1 × 10^−4^) based on the available summary statistics of BBJ^14^, and (3) ‘unclassified’ otherwise.

### Statistical analysis

For each genotyping platforms (Kv1.0 and Kv1.1), QCed genotypes were imputed by platforms as described above. A GWAS was conducted using the imputed genotypes by the platforms. For the association analysis, residuals were obtained from a linear regression model of the measured value or common log-transformed value of all traits after adjusting for age, sex, and recruitment area. The residuals were transformed to approximate a normal distribution (Supplementary Data 1). Single variant association analysis (linear regression) on the transformed traits was performed using EPACTS v3.4.6 assuming an additive mode of inheritance based on the alternative allele count. The KBA GWAS was conducted via meta-analysis based on a combination of the Kv1.0 and Kv1.1 summary statistics. Then, a meta-analysis of the summary statistics of the KBA and BBJ was performed. Inverse variance weighted meta-analyses were performed using METAL software^82^. Associated variants (*P* ≤ 5.56 × 10^−9^) were clustered as a locus if the variants were located within a 500 kb range. Independently associated loci were defined if the minimum distance between any distinct locus was greater than 500 kb. Common associations were regarded as ‘known’ if the distance was <500 kb from the previous associations, and ‘novel’ otherwise. Most of the length of defined loci were <2 Mb except for the *APOB* region on chromosome 2, the human Leukocyte antigen region on chromosome 6, and the 12q24 region of the well-known long-range haplotype^83–85^. Conditional analyses of the GWAS summary data were performed using GCTA-COJO software (Genome-wide Complex Trait Analysis, conditional & joint association analysis)^86^ to identify independently associated variants (MAF ≥ 1% and P < 5.56 × 10^−9^) (including ±500 kb of the associated loci). Miami plots were generated using the R program (version 3.4.4). Genetic correlations were calculated using GNOVA^87^ software by analyzing the summary statistics of the HapMap Phase 3 matched variants with MAF ≥ 5% (~869 K) based on allele frequencies from the 1000 Genomes Phase 3 East Asians. The genomic inflation factor was calculated with formula: λ = median(qchisq(1-P, 1))/qchisq(0.5,1) where P is a vector of *P*-values. The LD score regression intercept was estimated using LDSC(LD SCore) v1.01 with pre-calculated LD scores from 1000 Genome Project phase 3 East Asians by analyzing the summary statistics of the HapMap Phase 3 matched variants from meta-analysis results^37^. Candidate genes of each locus were listed by including the gene containing the lead variant or nearest genes of upstream and downstream of the lead variant. The lists were used as an input for GENE2FUNC analysis of FUMA-GWAS^40^. Tissue specificity was assessed by analyzing enrichment of differentially expressed gene sets in a certain tissue compared to all other tissue types using gene expression data sets of GTEx v8^40^. Classification performance of T2D by glycemic and erythrocytic components was assessed by AUC. To avoid overfitting, the mean AUC of a logistic regression model with GRS based on glycemic or erythrocytic components was estimated in a tenfold cross-validation framework from test sets.

### Reporting summary

Further information on research design is available in the Nature Research Reporting Summary linked to this article.

## Supplementary information

Supplementary Information

Description of Additional Supplementary Files

Supplementary Data 1-24

Reporting Summary

## Data Availability

Overall meta-analyses summary level results generated in this study are available at the Korea Biobank Array project website (http://koreanchip.org/kba130k/). The results include association results from the Korean population and meta-analysis combining the results of the Korean and the Japanese (BioBank Japan).
