# Supplementary material for: The contribution of common and rare genetic variants to variation in metabolic traits in 288,137 East Asians
Source: Nat Commun. 2022 Nov 4;13:6642. doi: 10.1038/s41467-022-34163-2 (PMC9636136; doi:10.1038/s41467-022-34163-2)

Supplementary Figure 1. Miami plot of common and rare associations for a glycemic trait (FPG)

Miami plot shows linear regression analysis results of common variants (upper panel) and rare variants (lower panel). Red horizontal line indicates -log10(5.56e-9) and -log10(7.61e-8) for upper and lower panels, respectively. Previously known loci were colored in blue for ±250kb of the lead signal and colored in red for ±250kb of new associations of this study

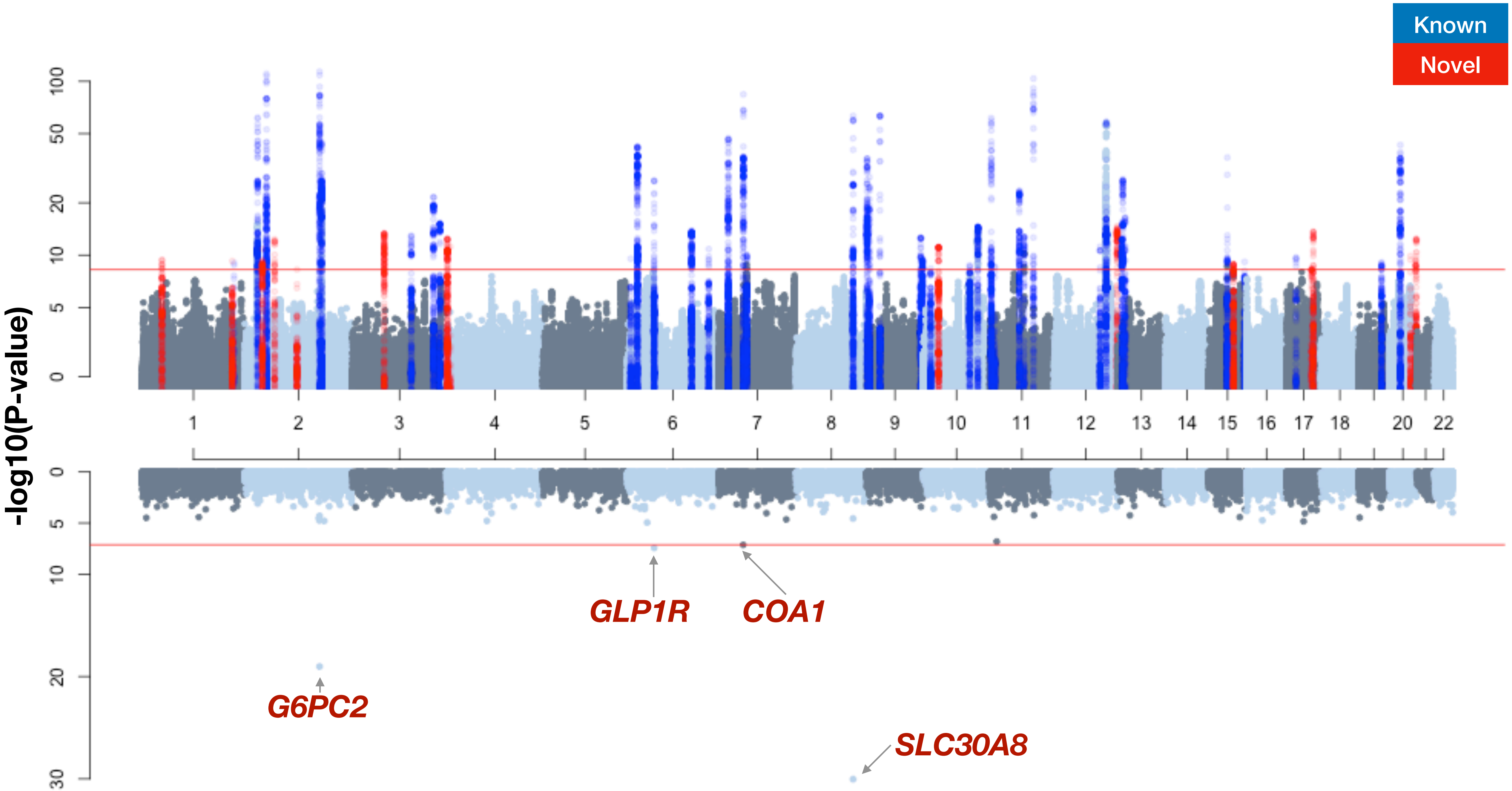

Supplementary Figure 2. Miami plot of common and rare associations for lipid traits

Miami plot shows linear regression analysis results of common variants (upper panel) and rare variants (lower panel). Red horizontal line indicates -log10(5.56e-9) and -log10(7.61e-8) for upper and lower panels, respectively. Previously known loci were colored in blue for ±250kb of the lead signal and colored in red for ±250kb of new associations of this study

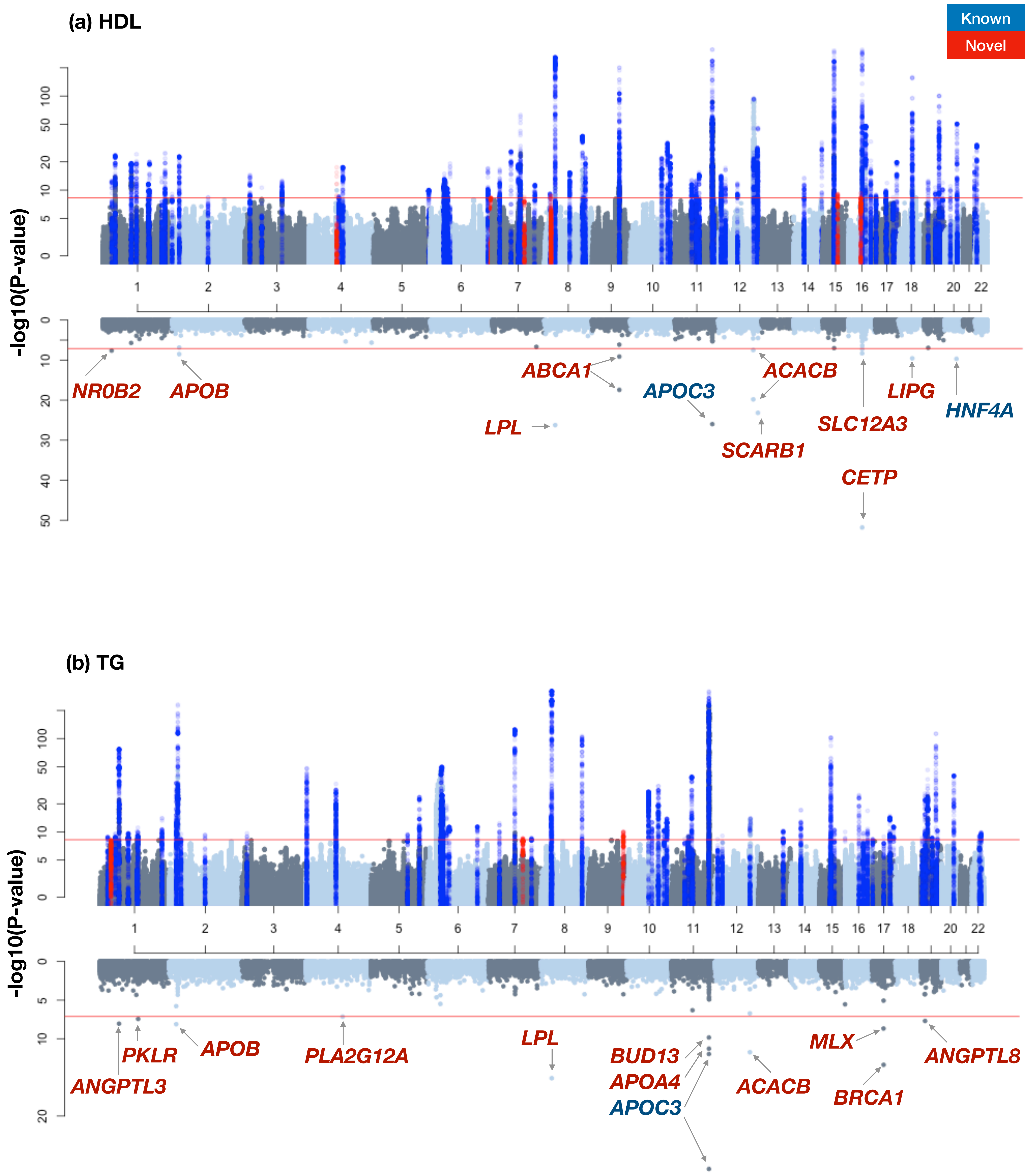

Supplementary Figure 2. Miami plot of common and rare associations for lipid traits

Miami plot shows linear regression analysis results of common variants (upper panel) and rare variants (lower panel). Red horizontal line indicates -log10(5.56e-9) and -log10(7.61e-8) for upper and lower panels, respectively. Previously known loci were colored in blue for ±250kb of the lead signal and colored in red for ±250kb of new associations of this study

(c) TC

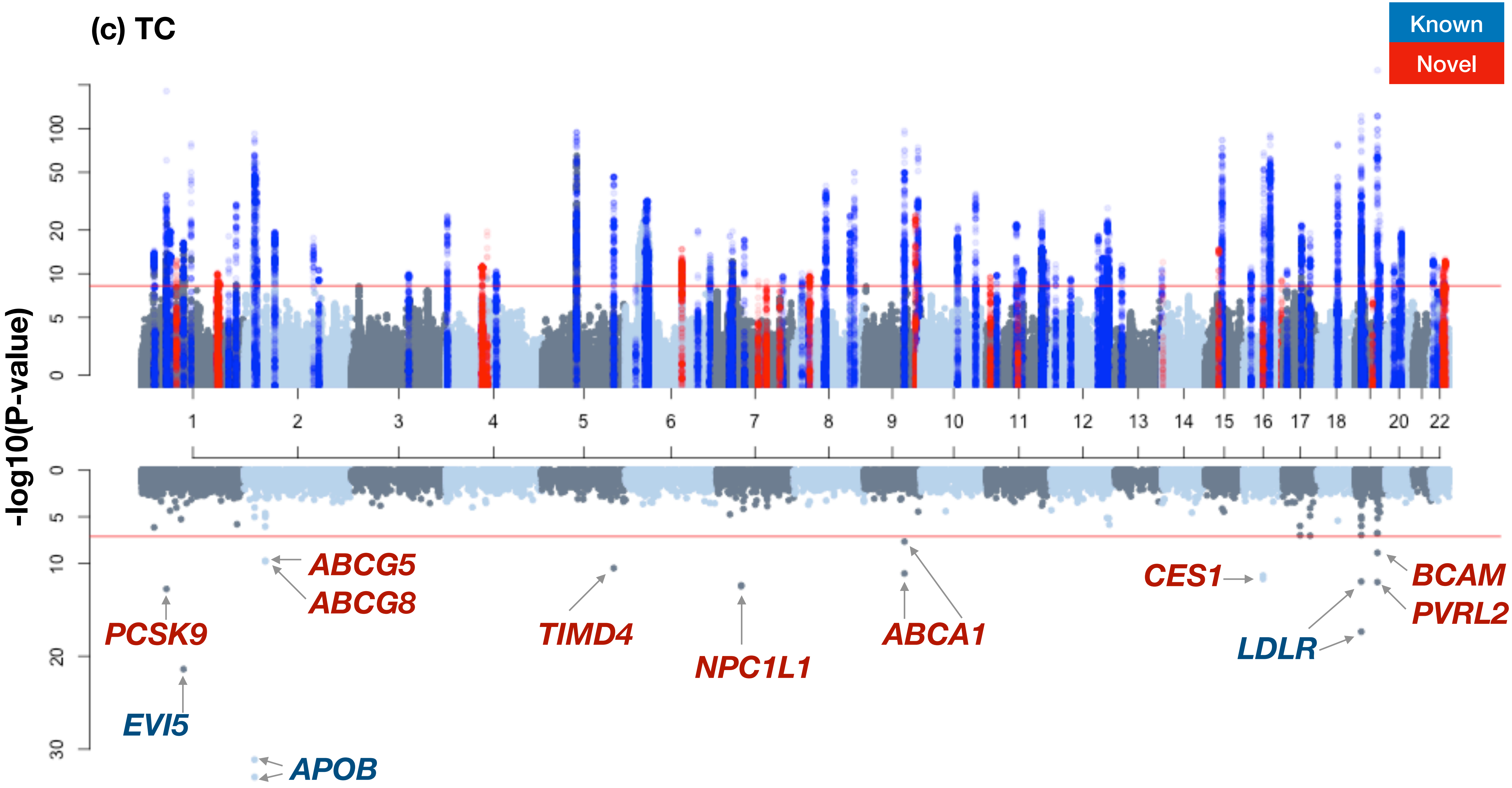

Supplementary Figure 3. Miami plot of common and rare associations for liver enzymes

Miami plot shows linear regression analysis results of common variants (upper panel) and rare variants (lower panel). Red horizontal line indicates -log10(5.56e-9) and -log10(7.61e-8) for upper and lower panels, respectively. Previously known loci were colored in blue for ±250kb of the lead signal and colored in red for ±250kb of new associations of this study

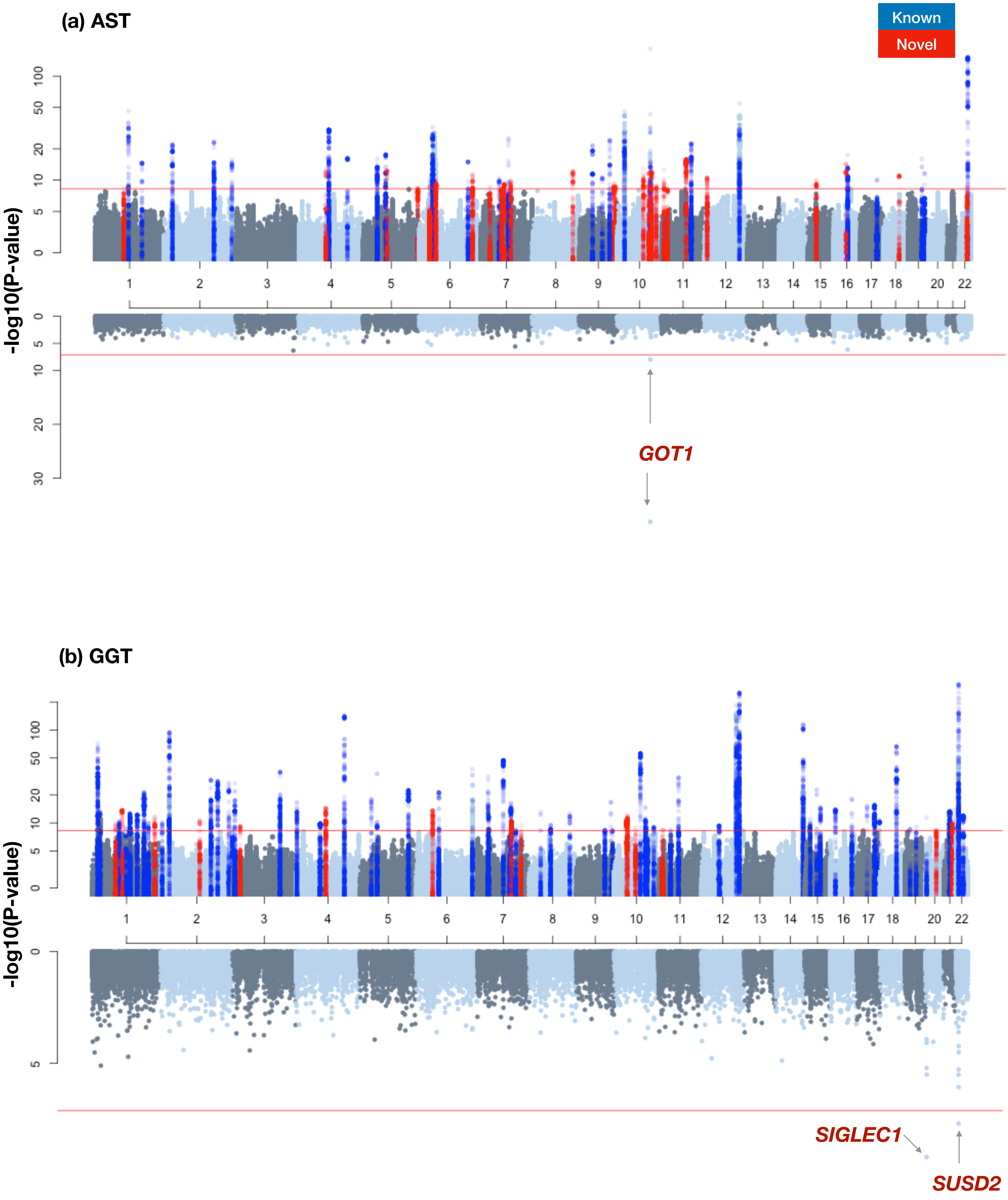

Supplementary Figure 4. Tissue specificity of known and novel loci

(a) ALT

Significantly enriched DEG(Differentially Expressed Gene) sets (Bonferroni corrected P < 0.05) are highlighted in red. Using GENE2FUNC of FUMA-GWAS, pre-calculated DEG sets were compared with input genes (candidate genes from known or novel loci) using the hypergeometric test

Known loci

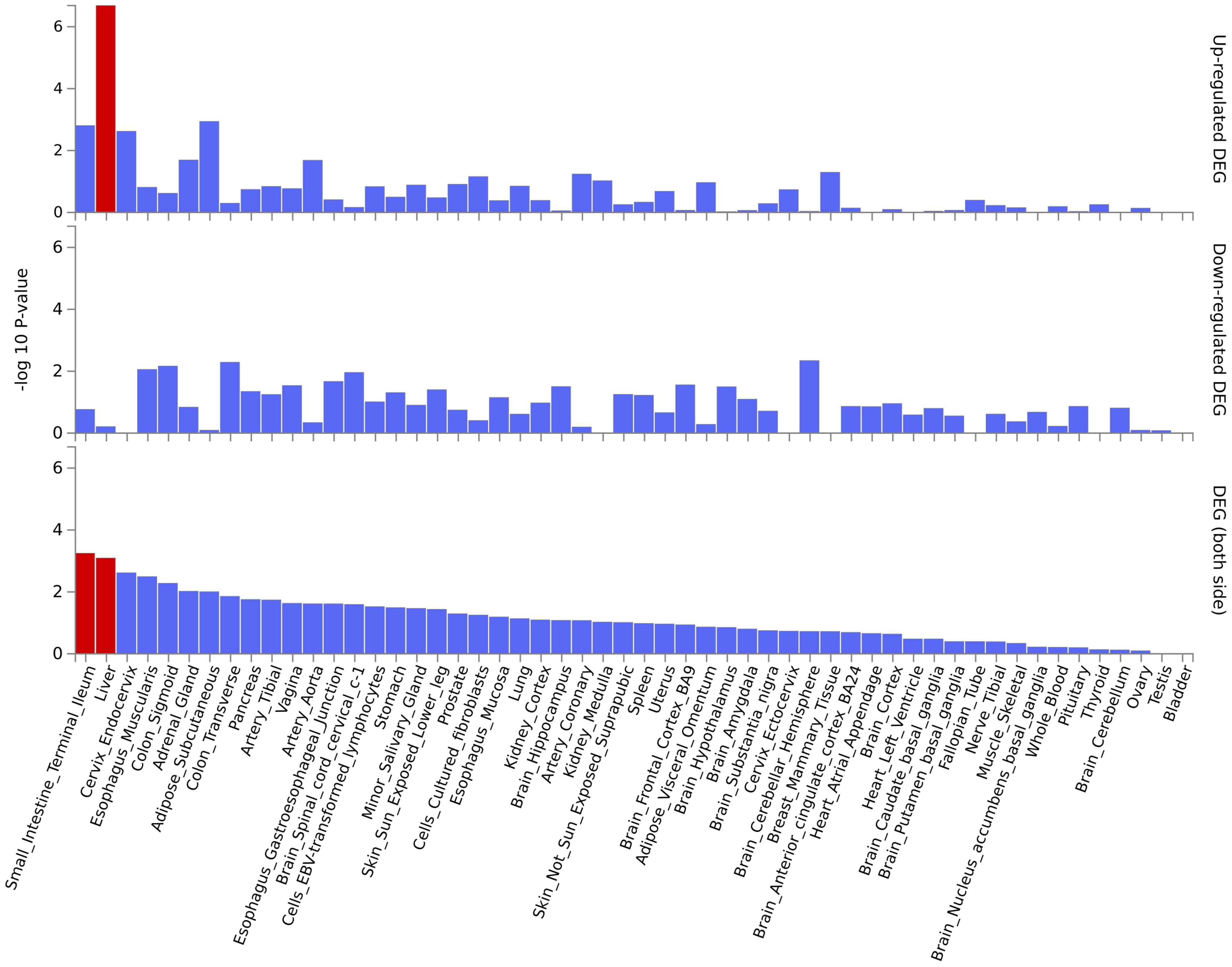

Novel loci

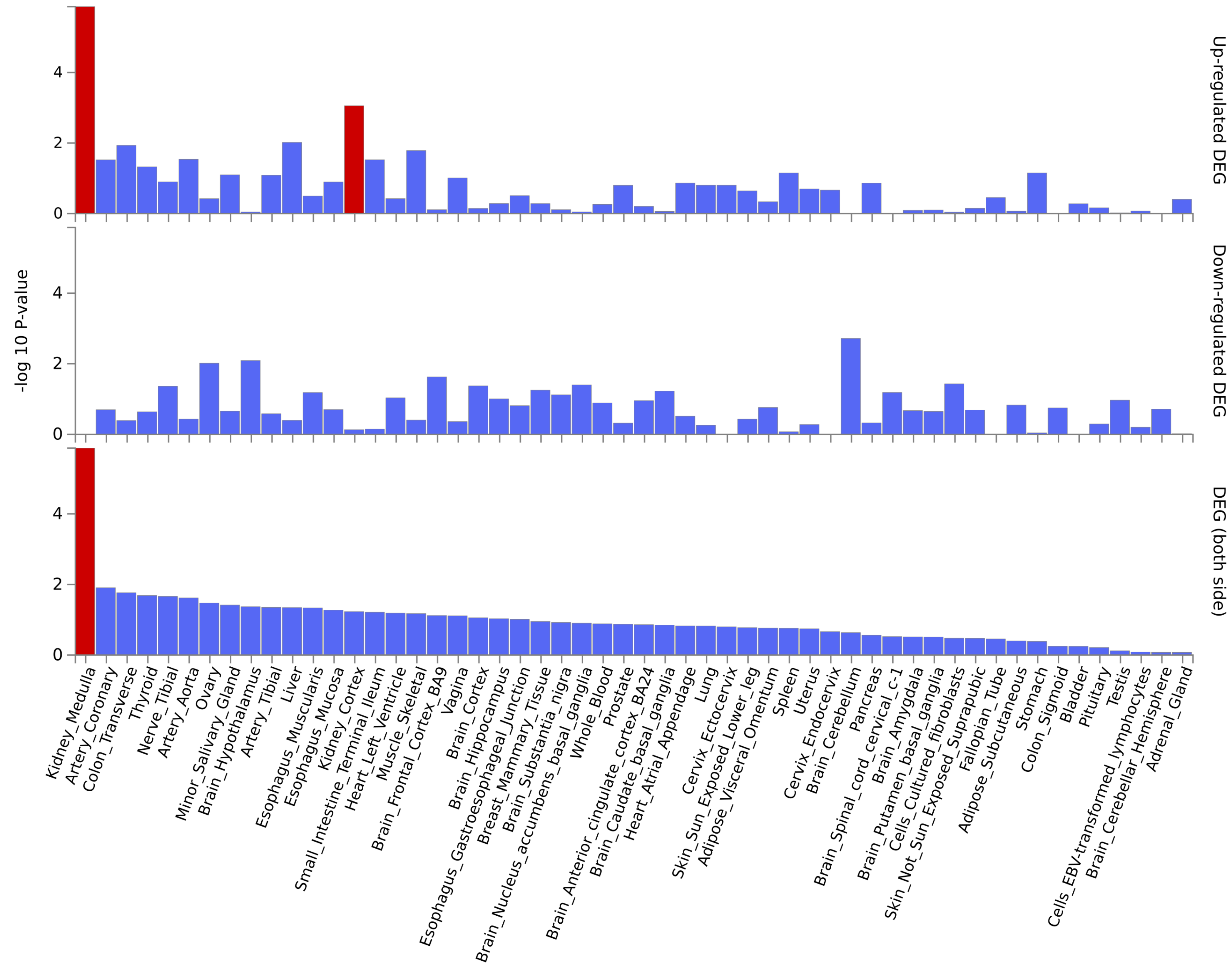

Supplementary Figure 4. Tissue specificity of known and novel loci

(b) AST Significantly enriched DEG(Differentially Expressed Gene) sets (Bonferroni corrected P < 0.05) are highlighted in red. Using GENE2FUNC of FUMA-GWAS, pre-calculated DEG sets were compared with input genes (candidate genes from known or novel loci) using the hypergeometric test

Known loci

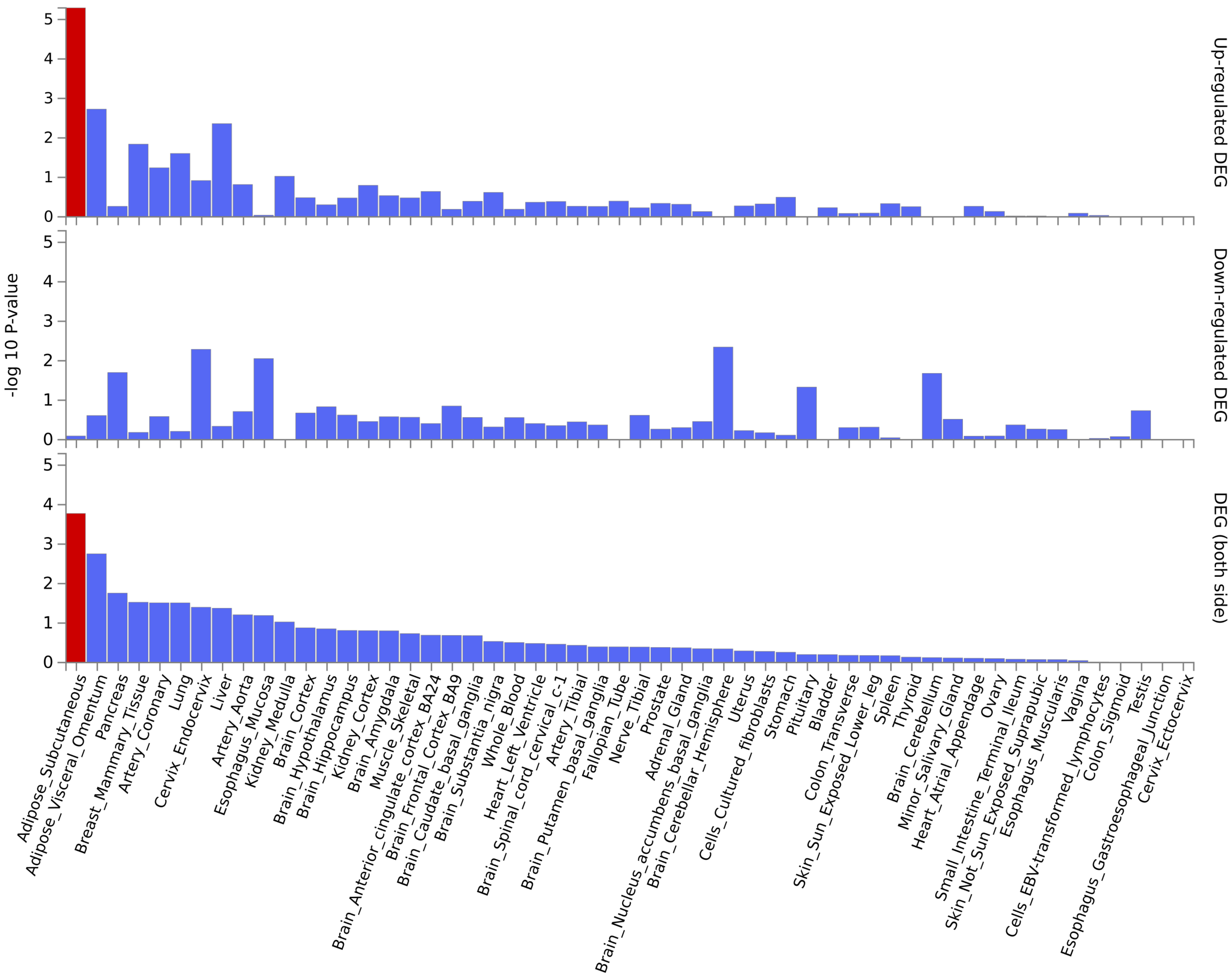

Novel loci

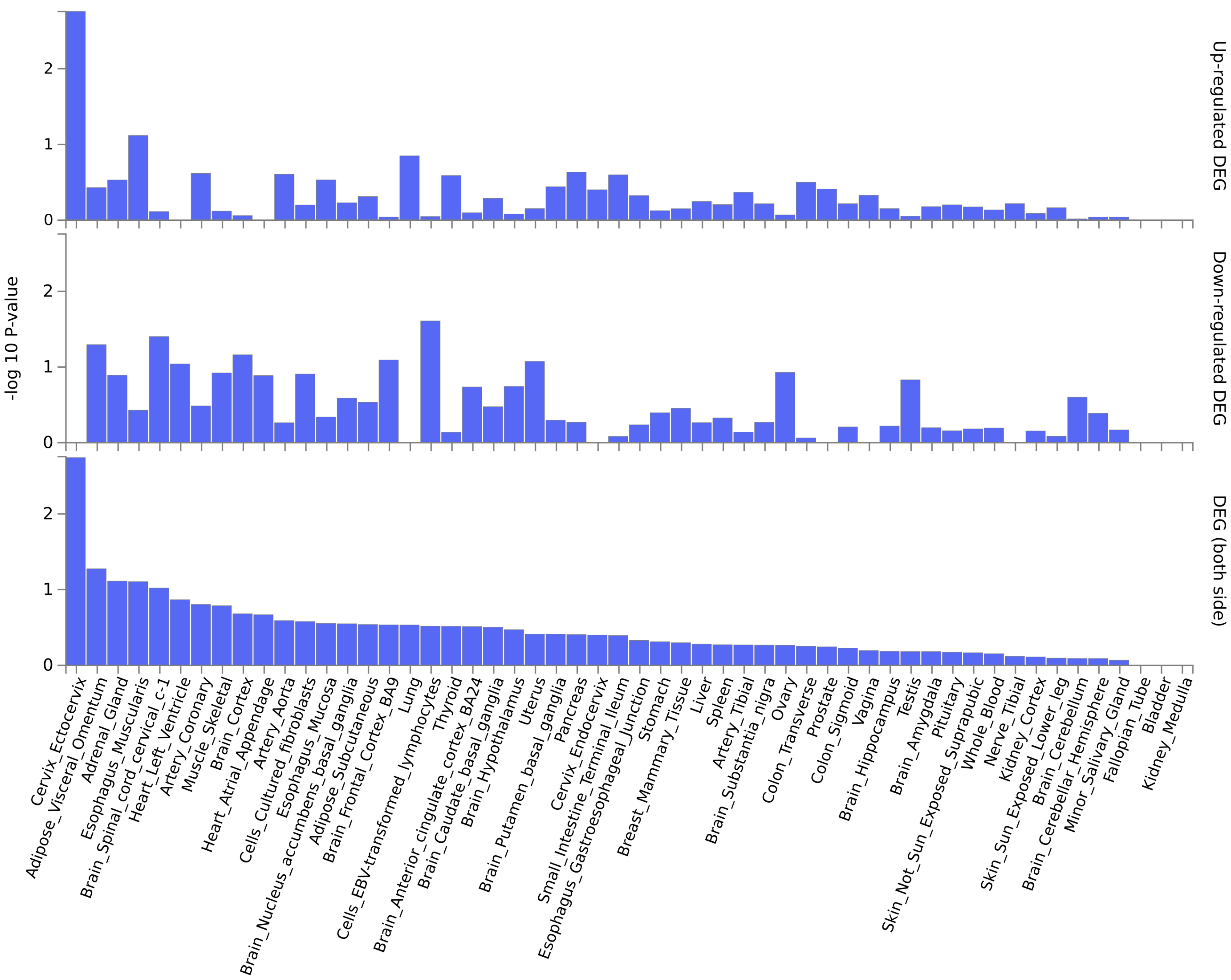

Supplementary Figure 4. Tissue specificity of known and novel loci

(c) GGT

Significantly enriched DEG(Differentially Expressed Gene) sets (Bonferroni corrected P < 0.05) are highlighted in red. Using GENE2FUNC of FUMA-GWAS, pre-calculated DEG sets were compared with input genes (candidate genes from known or novel loci) using the hypergeometric test

Known loci

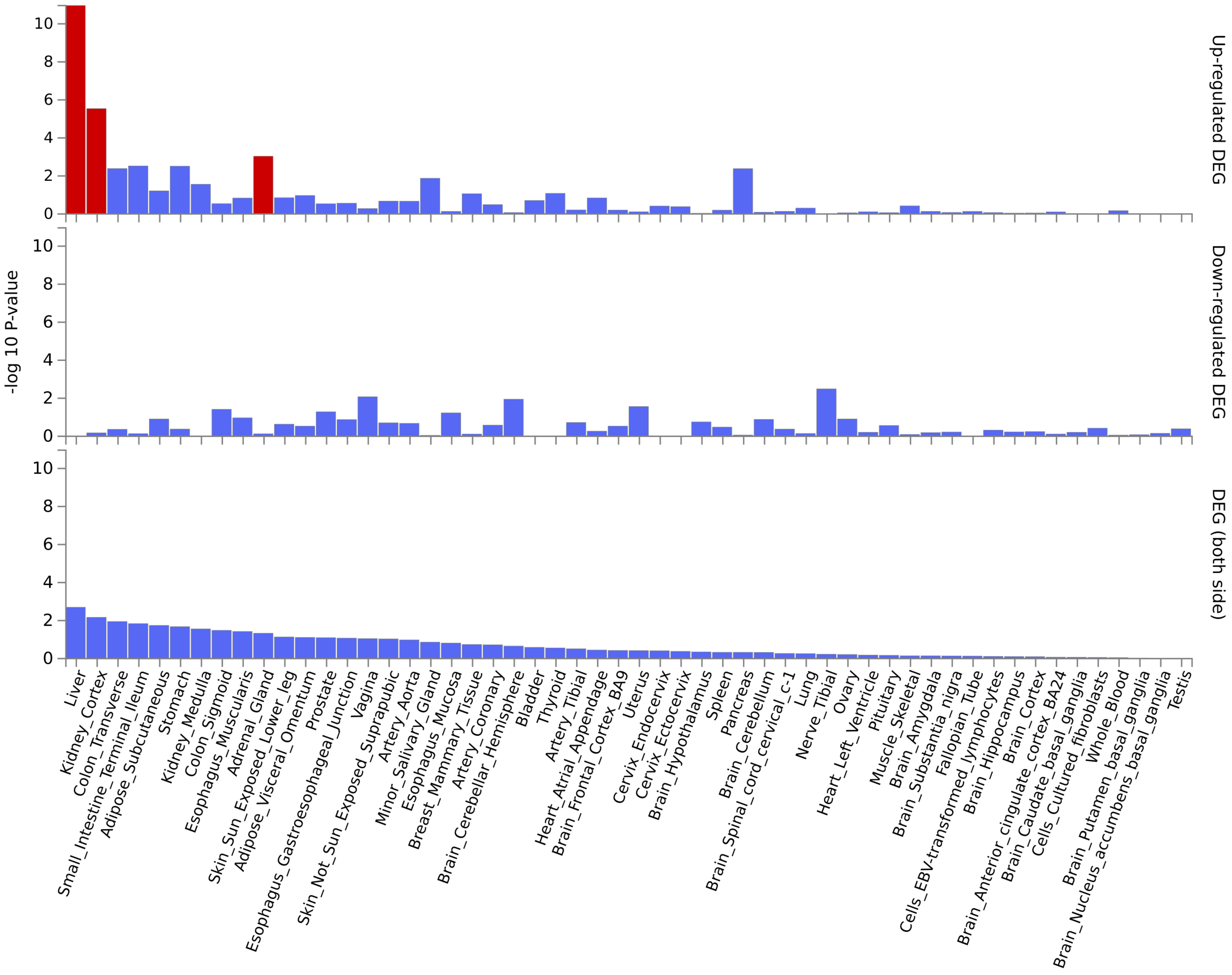

Novel loci

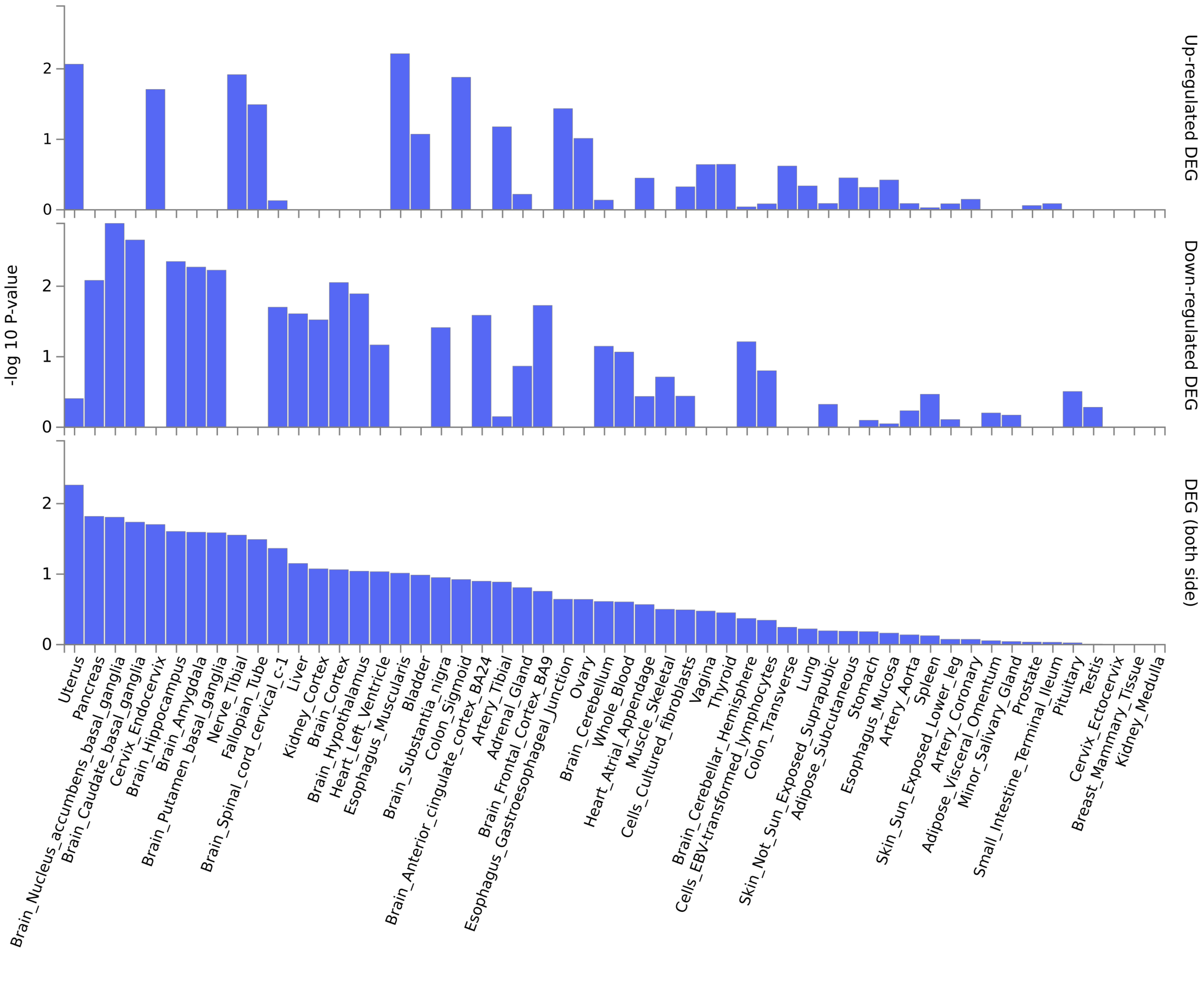

Supplementary Figure 4. Tissue specificity of known and novel loci

(d) FPG

Significantly enriched DEG(Differentially Expressed Gene) sets (Bonferroni corrected P < 0.05) are highlighted in red. Using GENE2FUNC of FUMA-GWAS, pre-calculated DEG sets were compared with input genes (candidate genes from known or novel loci) using the hypergeometric test

Known loci

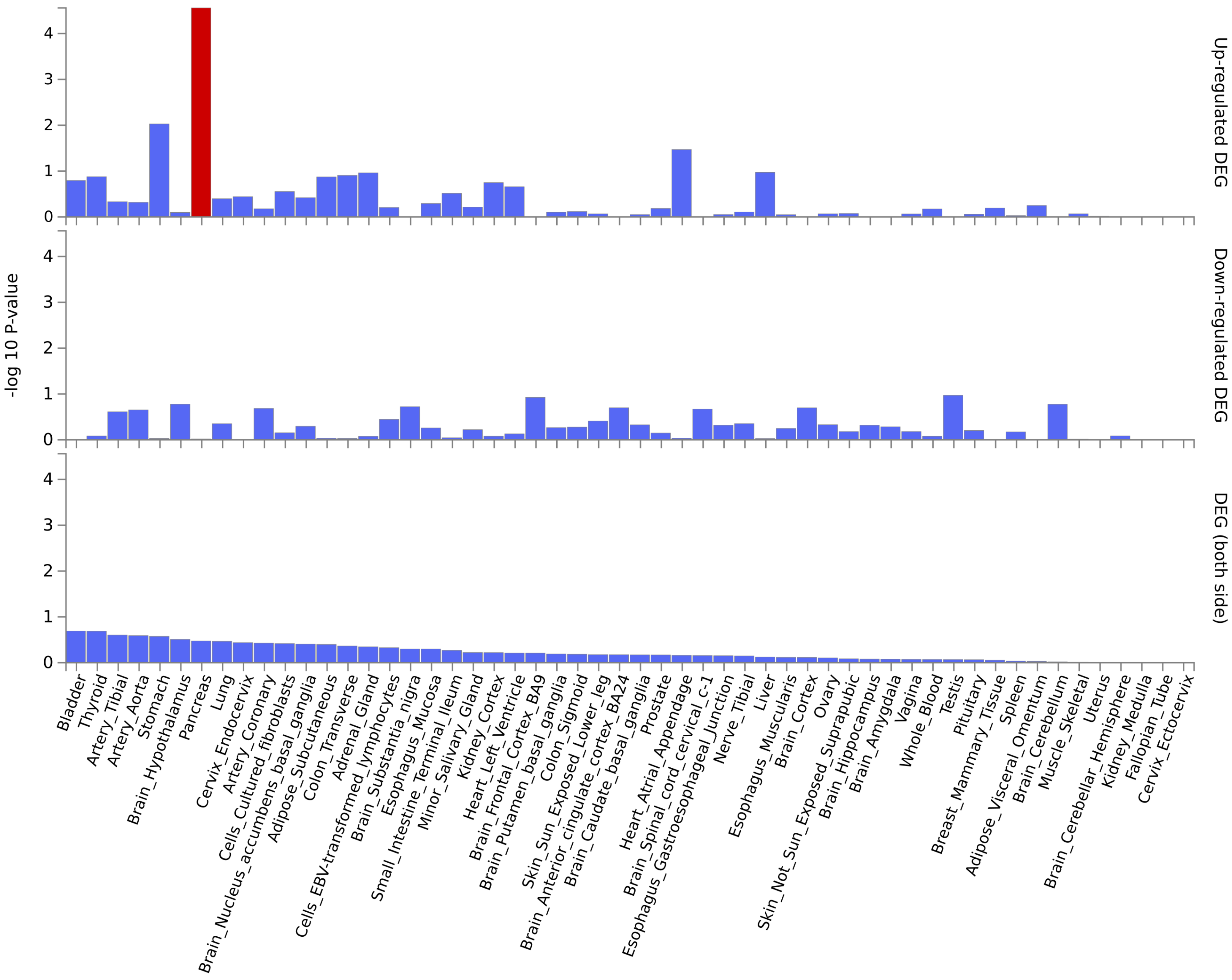

Novel loci

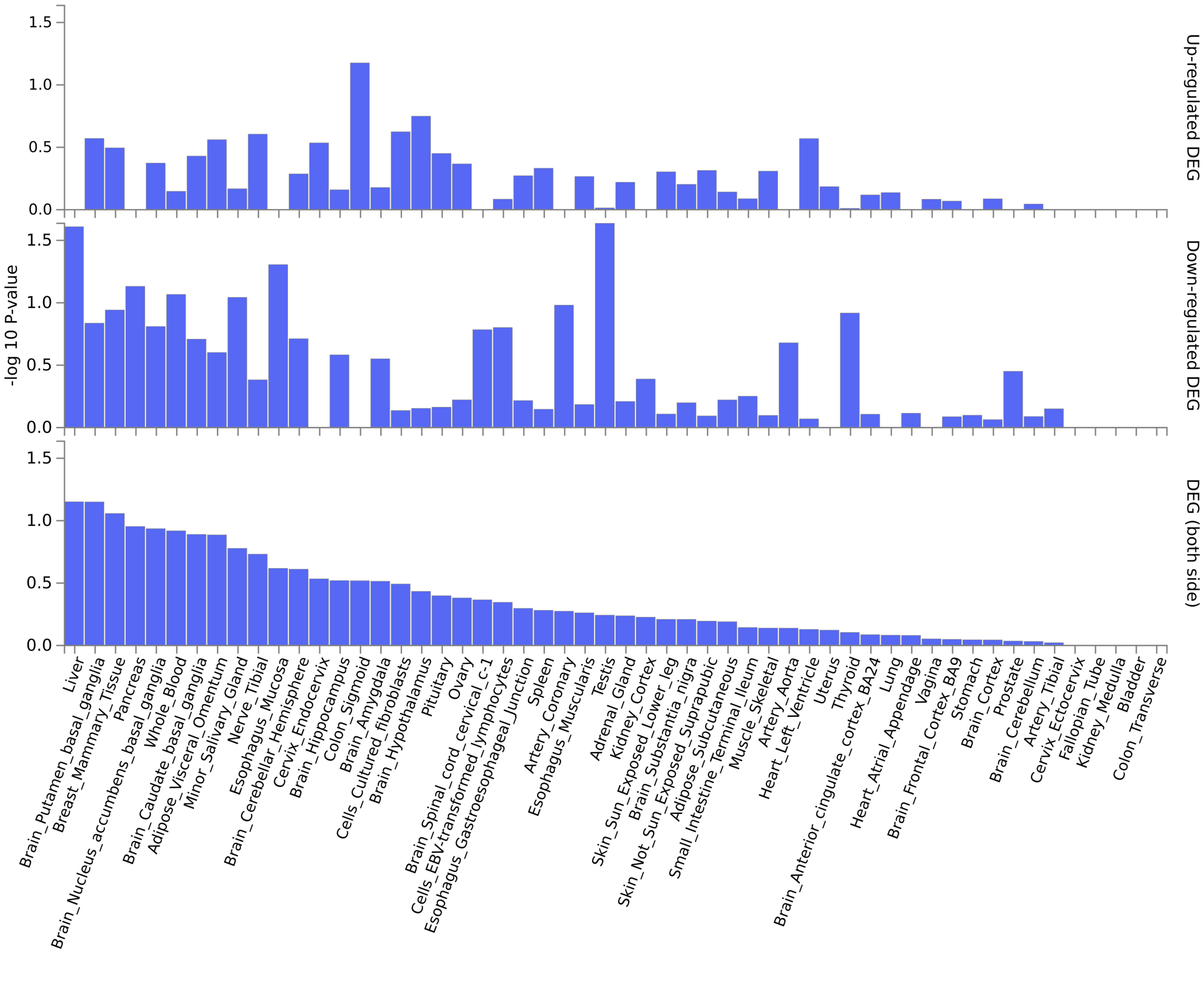

Supplementary Figure 4. Tissue specificity of known and novel loci

(e) HbA1c Significantly enriched DEG(Differentially Expressed Gene) sets (Bonferroni corrected P < 0.05) are highlighted in red. Using GENE2FUNC of FUMA-GWAS, pre-calculated DEG sets were compared with input genes (candidate genes from known or novel loci) using the hypergeometric test

Known loci

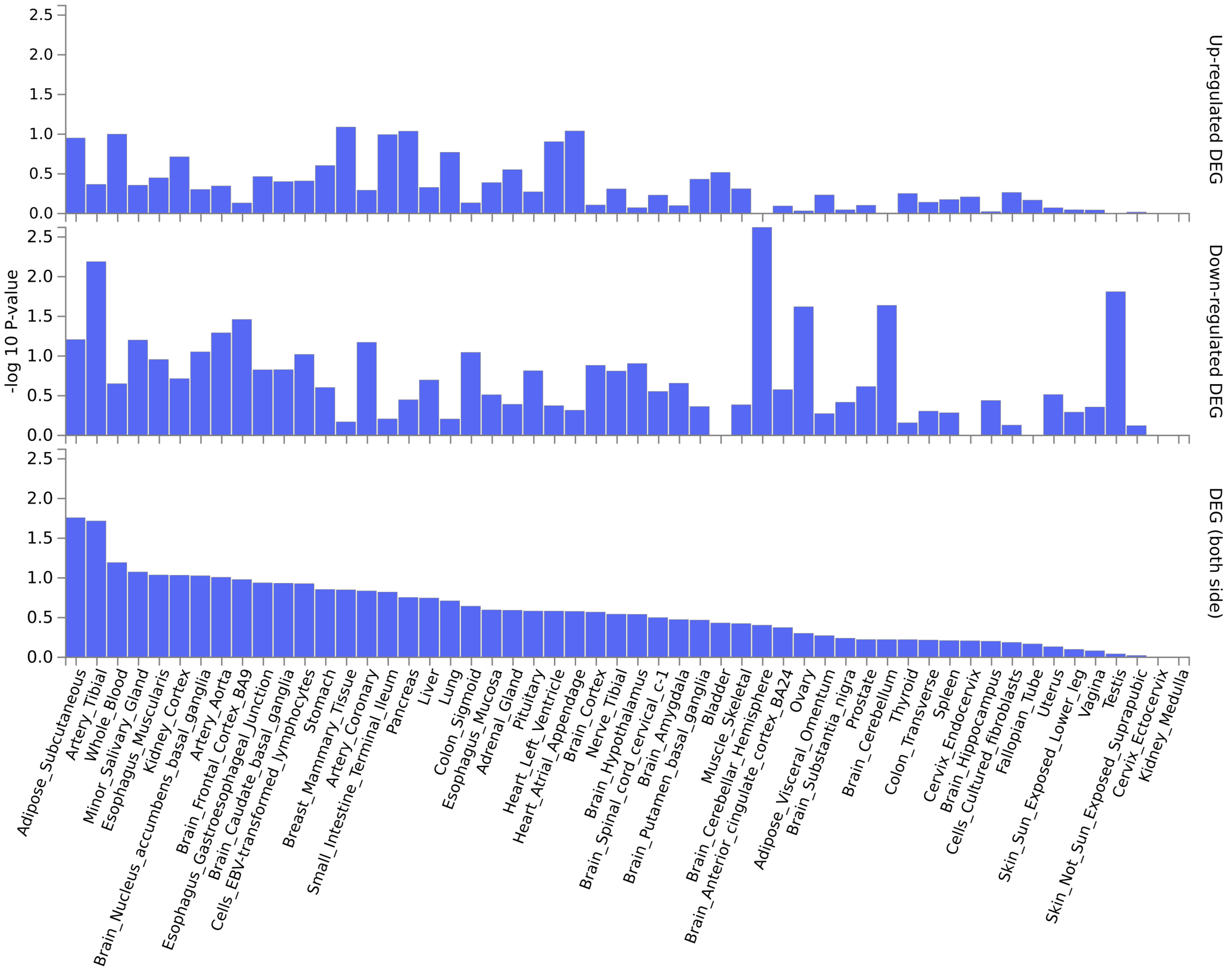

Novel loci

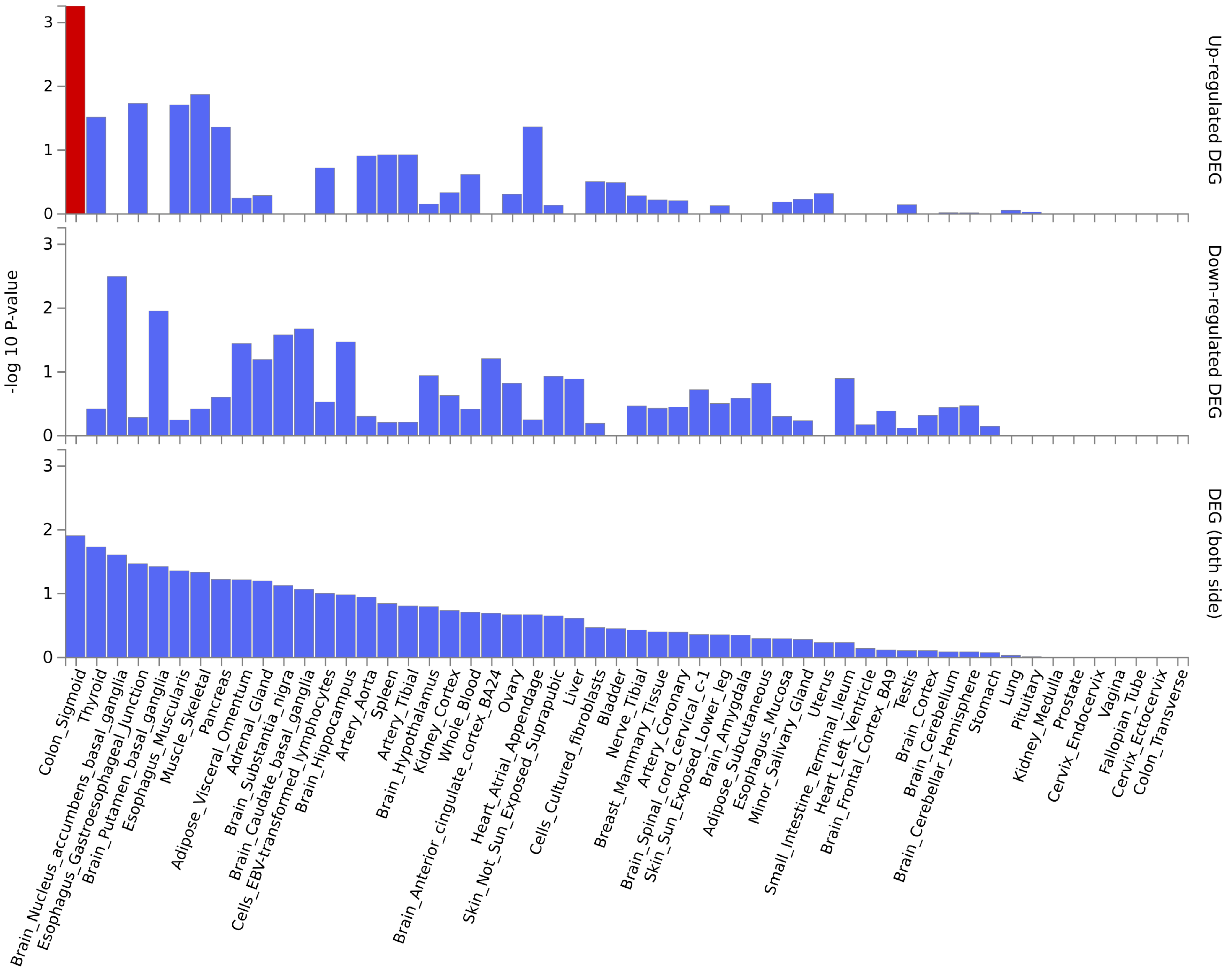

Supplementary Figure 4. Tissue specificity of known and novel loci

(f) HDL

Significantly enriched DEG(Differentially Expressed Gene) sets (Bonferroni corrected P < 0.05) are highlighted in red. Using GENE2FUNC of FUMA-GWAS, pre-calculated DEG sets were compared with input genes (candidate genes from known or novel loci) using the hypergeometric test

Known loci

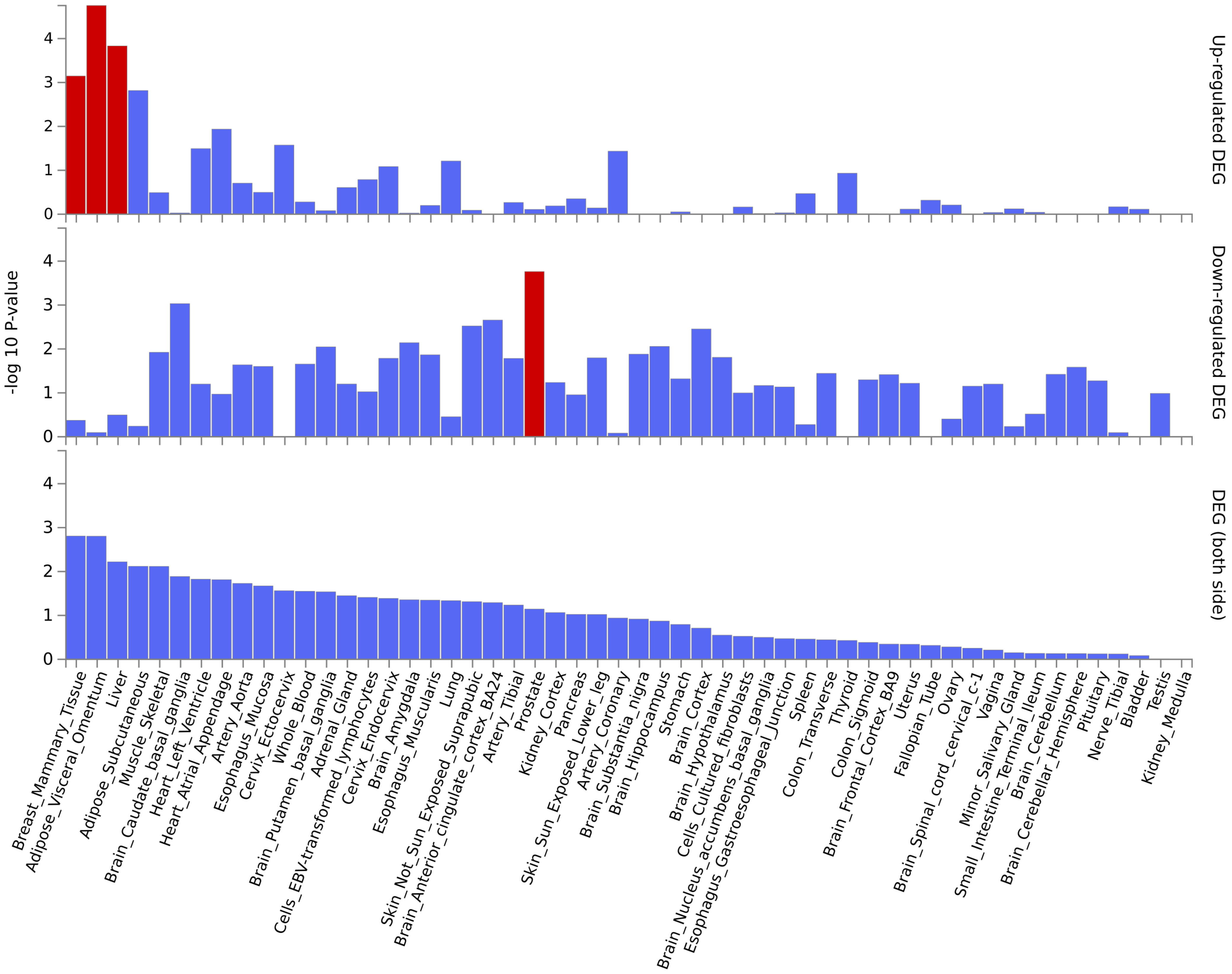

Novel loci

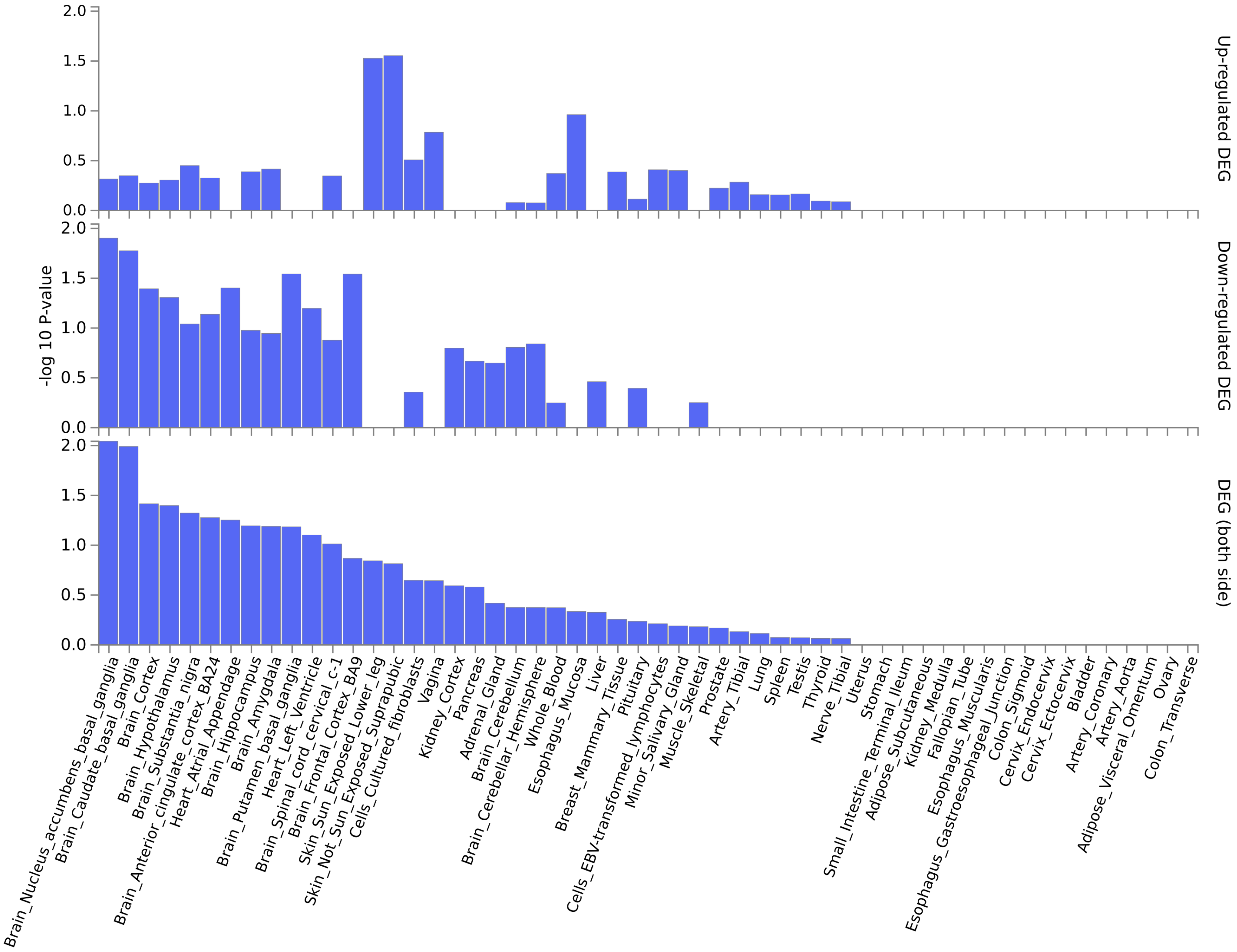

Supplementary Figure 4. Tissue specificity of known and novel loci

(g) LDL Significantly enriched DEG(Differentially Expressed Gene) sets (Bonferroni corrected P < 0.05) are highlighted in red. Using GENE2FUNC of FUMA-GWAS, pre-calculated DEG sets were compared with input genes (candidate genes from known or novel loci) using the hypergeometric test

Known loci

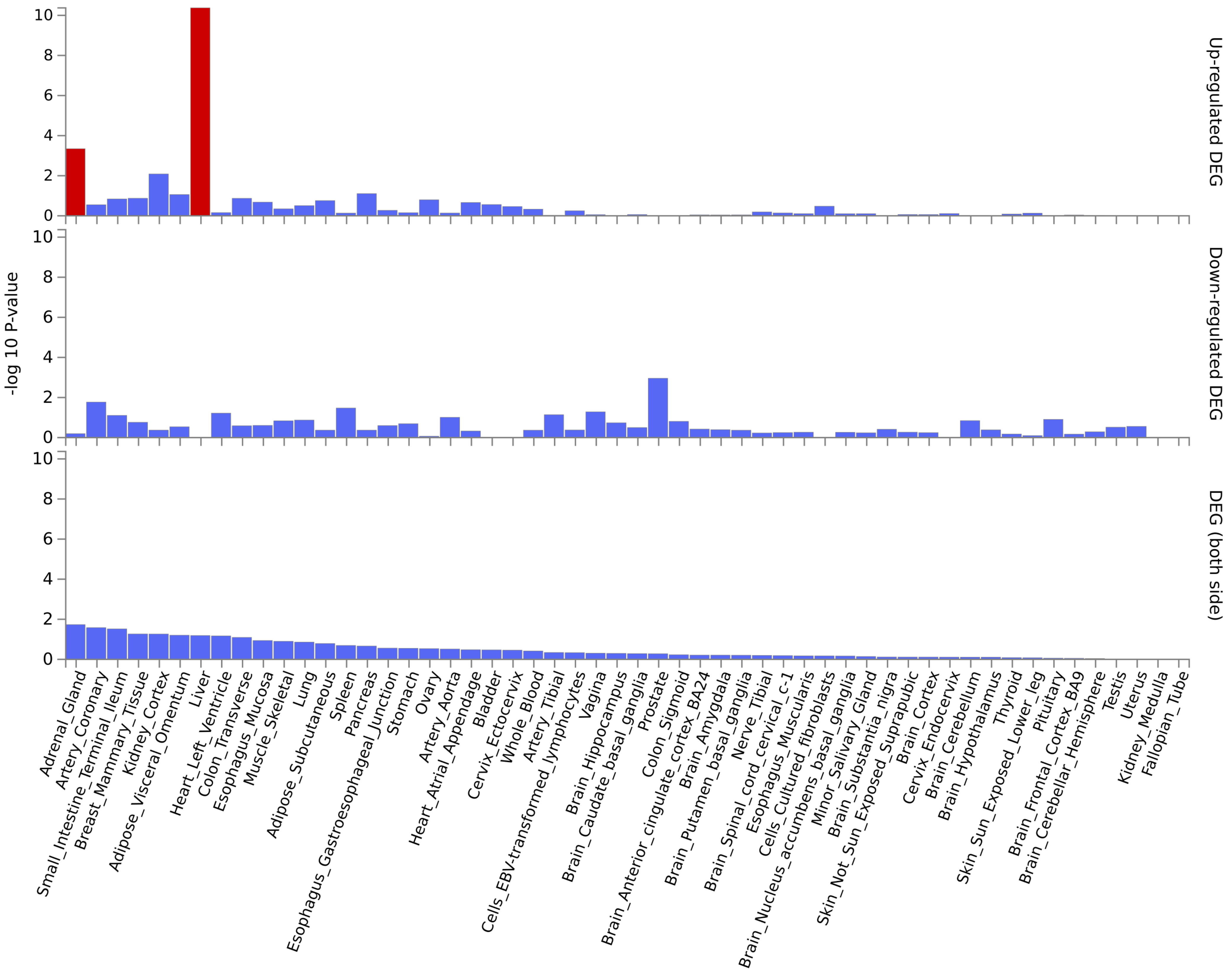

Novel loci

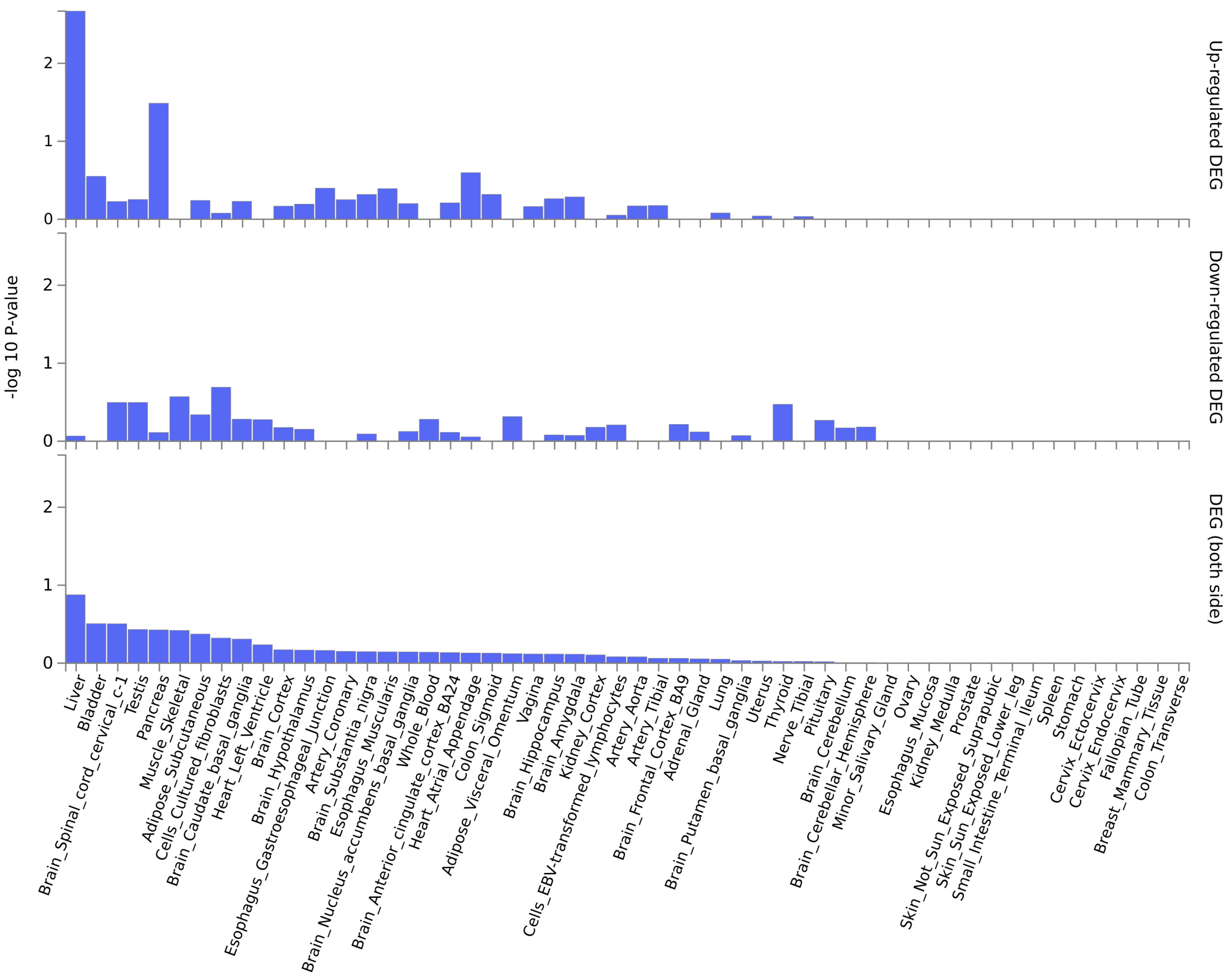

Supplementary Figure 4. Tissue specificity of known and novel loci

(h) TG

Significantly enriched DEG(Differentially Expressed Gene) sets (Bonferroni corrected P < 0.05) are highlighted in red. Using GENE2FUNC of FUMA-GWAS, pre-calculated DEG sets were compared with input genes (candidate genes from known or novel loci) using the hypergeometric test

Known loci

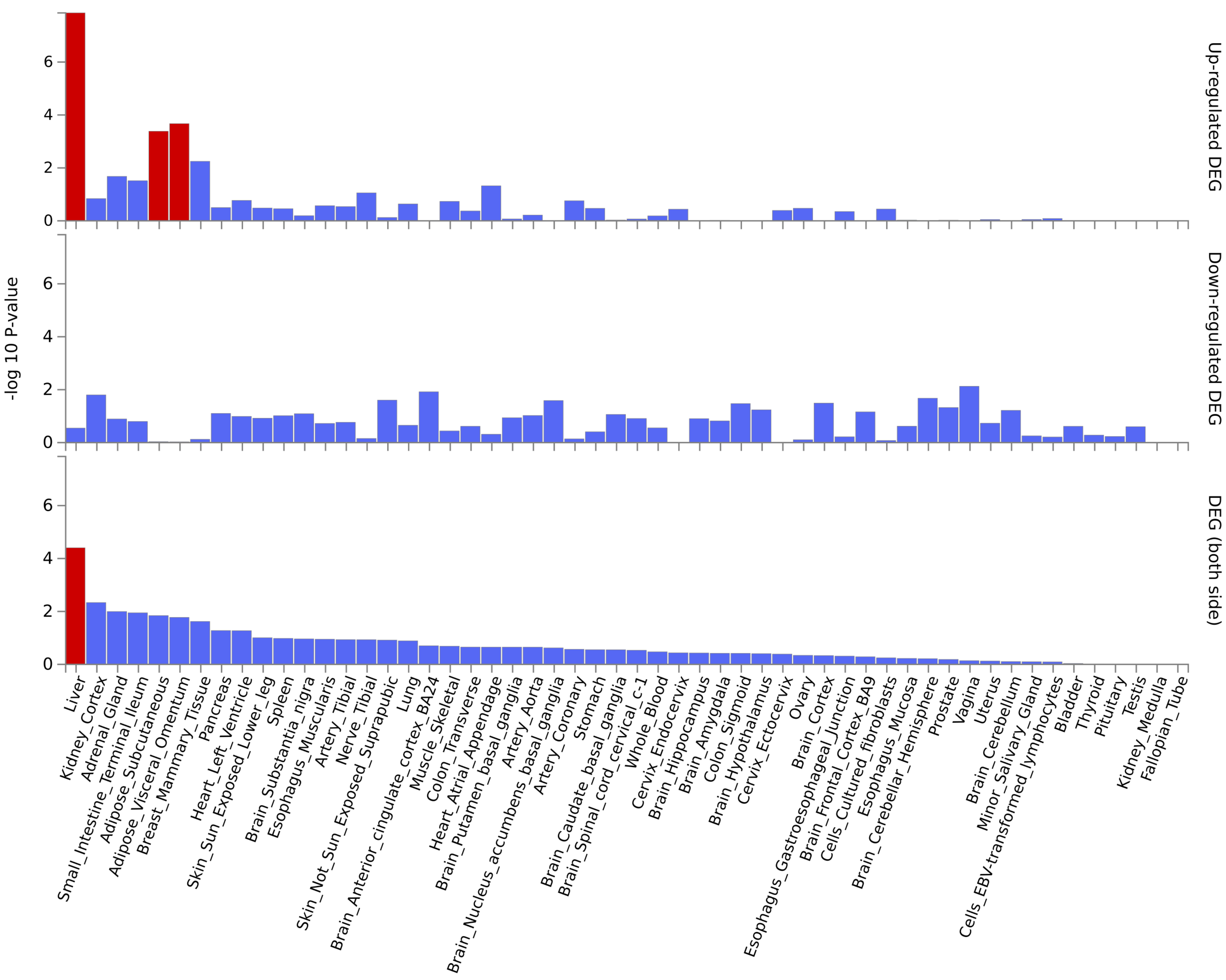

Novel loci

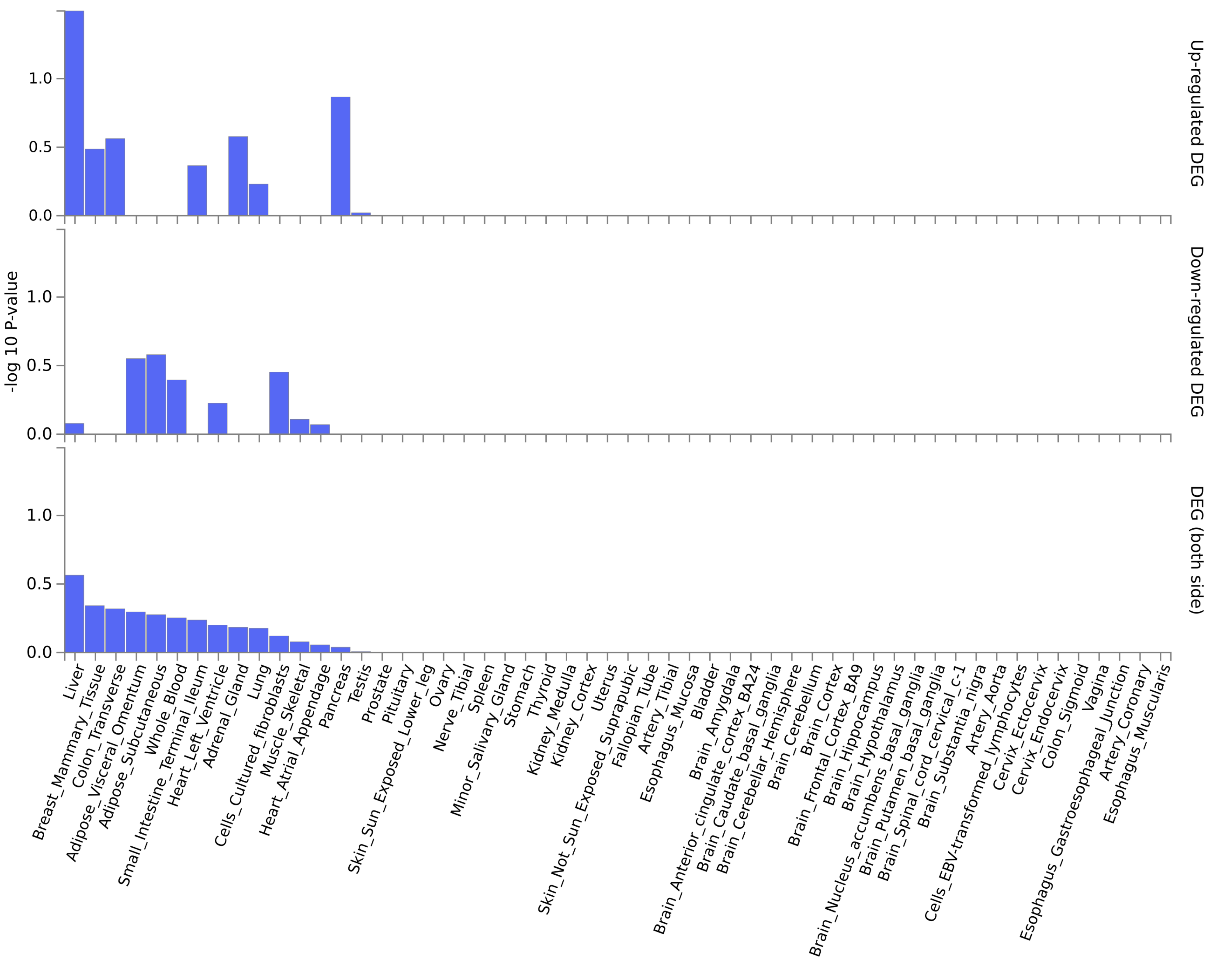

Supplementary Figure 4. Tissue specificity of known and novel loci

(i) TC

Significantly enriched DEG(Differentially Expressed Gene) sets (Bonferroni corrected P < 0.05) are highlighted in red. Using GENE2FUNC of FUMA-GWAS, pre-calculated DEG sets were compared with input genes (candidate genes from known or novel loci) using the hypergeometric test

Known loci

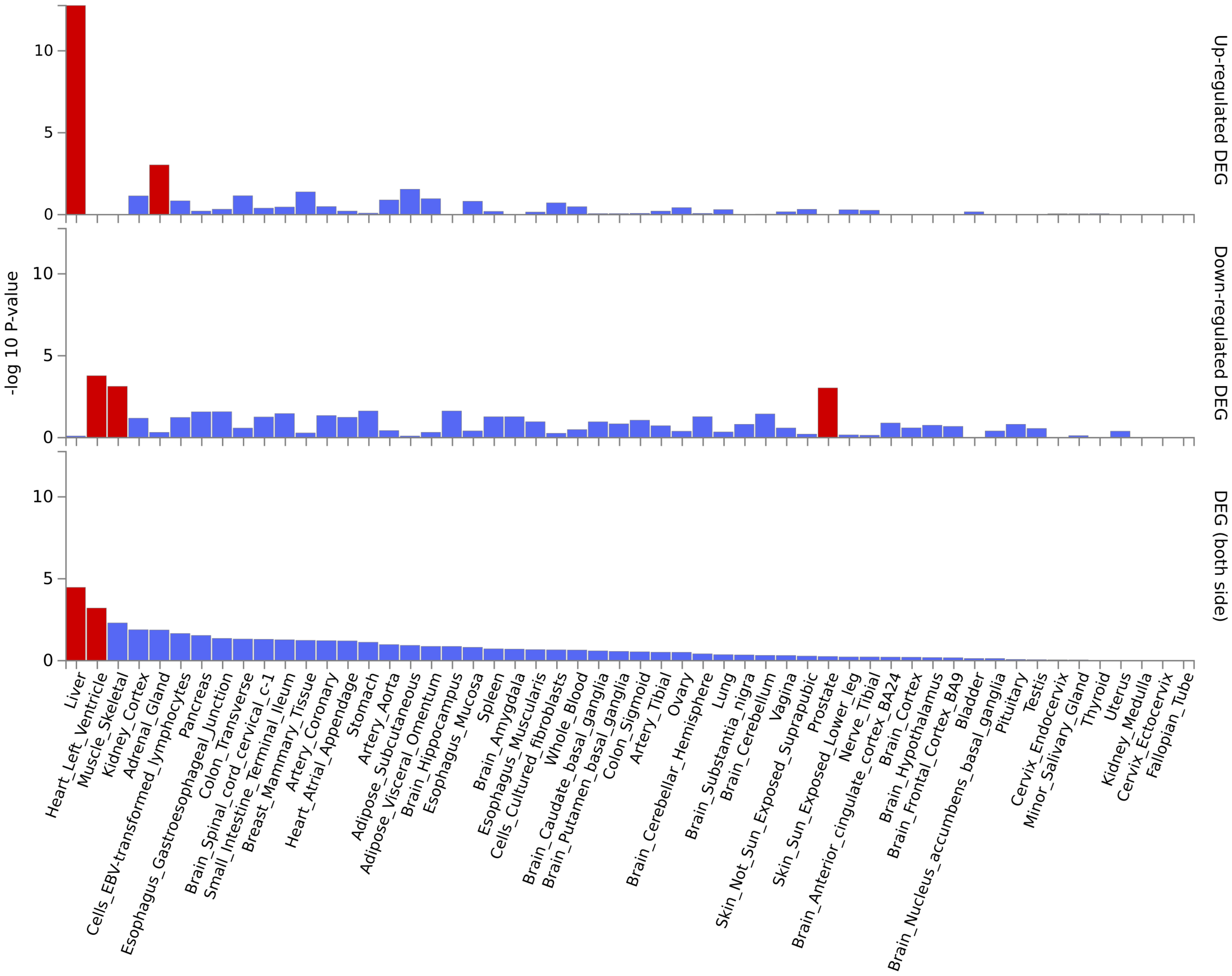

Novel loci

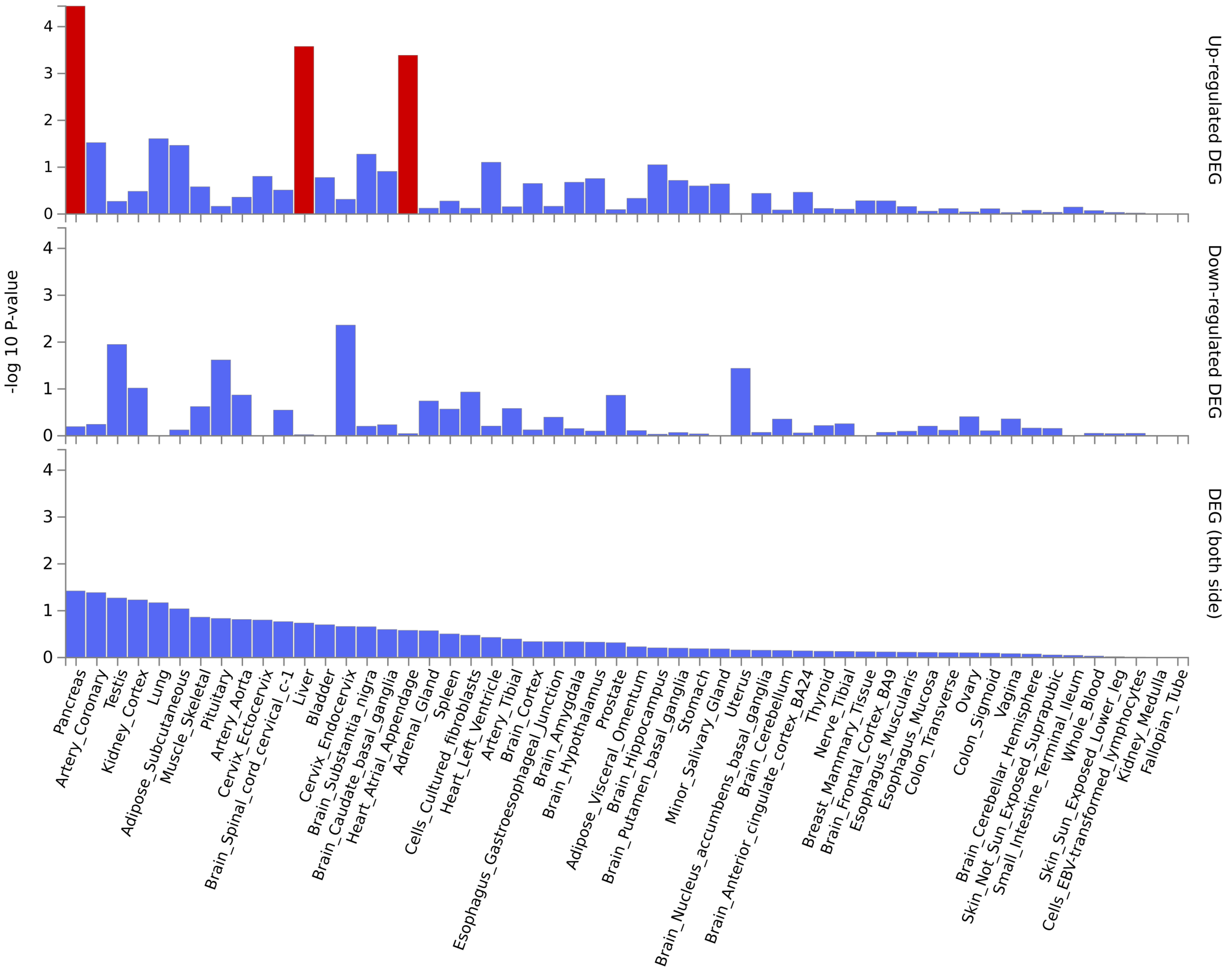

Supplementary Figure 5. Comparison of genetic effects of common lead variants between KBA and replication study (UK Biobank)

The points are all the common lead variants identified in the discovered study.
Effect sizes were compared based on the effect allele of this study. Error bars represent standard errors of effect sizes. 'n' indicates # of variants.

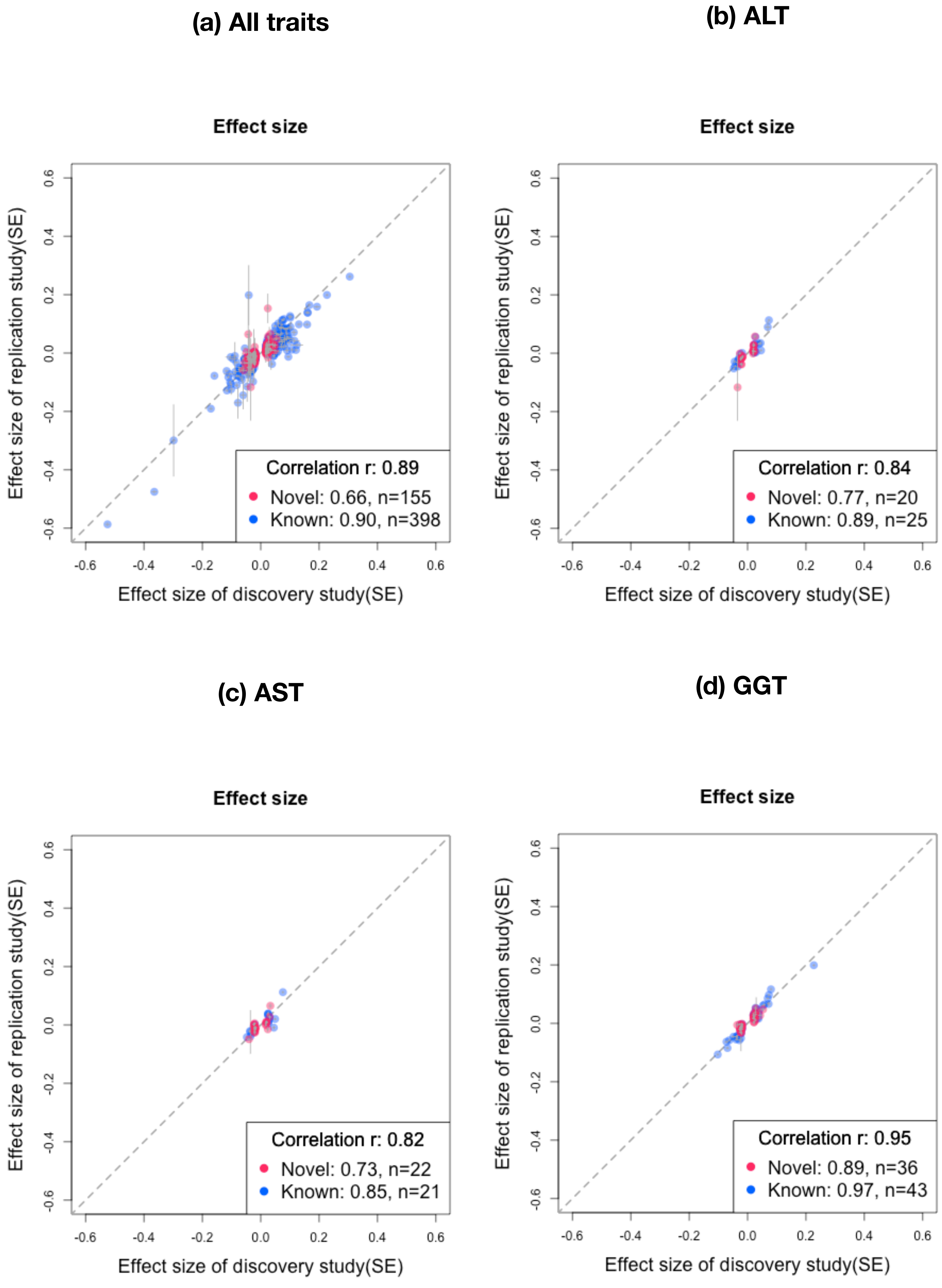

Supplementary Figure 5. Comparison of genetic effects of common lead variants between KBA and replication study (UK Biobank)

The points are all the common lead variants identified in the discovered study.
Effect sizes were compared based on the effect allele of this study. Error bars represent standard errors of effect sizes. 'n' indicates # of variants.

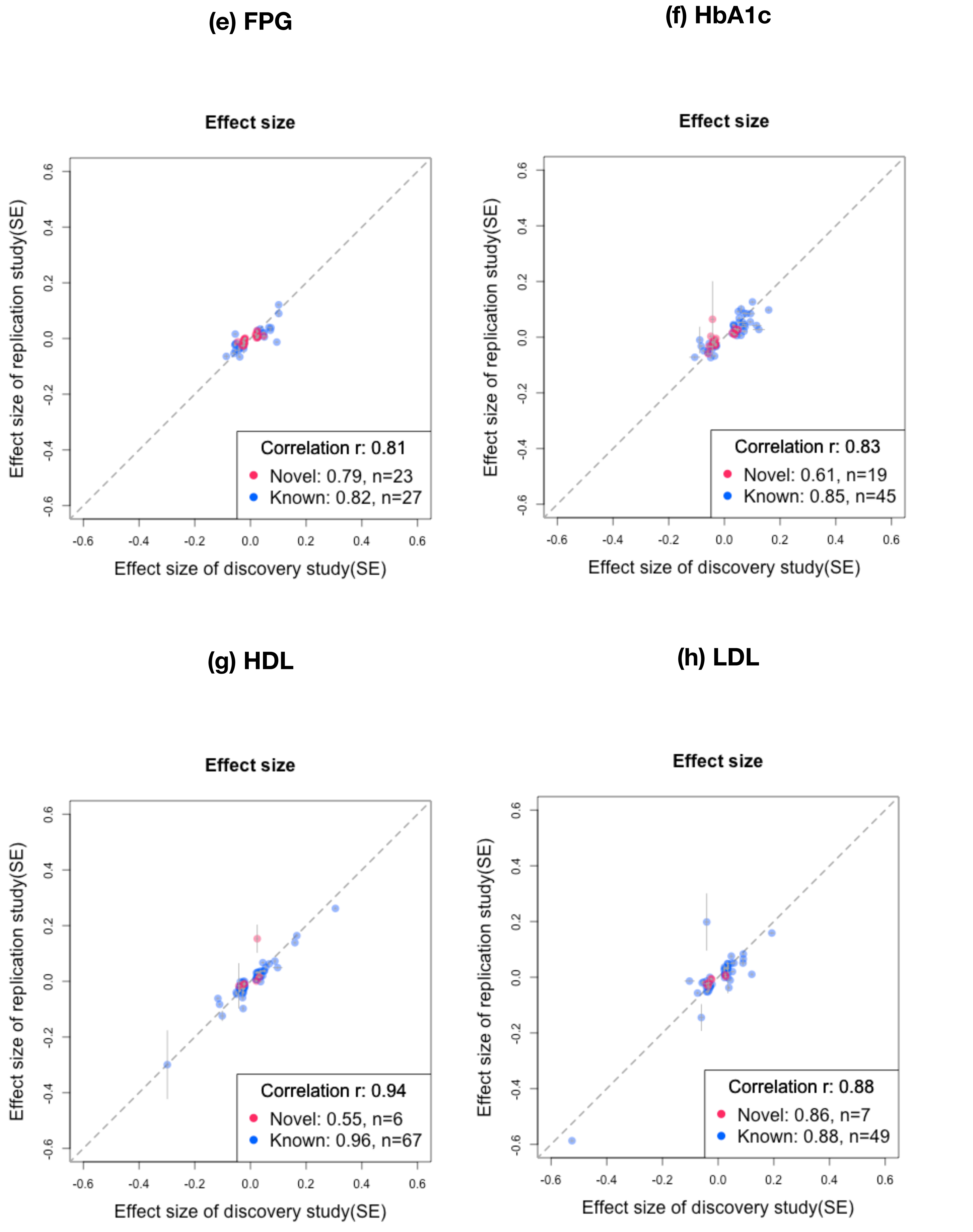

Supplementary Figure 5. Comparison of genetic effects of common lead variants between KBA and replication study (UK Biobank)

The points are all the common lead variants identified in the discovered study.
Effect sizes were compared based on the effect allele of this study. Error bars represent standard errors of effect sizes. 'n' indicates # of variants.

(i) TC

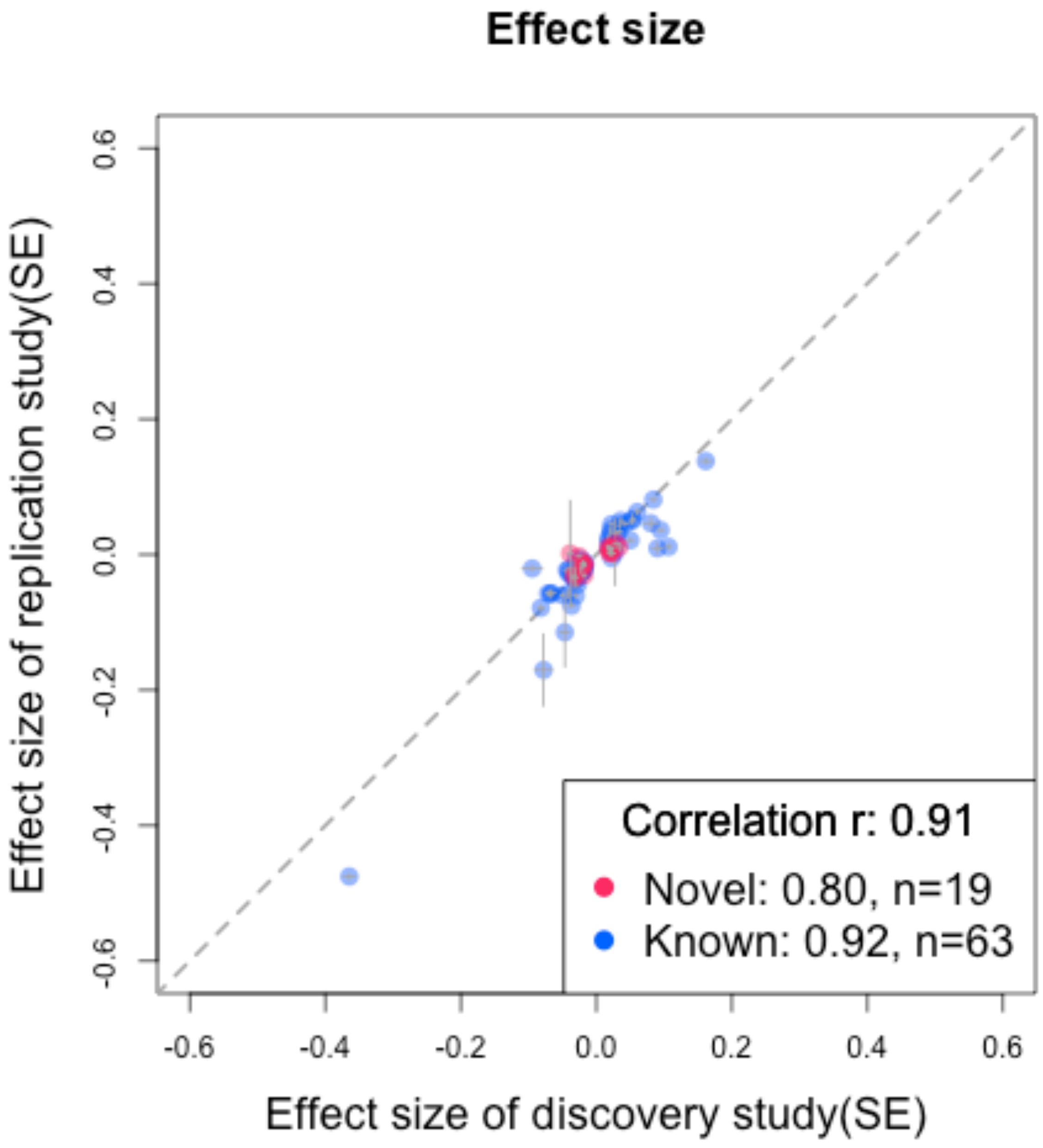

(j) TG

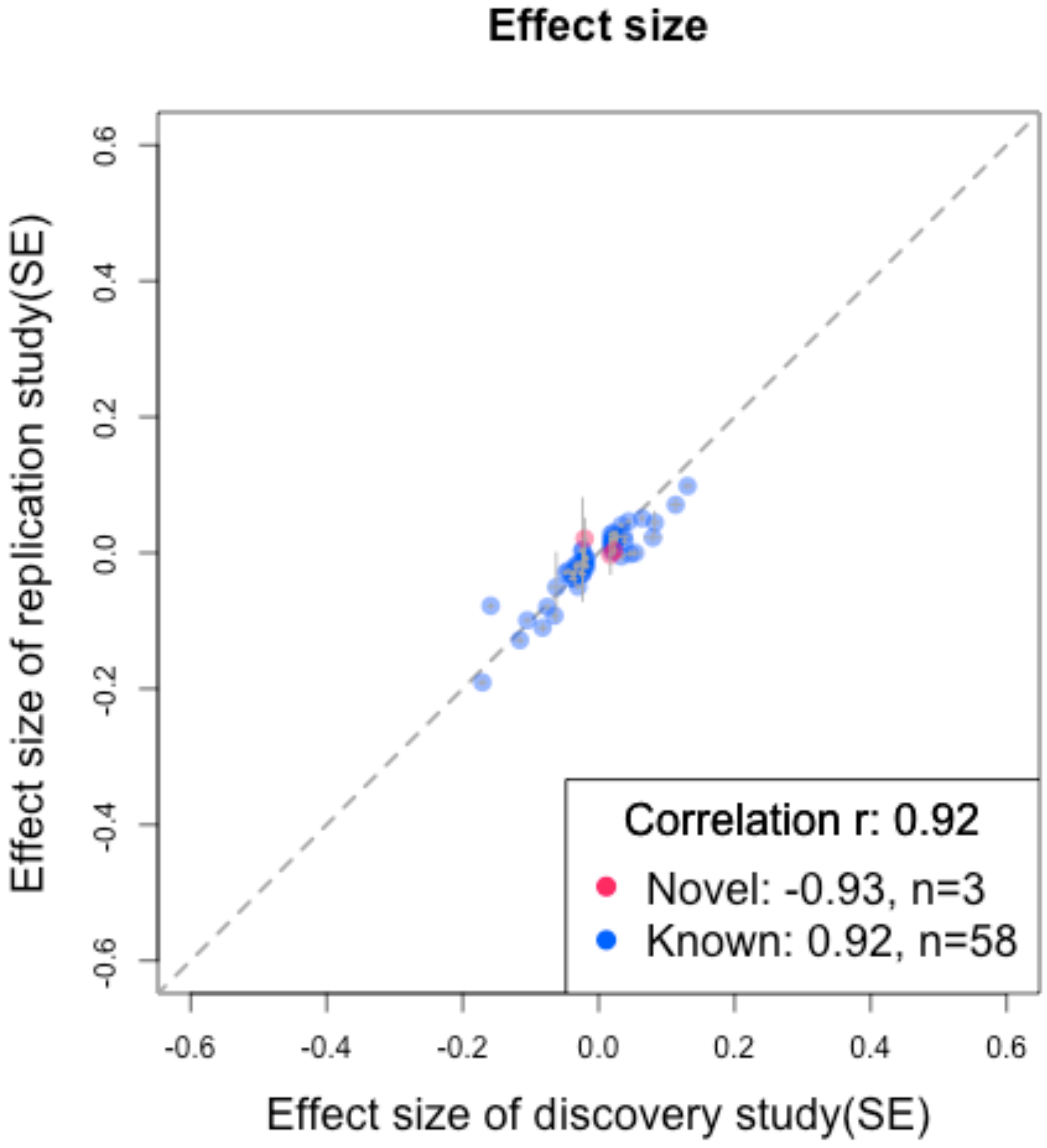

Supplementary Figure 6. Effect allele frequency of 943 associated signals in East Asians and Europeans

(A) scatter plot of effect(alternative) allele frequency(EAF) in East Asian (EAS) and European (EUR) from 1KG P3 or gnomAD database. Pearson's correlation was measured between AAF of EAS and EUR. Variants are colored in 'grey' and 'red' for 'known' and 'novel', respectively. (B) Box plot of AAF by trait categories and populations (EAS at left side and EUR at right side). Points are jittered EAF for the variants. Box plots represent median, 25th, 75th percentiles with whiskers extending to ± 1.5xIQR(interquartile range).

(a) Scatter plot of effect allele frequency (EAS vs. EUR)

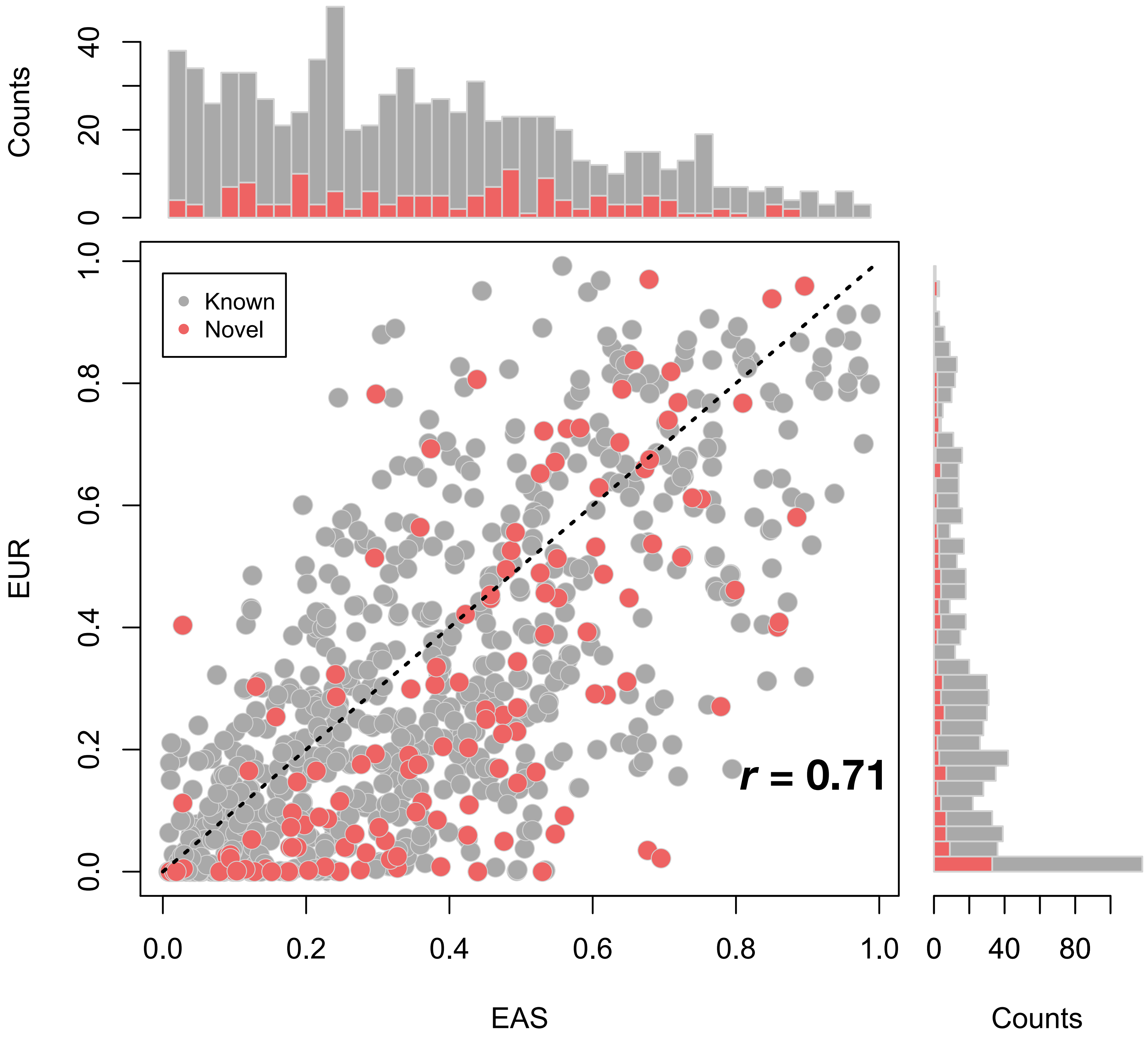

(b) Box plot of effect allele frequency by trait categories and populations

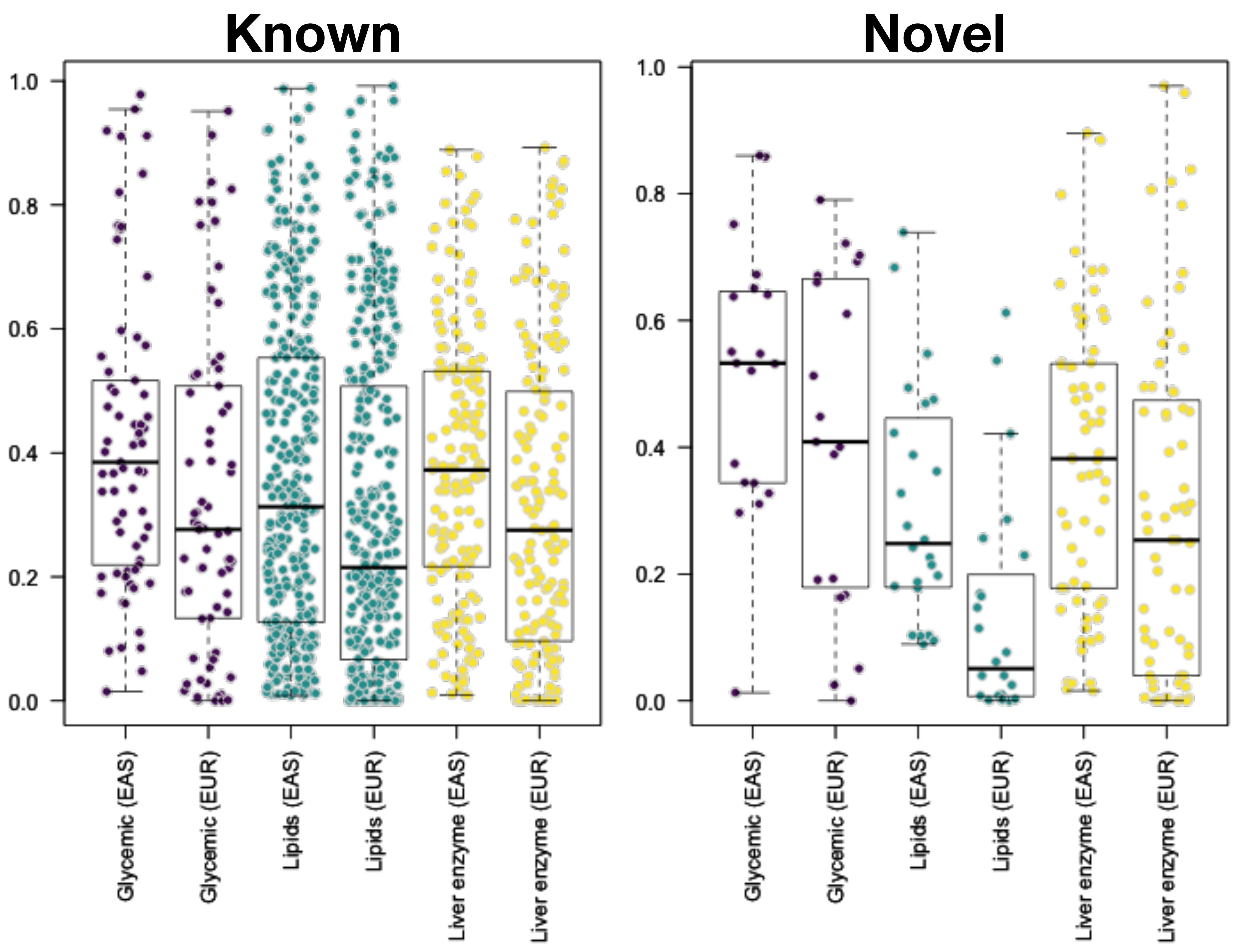

Supplementary Figure 7. Comparison of effect sizes of rare variants between discovery study and replication studies

The points are all the rare variants identified in the discovered study. Effect sizes were compared based on the effect allele of this study. Error bars represent standard errors of effect sizes. At the right corner in the figure, 'n' indicates # of variants

(a) Discovery study vs. KBA (replication, n=22,608 samples)

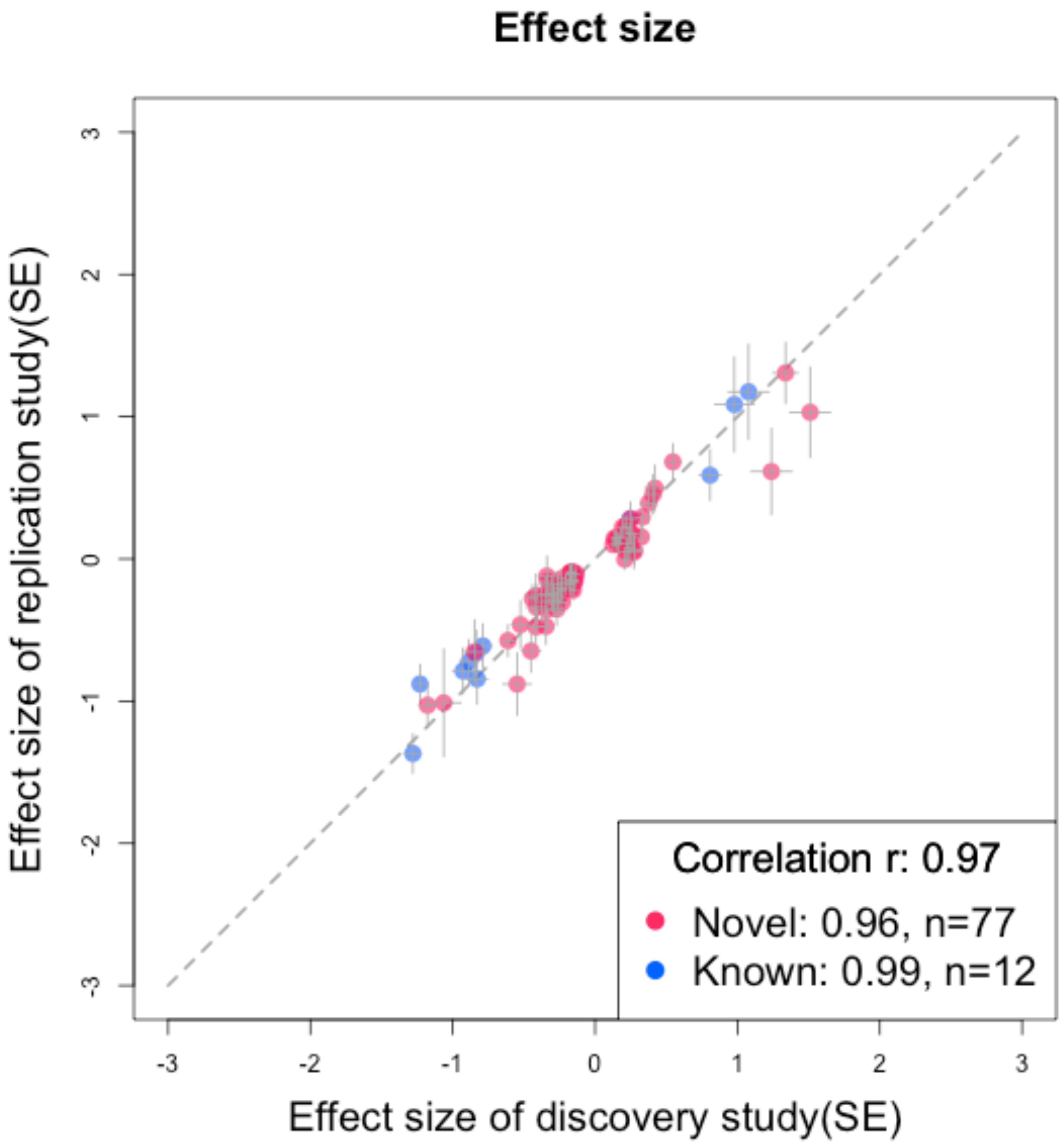

(b) Discovery study vs. UK biobank exome sequencing (replication, n=138,032 samples)

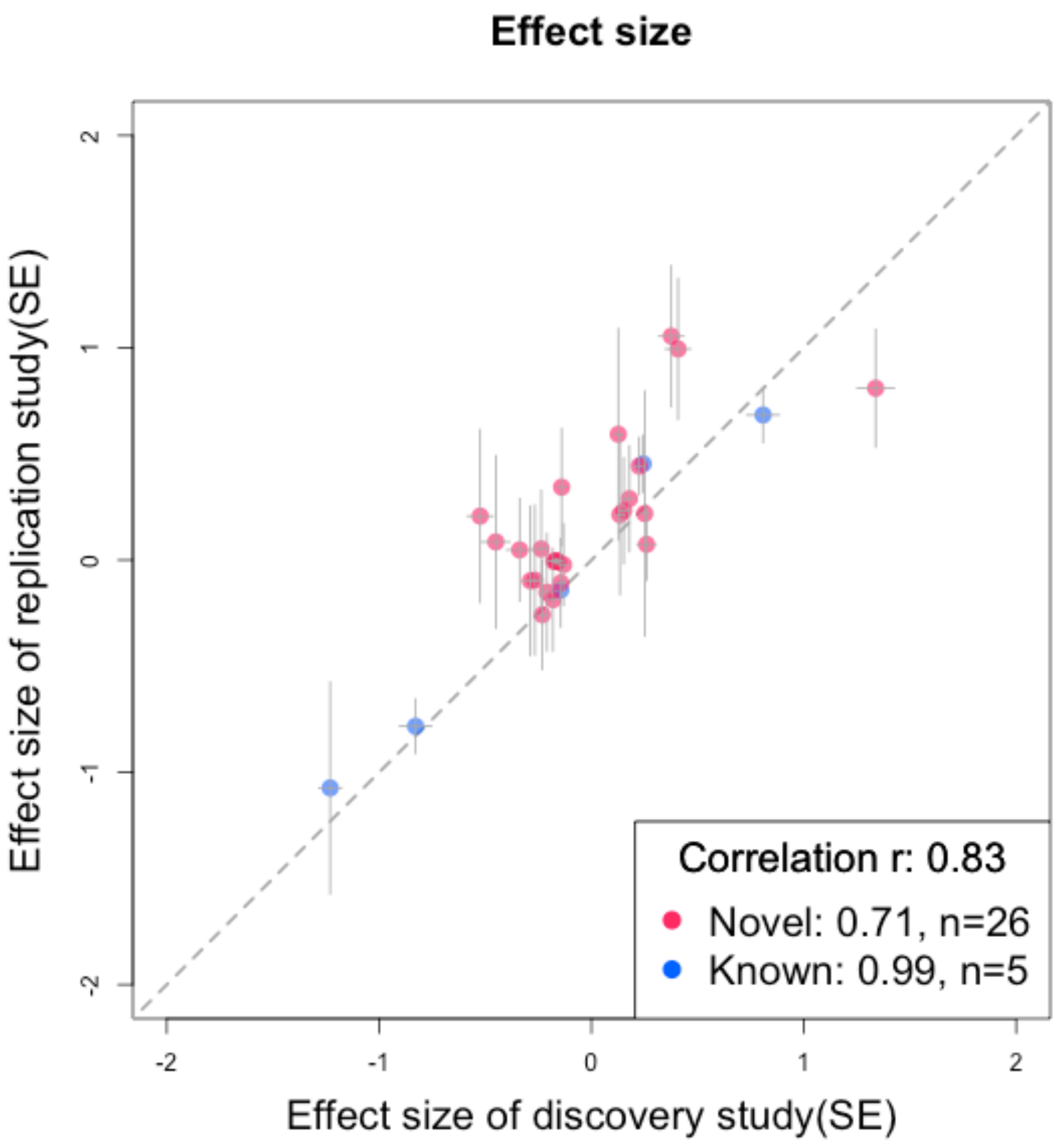

Supplementary Figure 8. Traits varied by common GRS group and status of rare alleles

For more clear representation of overall pattern across groups, outliers were not shown for box plots. Box plots were shown for raw values of each trait. 'N' indicates number of samples for a group. Numbers of top left inner panel indicate number of rare variants with the corresponding risk direction. Risk decreasing group (Risk(-) colored in blue): individuals carrying rare alleles decreasing risks in health problem by decreasing levels of metabolic traits (increasing for HDL). Risk increasing group (Risk(+) colored in red): individuals carrying rare alleles increasing risks in health problem by increasing levels of metabolic traits(decreasing for HDL). Risk complex group (colored in grey): individuals carrying decreasing and increasing rare variants. Reference group (colored in white): Non-carriers of rare variants

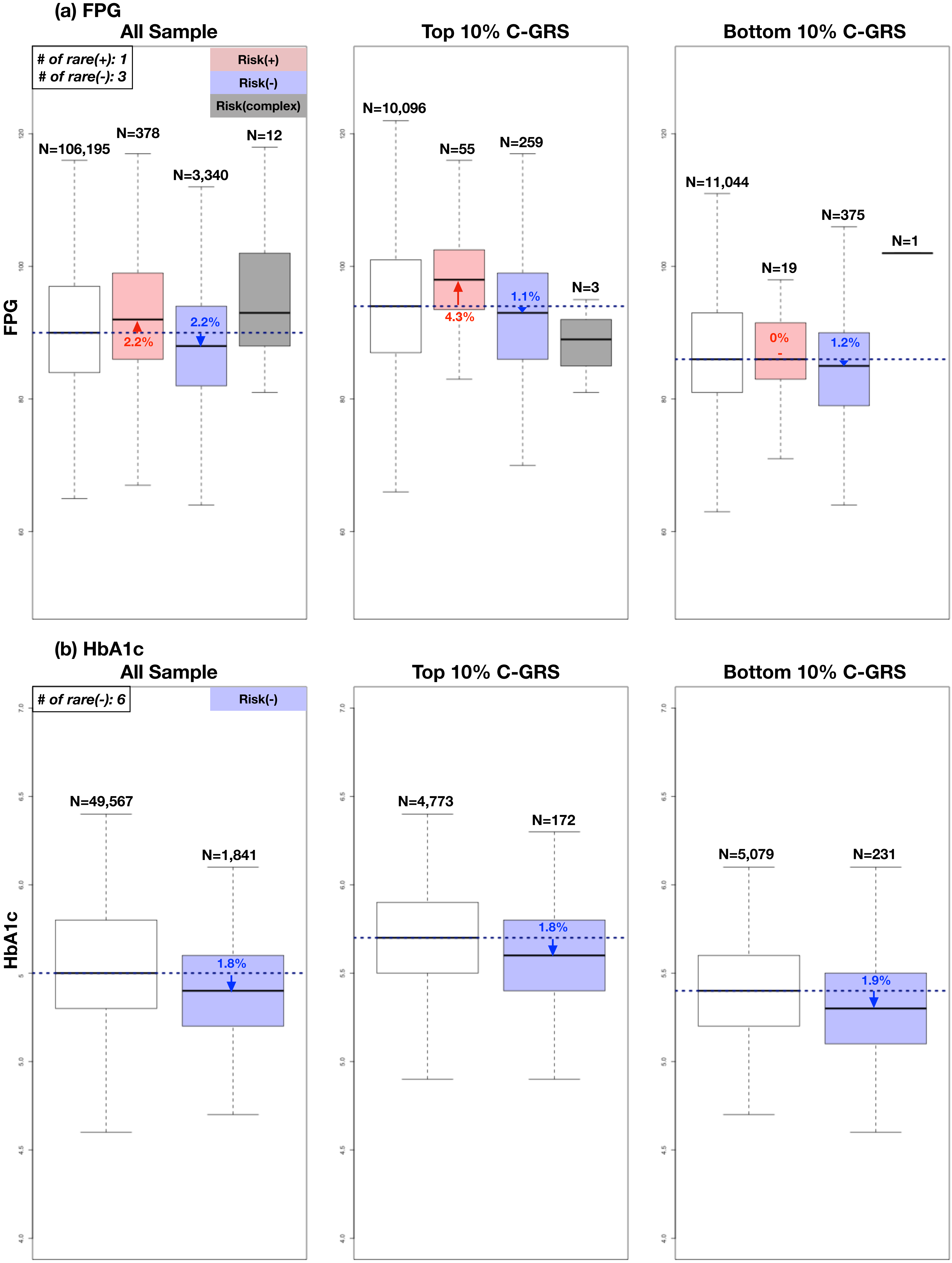

Supplementary Figure 8. Traits varied by common GRS group and status of rare alleles

For more clear representation of overall pattern across groups, outliers were not shown for box plots. Box plots were shown for raw values of each trait. 'N' indicates number of samples for a group. Numbers of top left inner panel indicate number of rare variants with the corresponding risk direction. Risk decreasing group (Risk(-) colored in blue): individuals carrying rare alleles decreasing risks in health problem by decreasing levels of metabolic traits (increasing for HDL). Risk increasing group (Risk(+) colored in red): individuals carrying rare alleles increasing risks in health problem by increasing levels of metabolic traits(decreasing for HDL). Risk complex group (colored in grey): individuals carrying decreasing and increasing rare variants. Reference group (colored in white): Non-carriers of rare variants

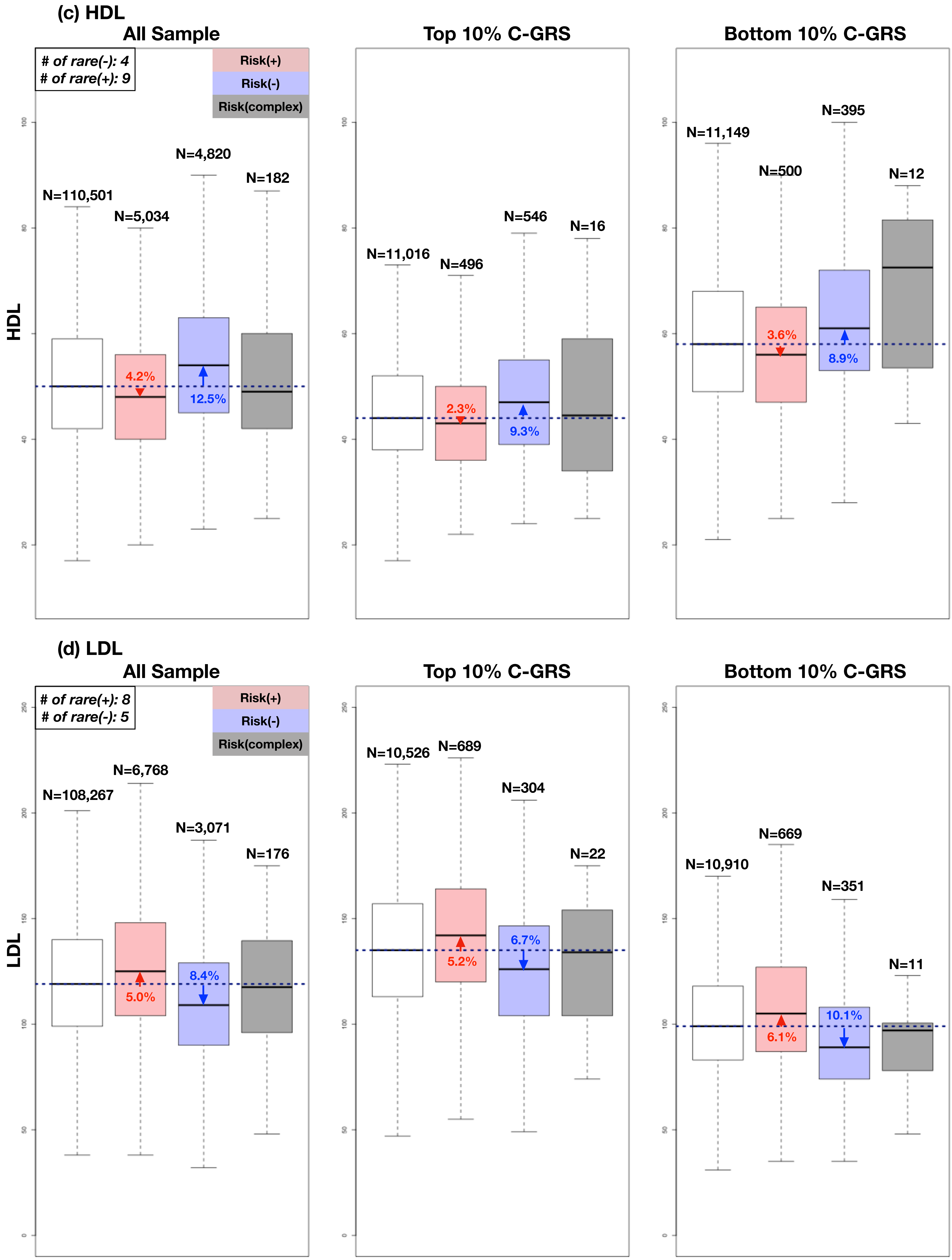

Supplementary Figure 8. Traits varied by common GRS group and status of rare alleles

For more clear representation of overall pattern across groups, outliers were not shown for box plots. Box plots were shown for raw values of each trait. 'N' indicates number of samples for a group. Numbers of top left inner panel indicate number of rare variants with the corresponding risk direction. Risk decreasing group (Risk(-) colored in blue): individuals carrying rare alleles decreasing risks in health problem by decreasing levels of metabolic traits (increasing for HDL). Risk increasing group (Risk(+) colored in red): individuals carrying rare alleles increasing risks in health problem by increasing levels of metabolic traits(decreasing for HDL). Risk complex group (colored in grey): individuals carrying decreasing and increasing rare variants. Reference group (colored in white): Non-carriers of rare variants

(e) TG

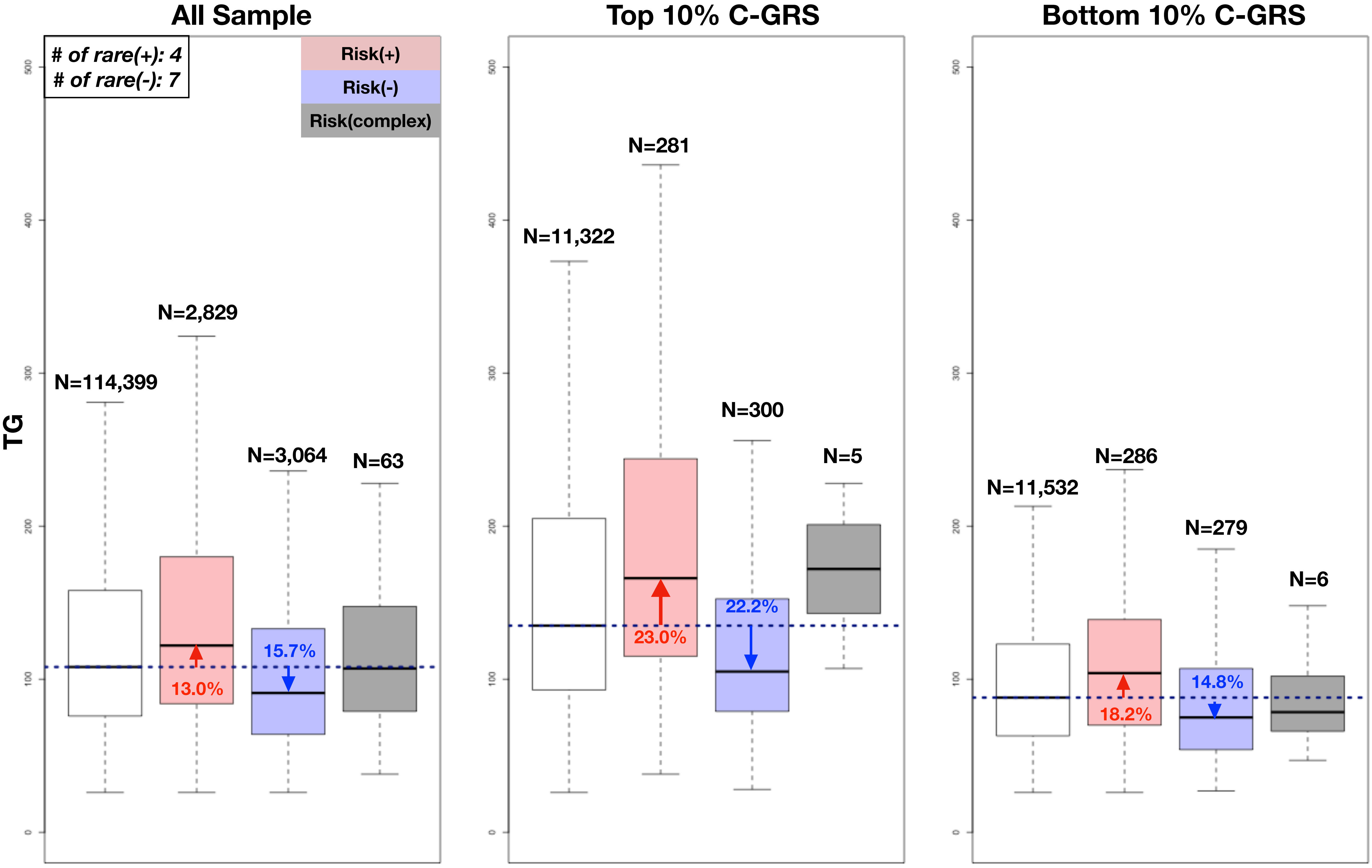

(f) TC

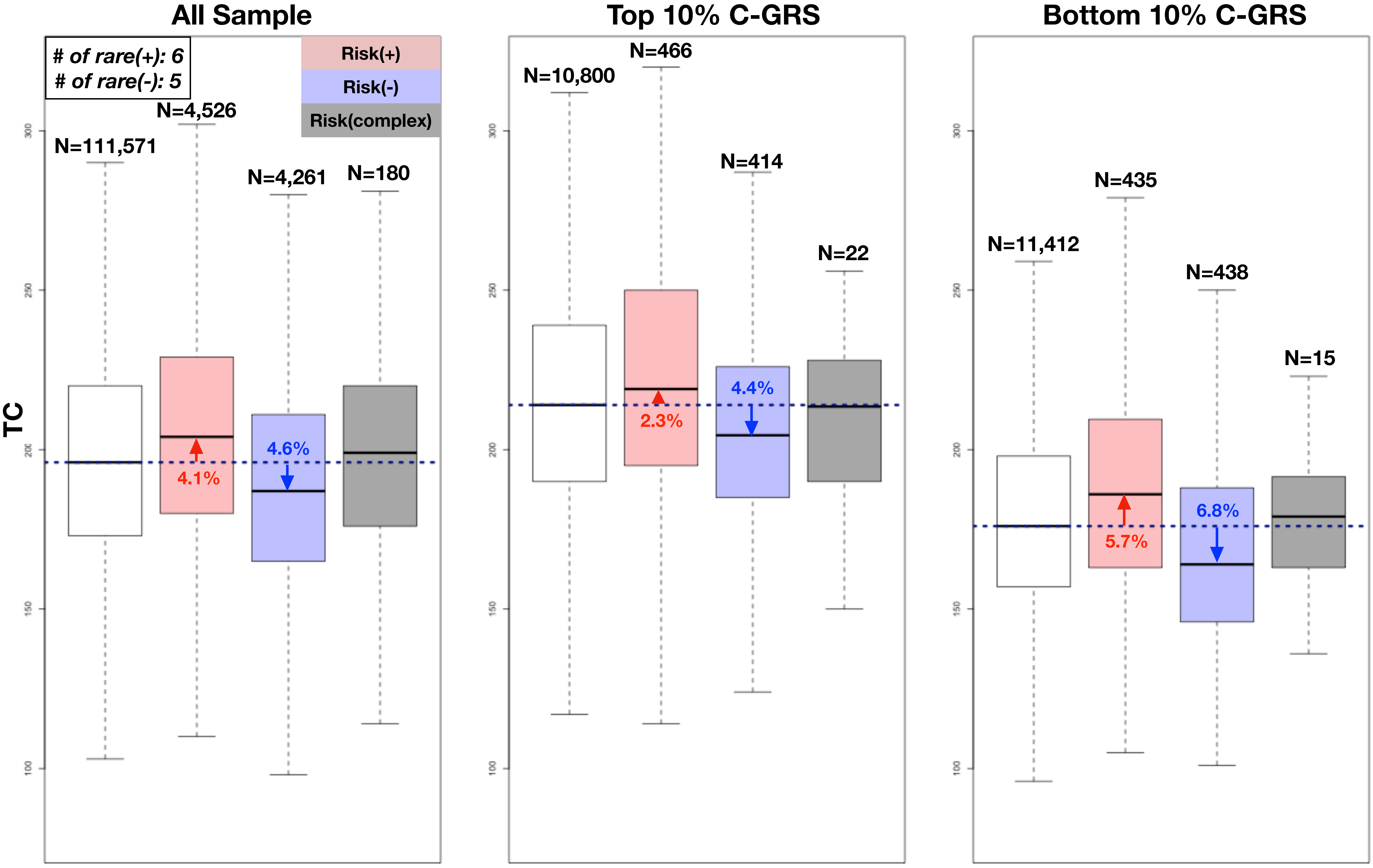

Supplementary Figure 8. Traits varied by common GRS group and status of rare alleles

For more clear representation of overall pattern across groups, outliers were not shown for box plots. Box plots were shown for raw values of each trait. 'N' indicates number of samples for a group. Numbers of top left inner panel indicate number of rare variants with the corresponding risk direction. Risk decreasing group (Risk(-) colored in blue): individuals carrying rare alleles decreasing risks in health problem by decreasing levels of metabolic traits (increasing for HDL). Risk increasing group (Risk(+) colored in red): individuals carrying rare alleles increasing risks in health problem by increasing levels of metabolic traits(decreasing for HDL). Risk complex group (colored in grey): individuals carrying decreasing and increasing rare variants. Reference group (colored in white): Non-carriers of rare variants

(g) ALT

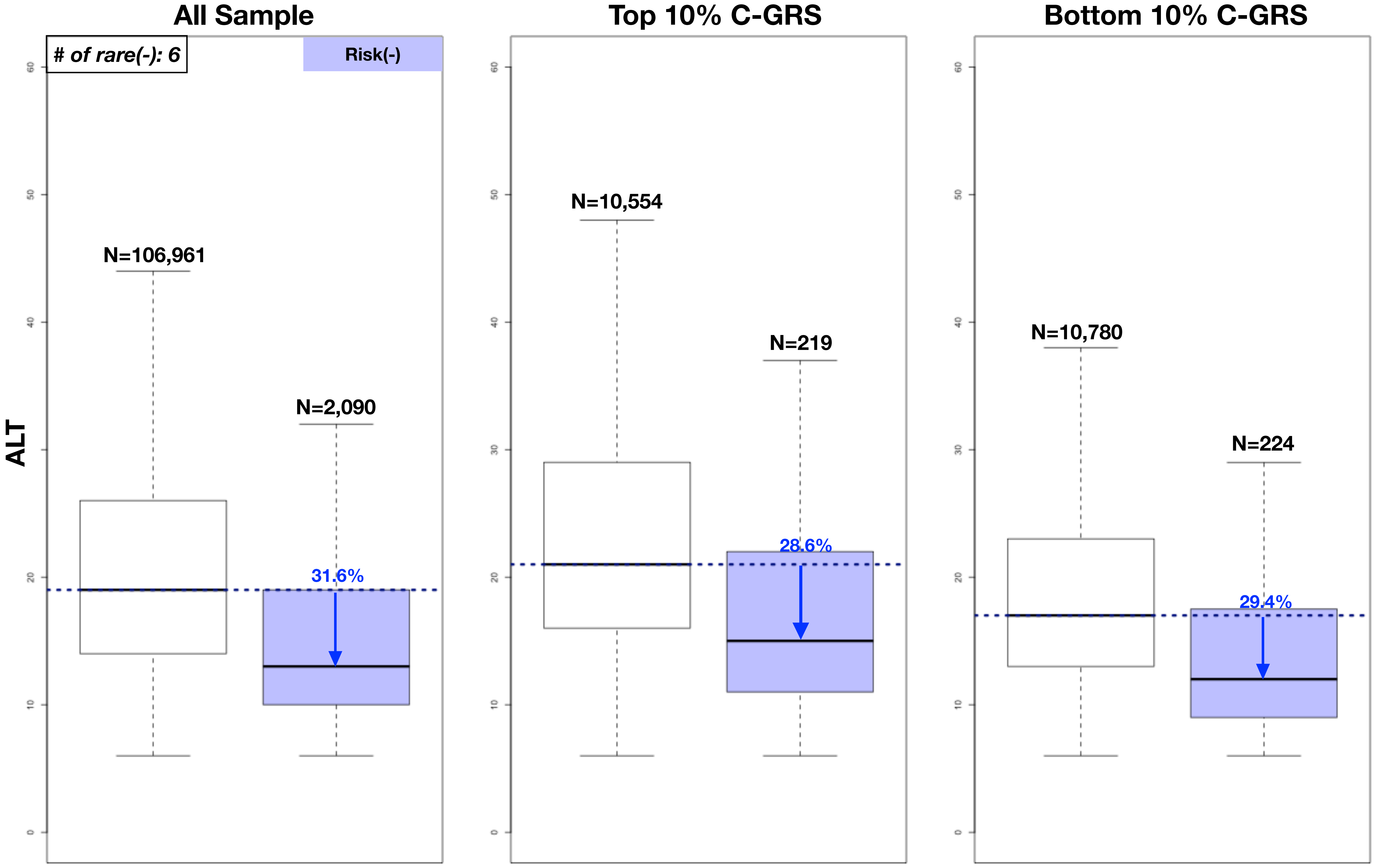

(h) AST

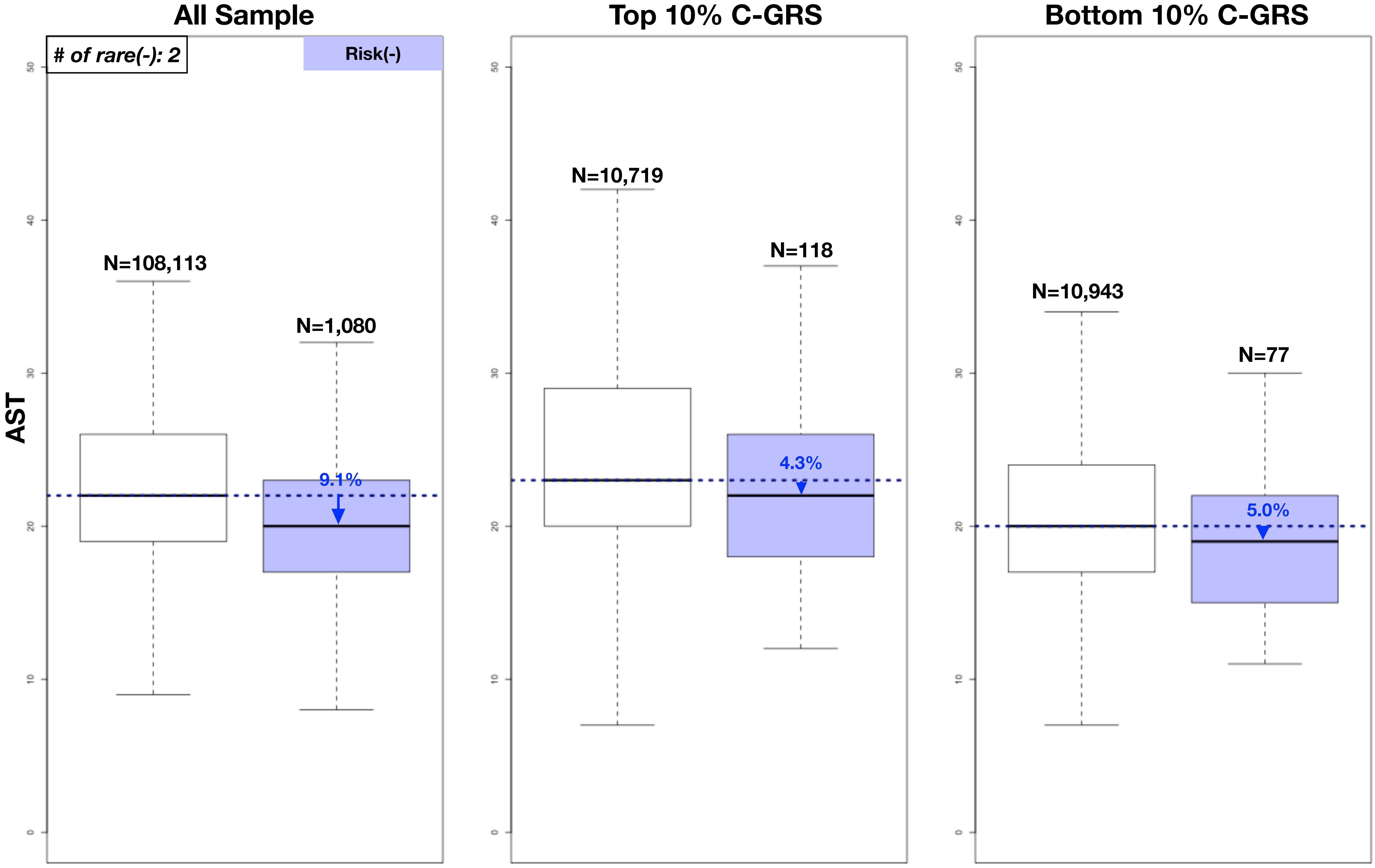

Supplementary Figure 8. Traits varied by common GRS group and status of rare alleles

For more clear representation of overall pattern across groups, outliers were not shown for box plots. Box plots were shown for raw values of each trait. 'N' indicates number of samples for a group. Numbers of top left inner panel indicate number of rare variants with the corresponding risk direction. Risk decreasing group (Risk(-) colored in blue): individuals carrying rare alleles decreasing risks in health problem by decreasing levels of metabolic traits (increasing for HDL). Risk increasing group (Risk(+) colored in red): individuals carrying rare alleles increasing risks in health problem by increasing levels of metabolic traits(decreasing for HDL). Risk complex group (colored in grey): individuals carrying decreasing and increasing rare variants. Reference group (colored in white): Non-carriers of rare variants

(i) GGT

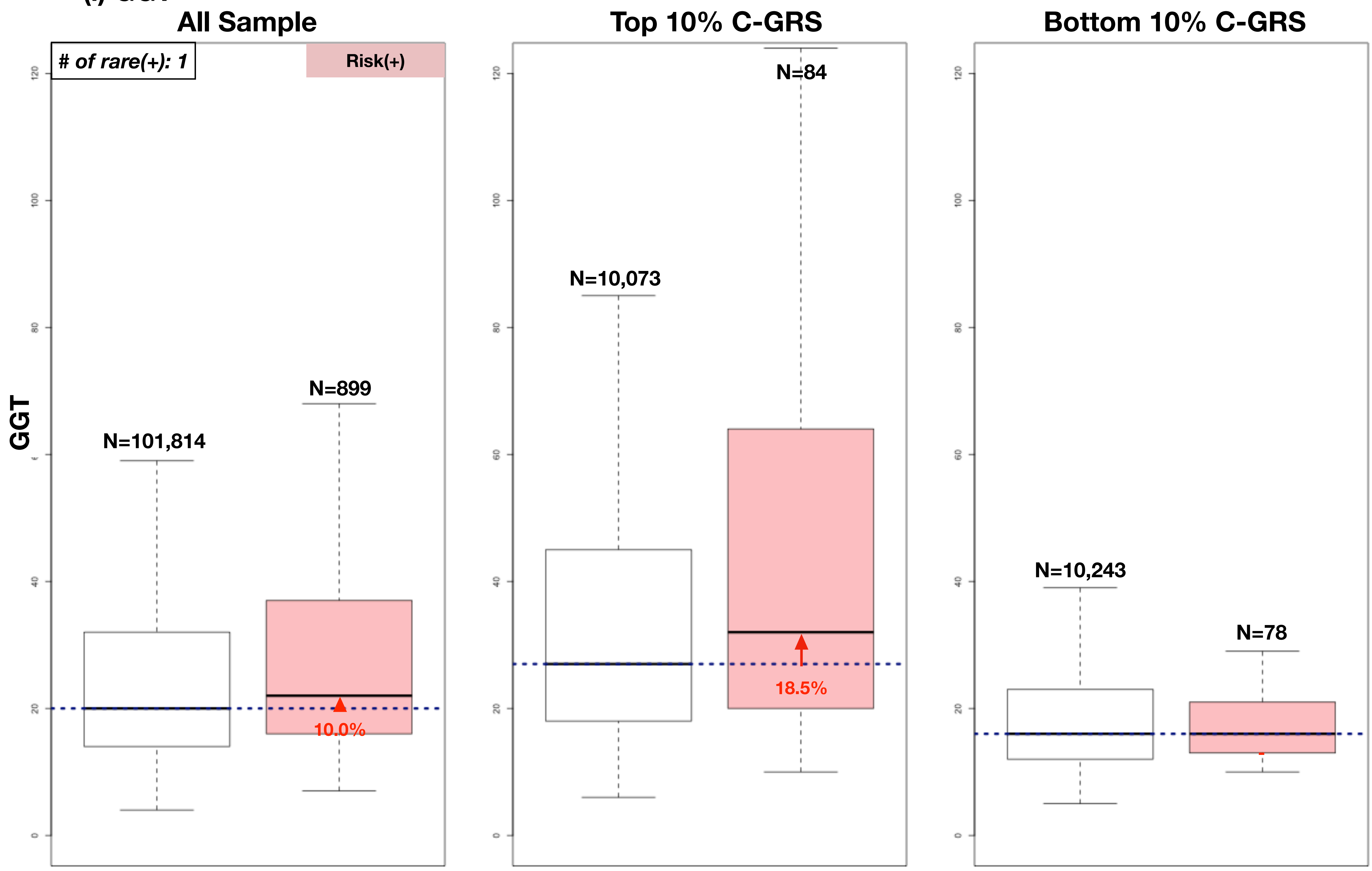

Supplementary Figure 9. Mean levels of glycemic traits by glycemic trait related GRS

Samples were grouped into 10 groups based on GRS scores in an increasing order. CV-GRS indicates GRS using common lead variants identified in this study. For each GRS bin, mean levels of FPG (A) or HbA1c (B) was calculated. HbA1c_C1_GRS indicates HbA1c GRS using only glycemic components. HbA1c_C2_GRS represents HbA1c GRS using erythrocytic components. Symbol were colored in blue (FPG_GRS), green (HbA1c_GRS), yellow (HbA1c_C1_GRS), grey (HbA1c_C2_GRS), and red (T2D_GRS).

(a) FPG

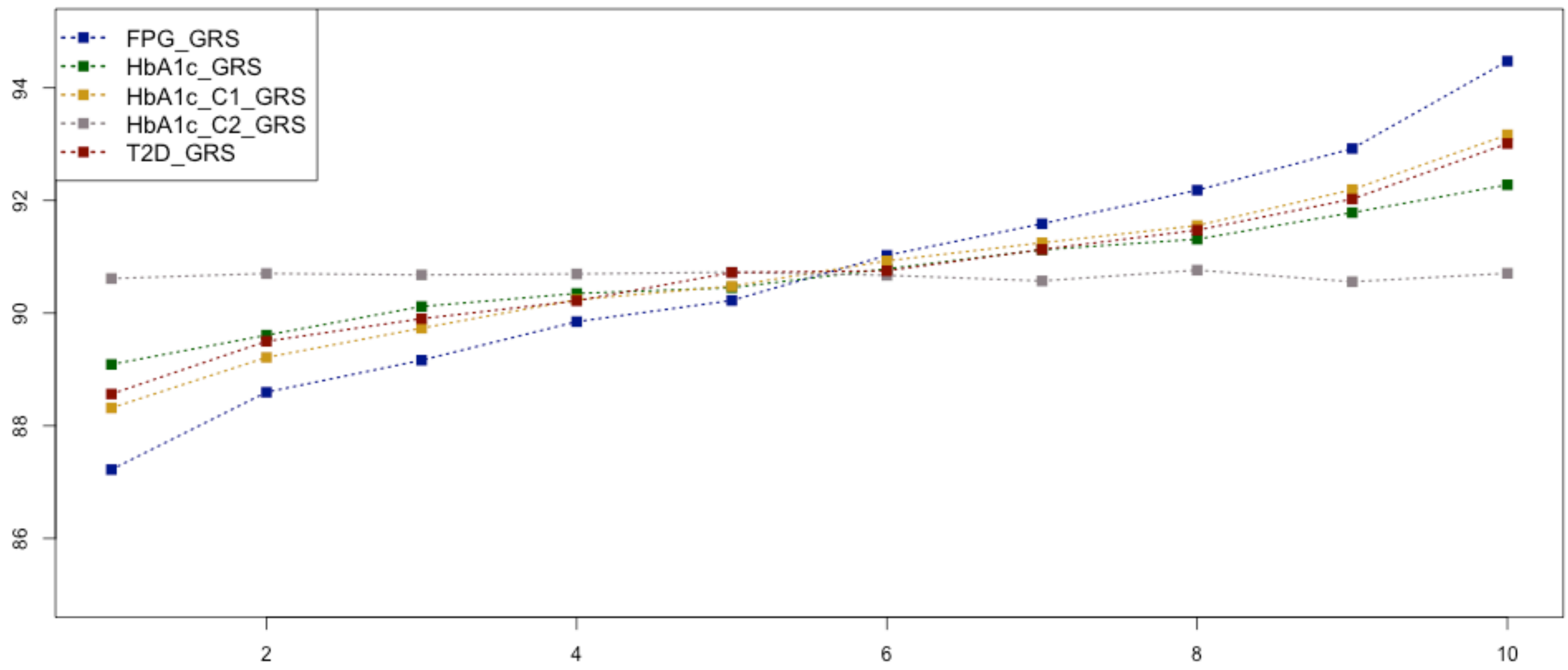

(b) HbA1c

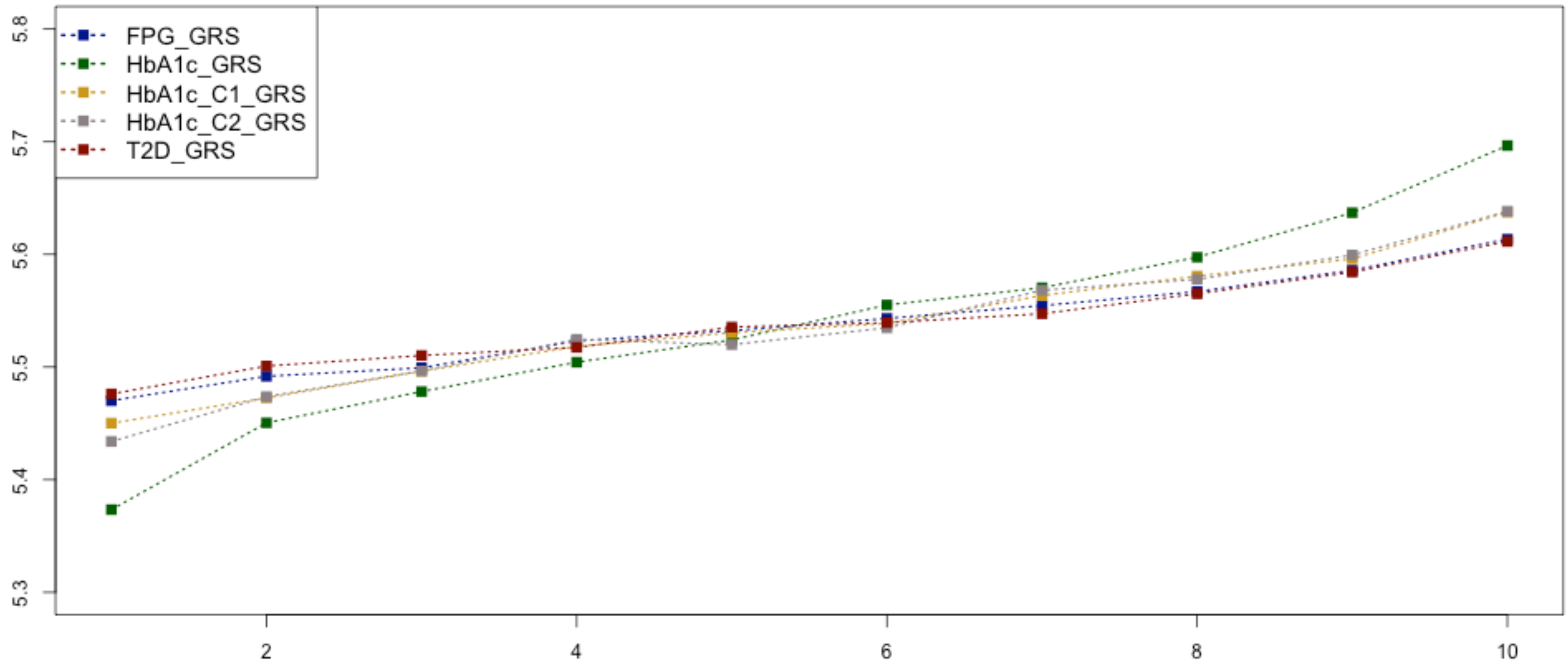

Supplementary Figure 10. Prevalence of T2D in GRS groups stratified by the presence of a rare protective allele

For GRS groups, T2D prevalence was calculated for non-carriers and carriers of a rare protective allele.

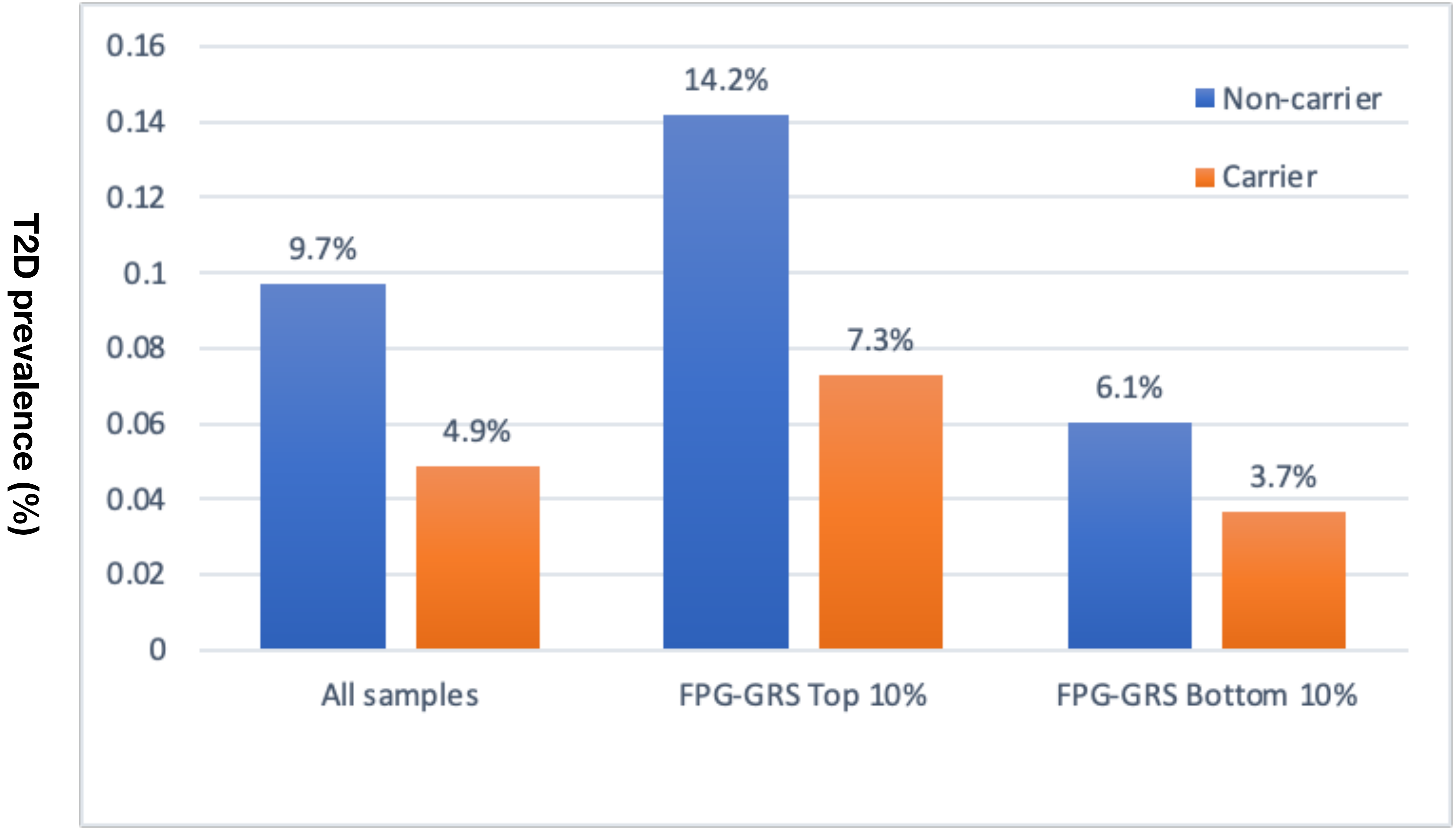

Supplementary Figure 11. Interplay of common and rare variants in inherited risk of T2D (PRS)

After sorting T2D PRS scores in an increasing order, T2D-PRS bins were categorized as 1st bin (0~20%), 2nd bin (20~80%), and 3rd bin (80%~100%) for clear representation. For rare allele carriers (dashed lines) and non-carriers (solid lines), all 126K samples were categorized into three T2D-PRS bins and T2D prevalence was calculated for rare allele carriers and non-carriers, separately. Lines were colored in red (FPG-PRS 80~100%) and blue (FPG-PRS 20~80%).

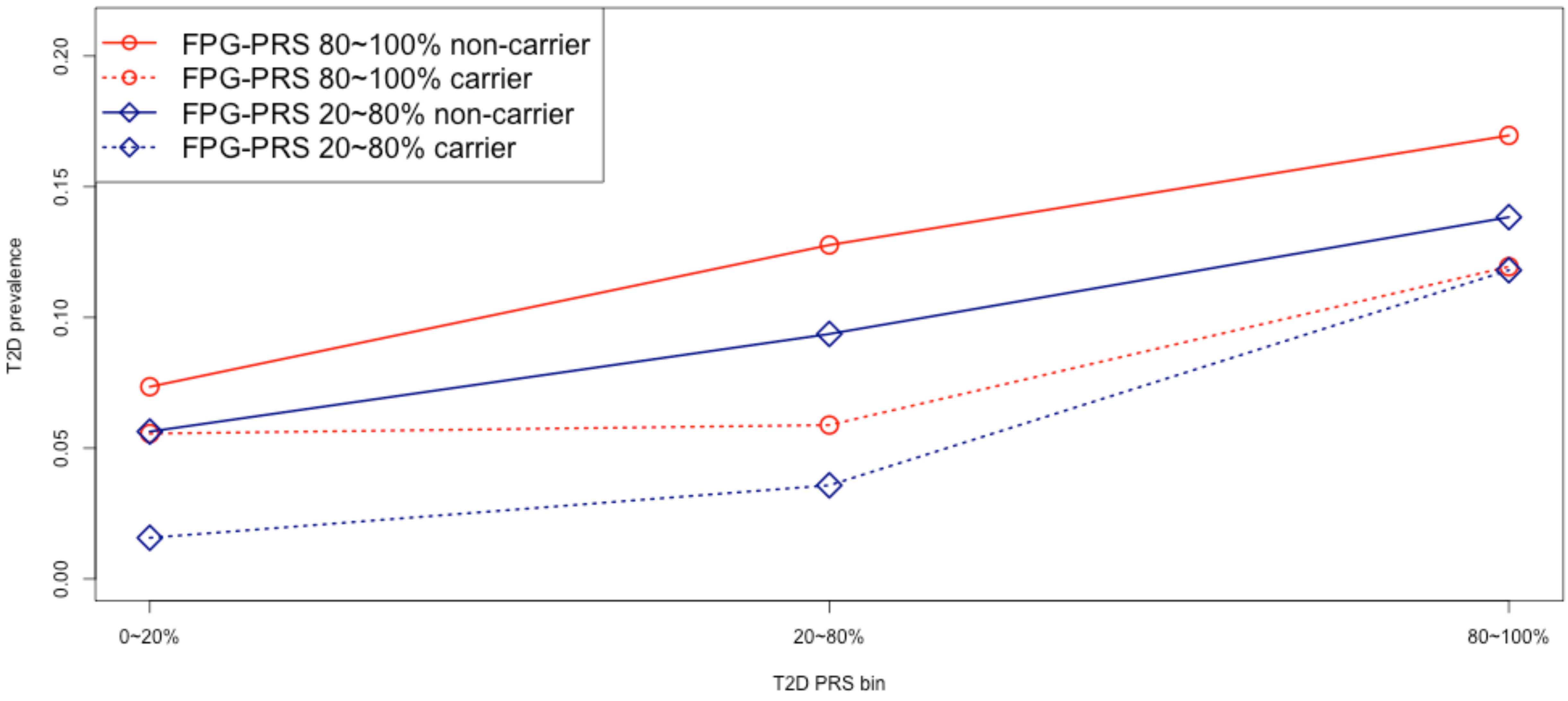

Supplementary Figure 12. Scatter plot of minor allele frequency of rare variants

Scatter plot of MAF (A) KBA vs. 2,579 Korean sequenced samples, (B) KBA vs. gnomAD EAS, (C) KBA vs. 1KG EAS
A dot indicate a variant. A variant was colored in blue if MAF difference < 0.5%, otherwise colored in red

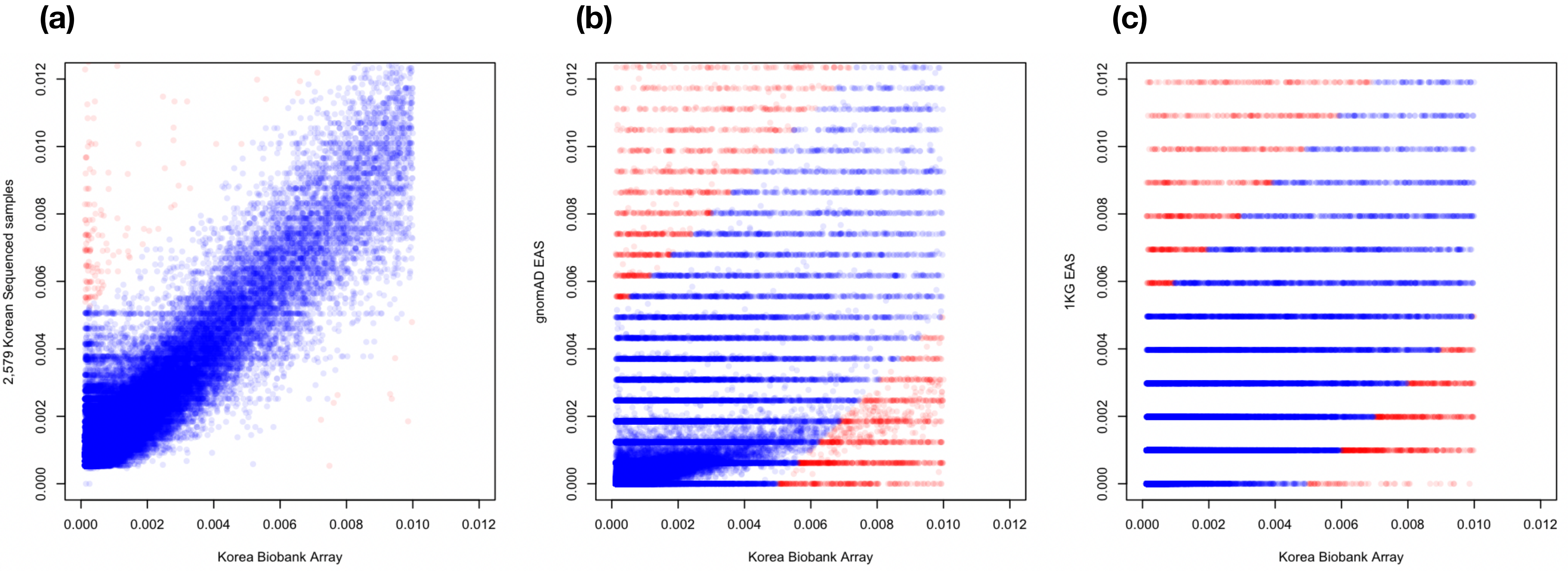

Supplementary Figure 13. Cluster plots of rare variants (Discovery study, KBA)

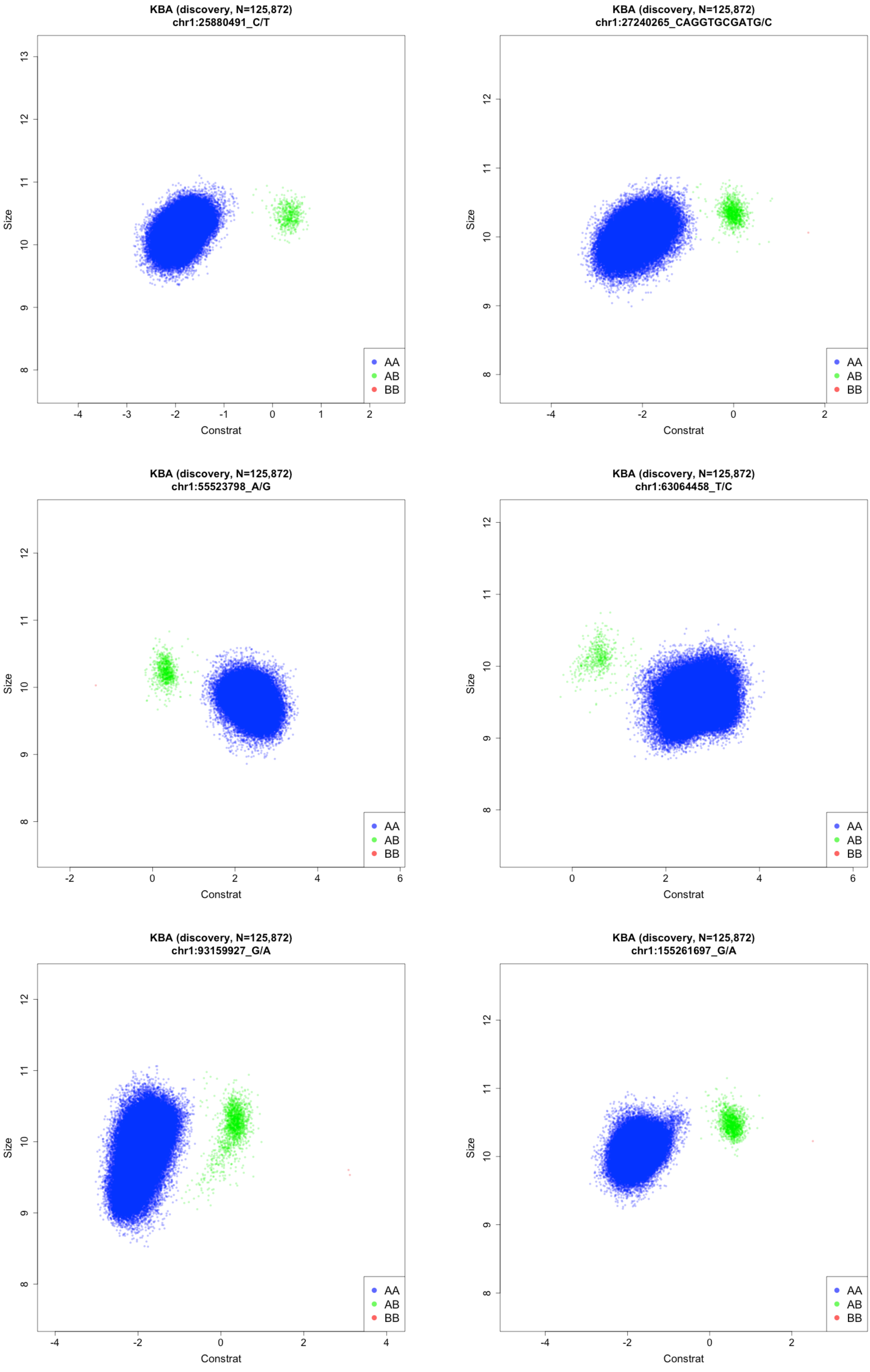

Supplementary Figure 13. Cluster plots of rare variants (Discovery study, KBA)

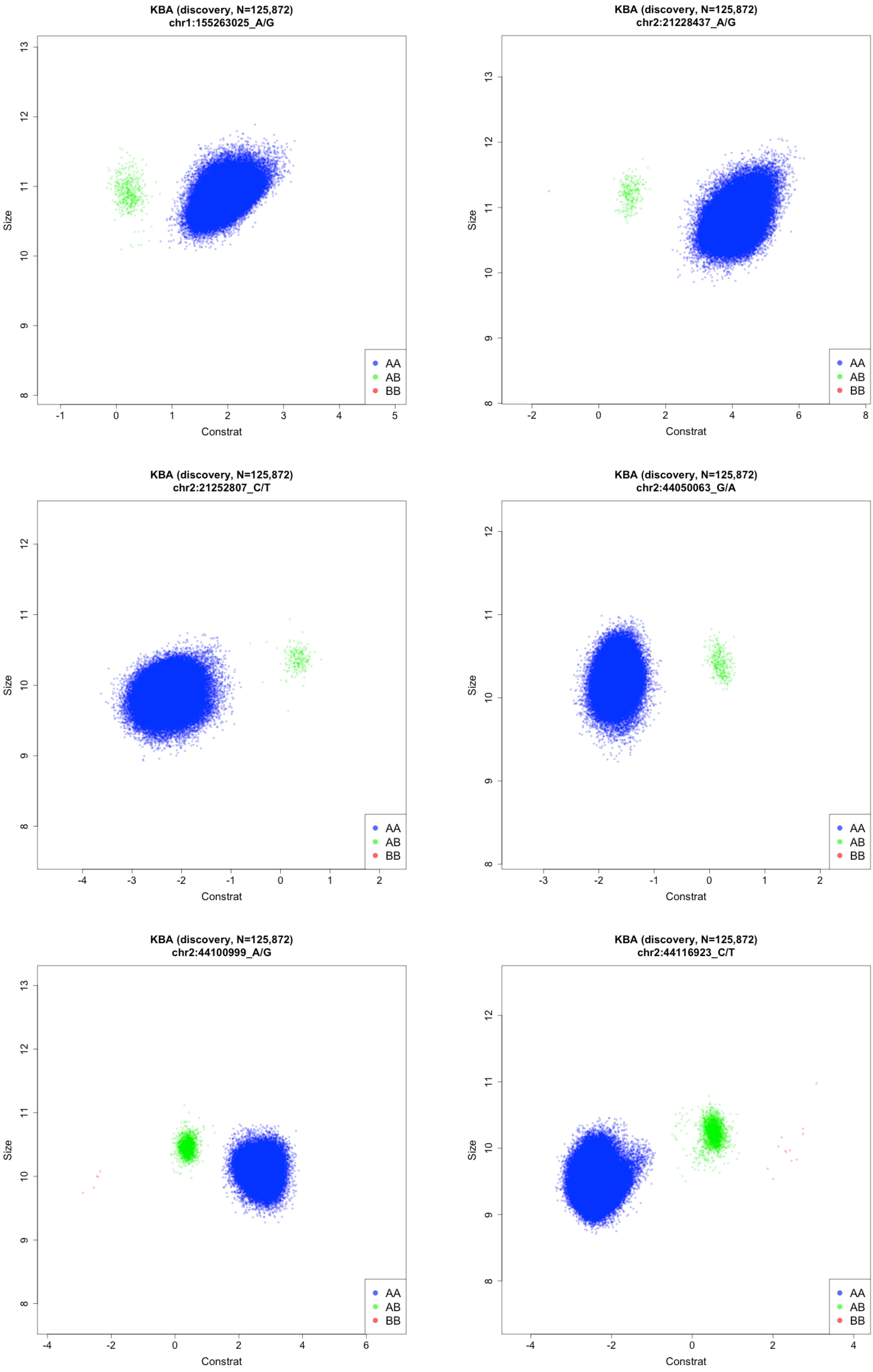

Supplementary Figure 13. Cluster plots of rare variants (Discovery study, KBA)

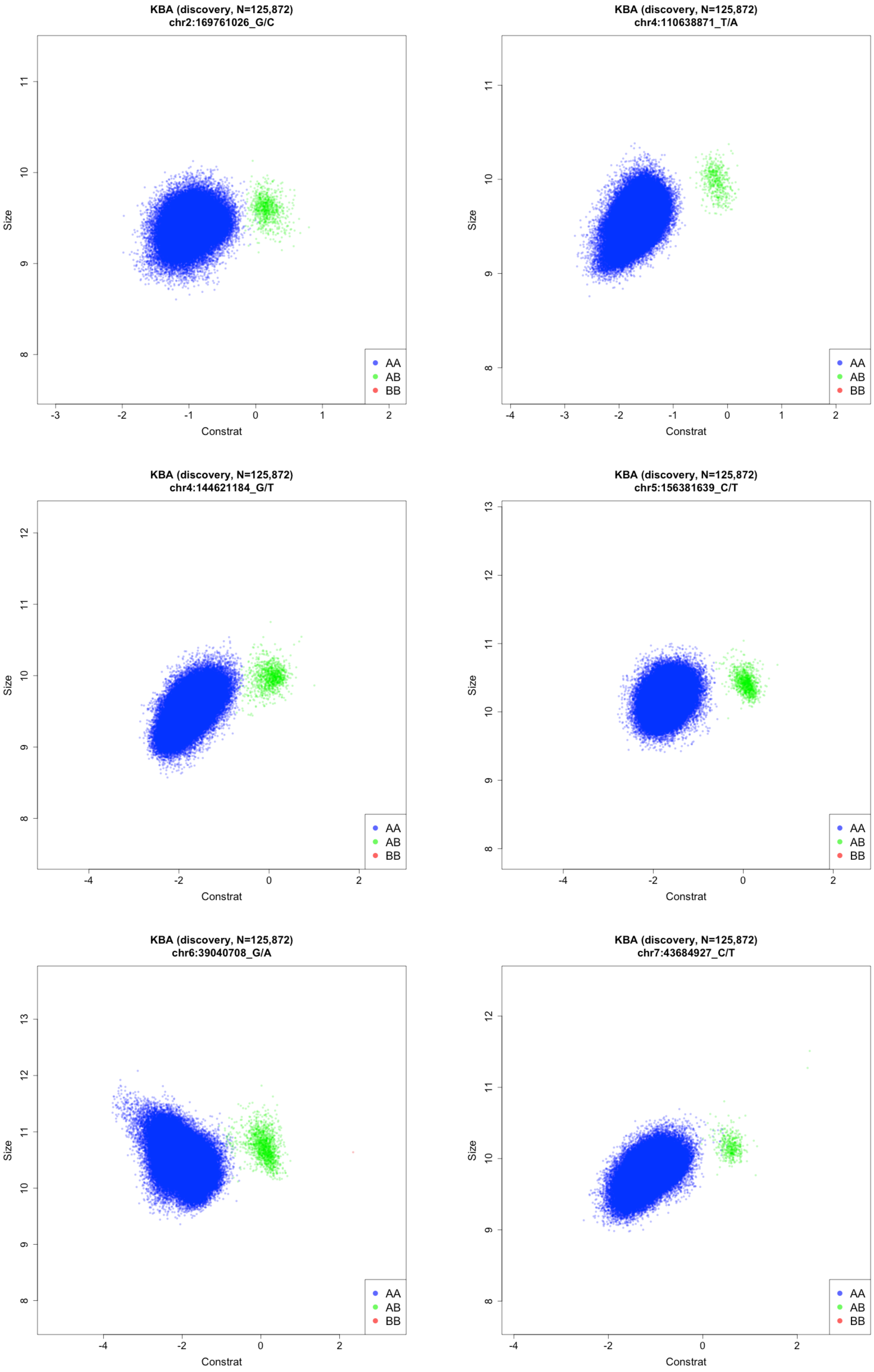

Supplementary Figure 13. Cluster plots of rare variants (Discovery study, KBA)

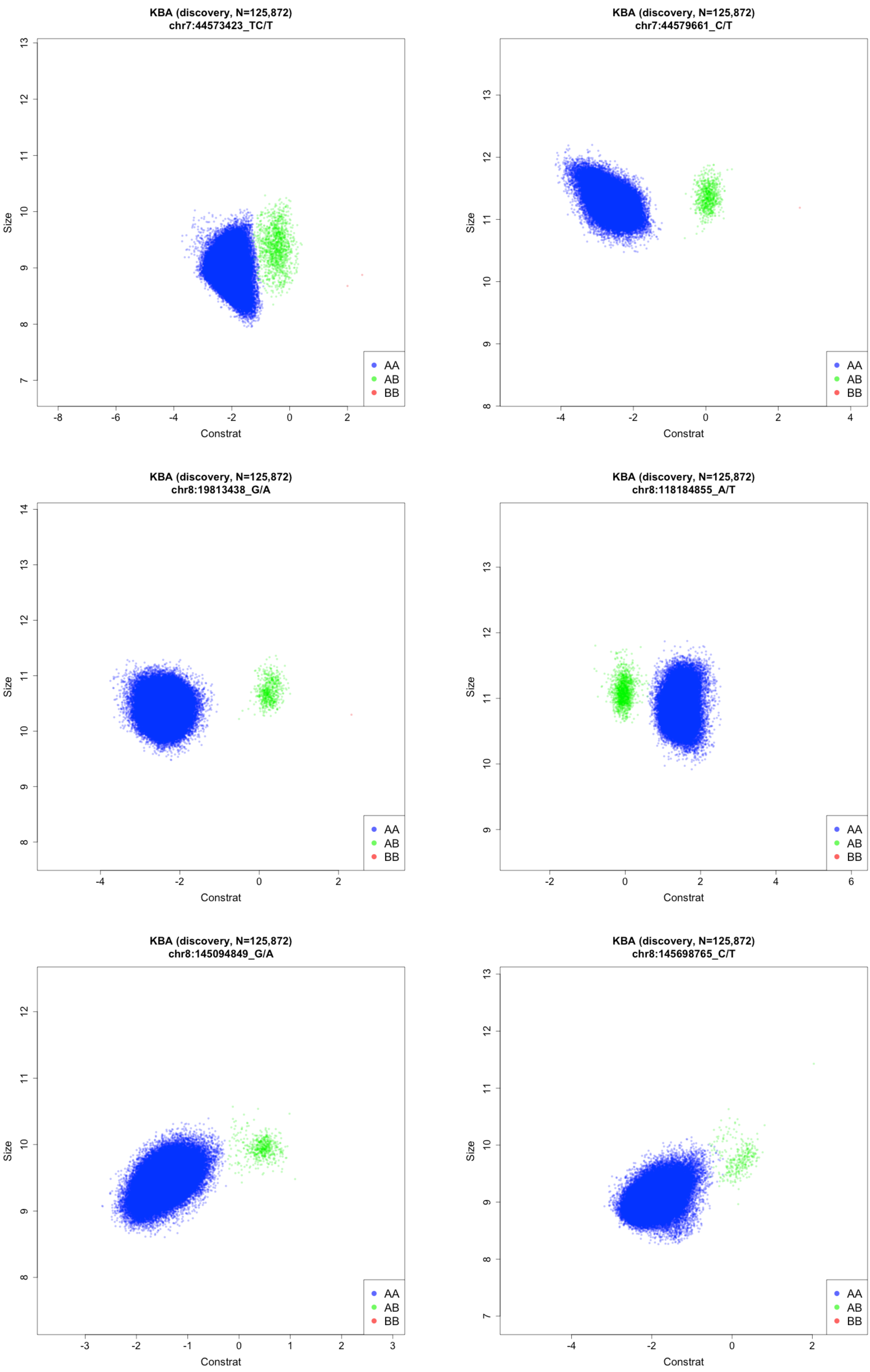

Supplementary Figure 13. Cluster plots of rare variants (Discovery study, KBA)

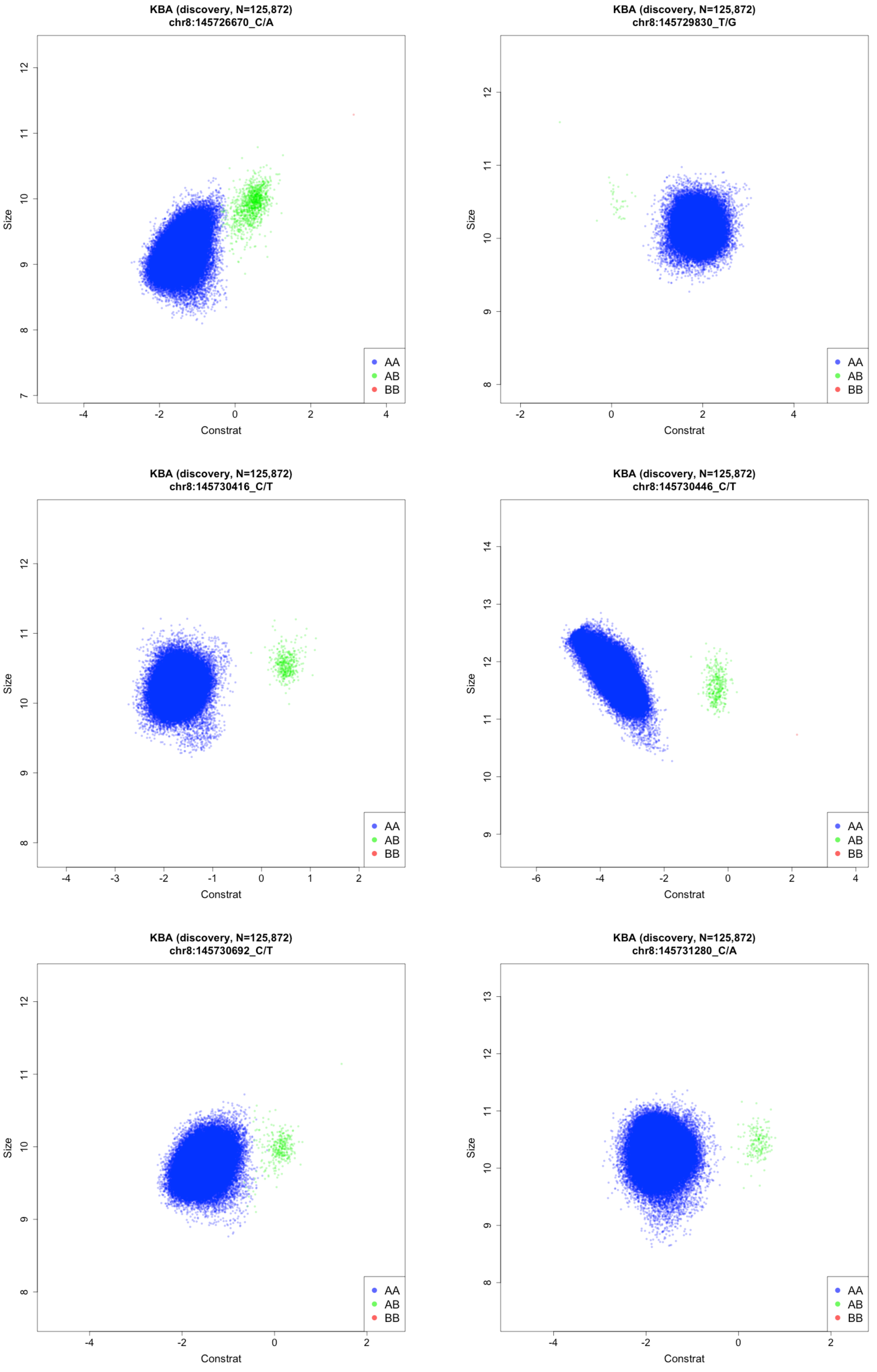

Supplementary Figure 13. Cluster plots of rare variants (Discovery study, KBA)

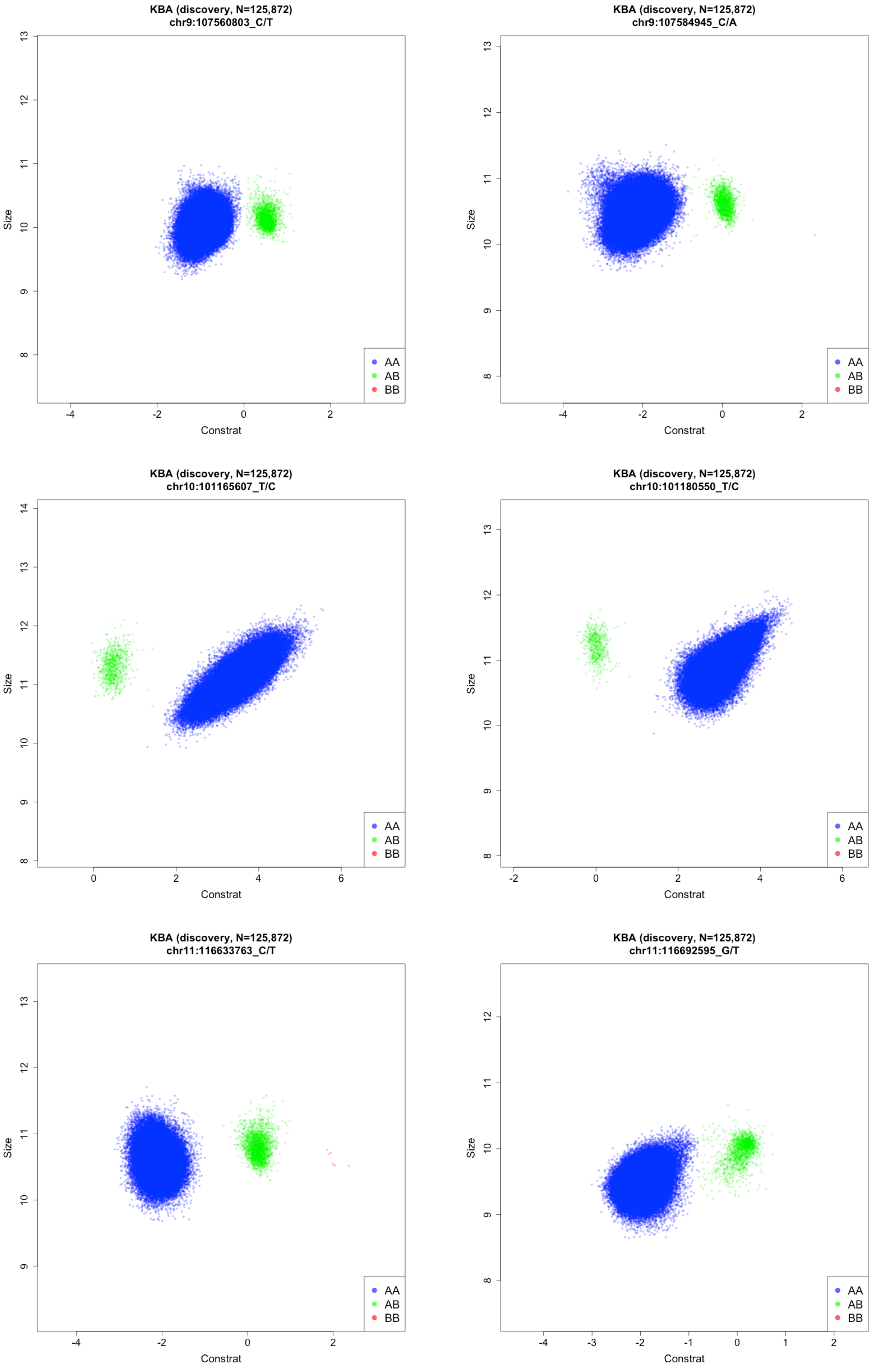

Supplementary Figure 13. Cluster plots of rare variants (Discovery study, KBA)

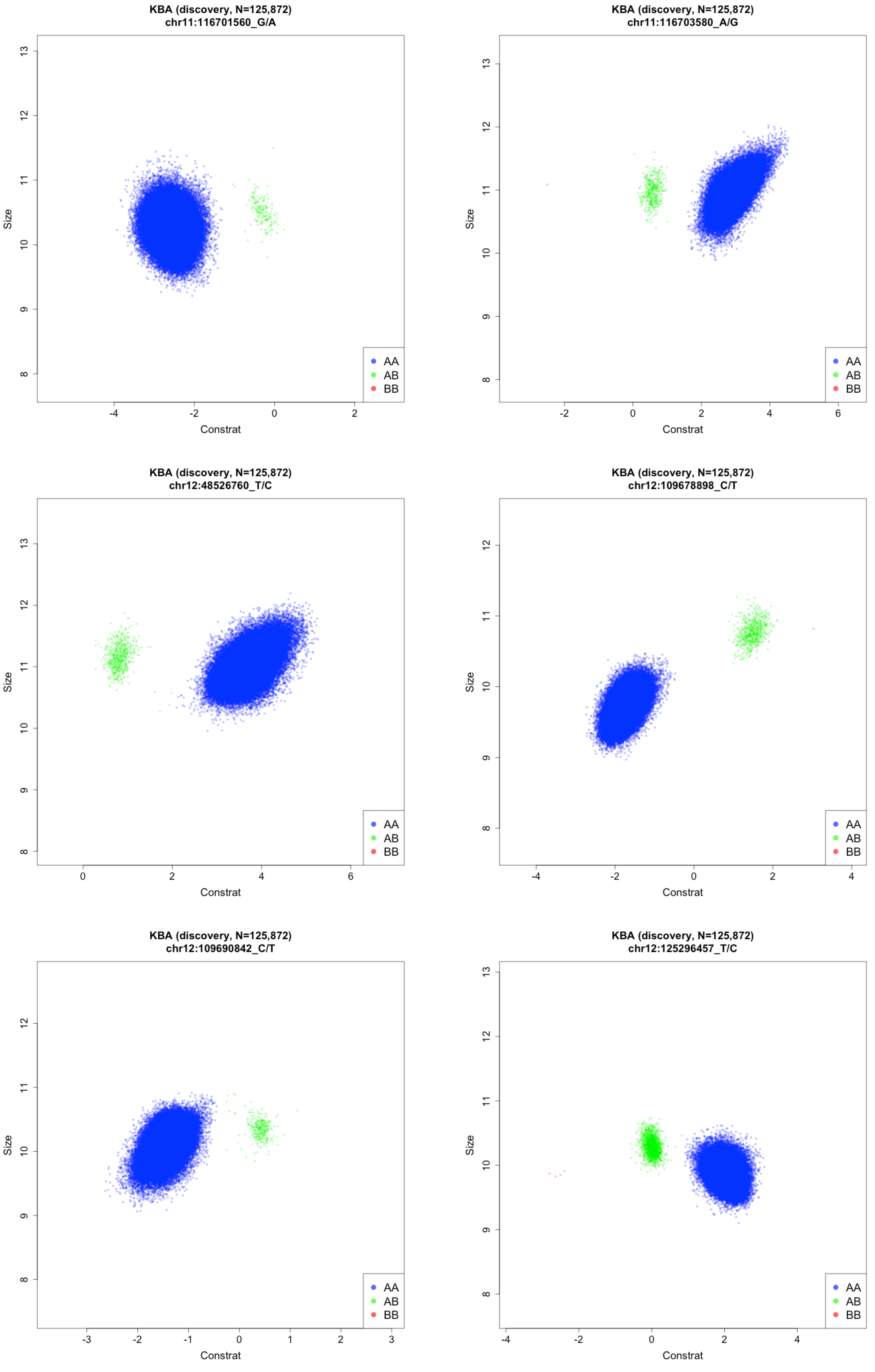

Supplementary Figure 13. Cluster plots of rare variants (Discovery study, KBA)

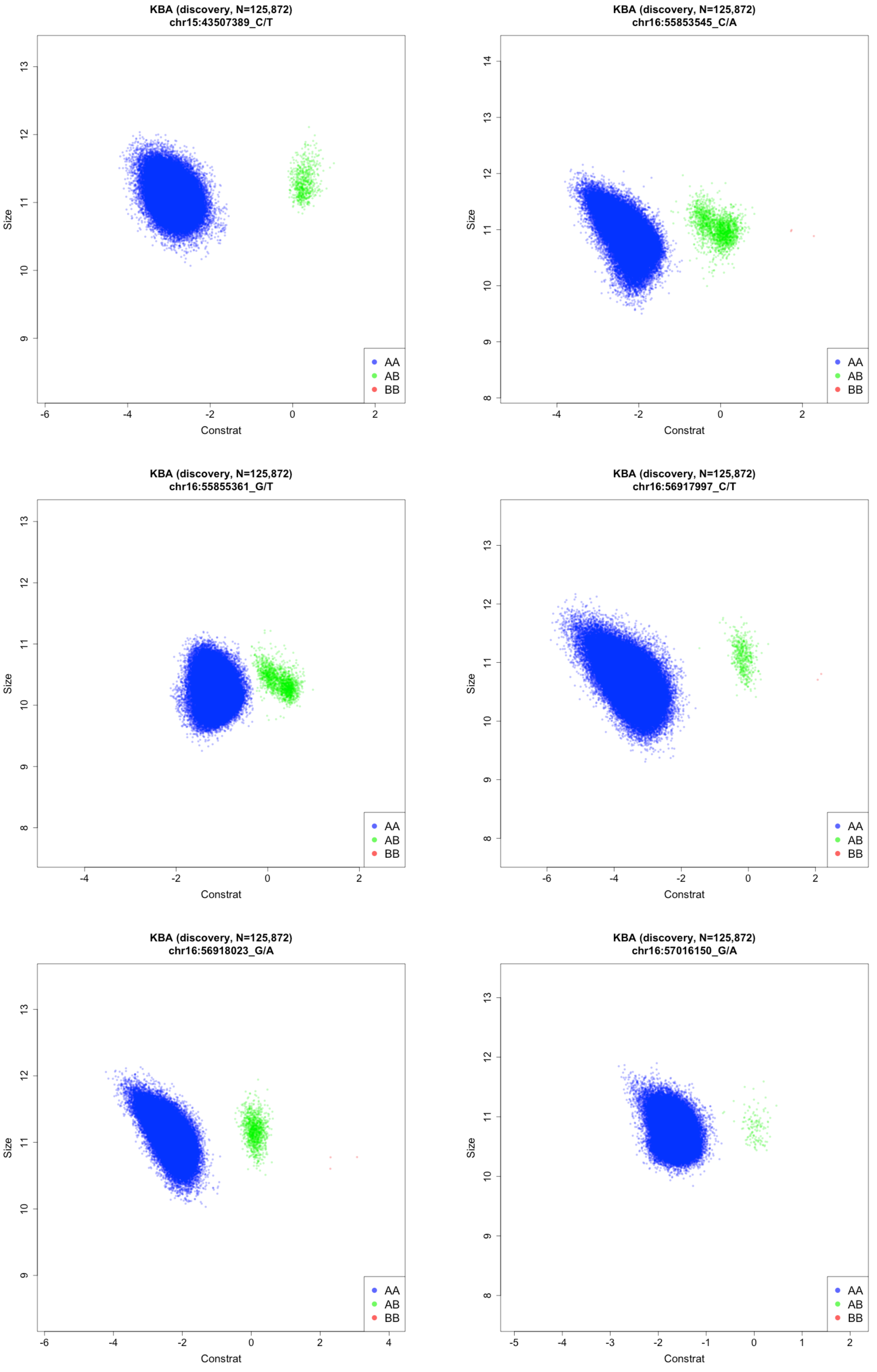

Supplementary Figure 13. Cluster plots of rare variants (Discovery study, KBA)

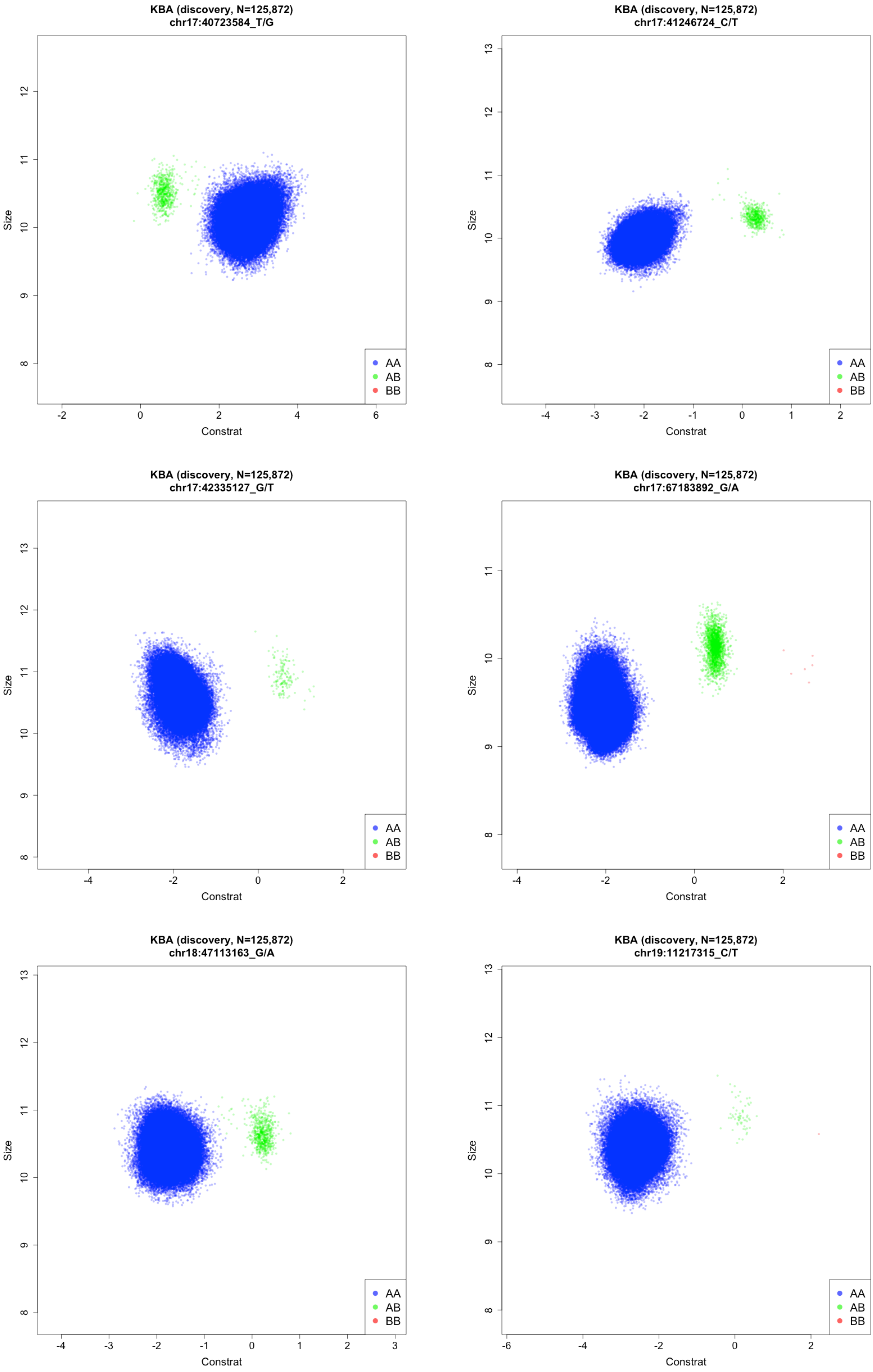

Supplementary Figure 13. Cluster plots of rare variants (Discovery study, KBA)

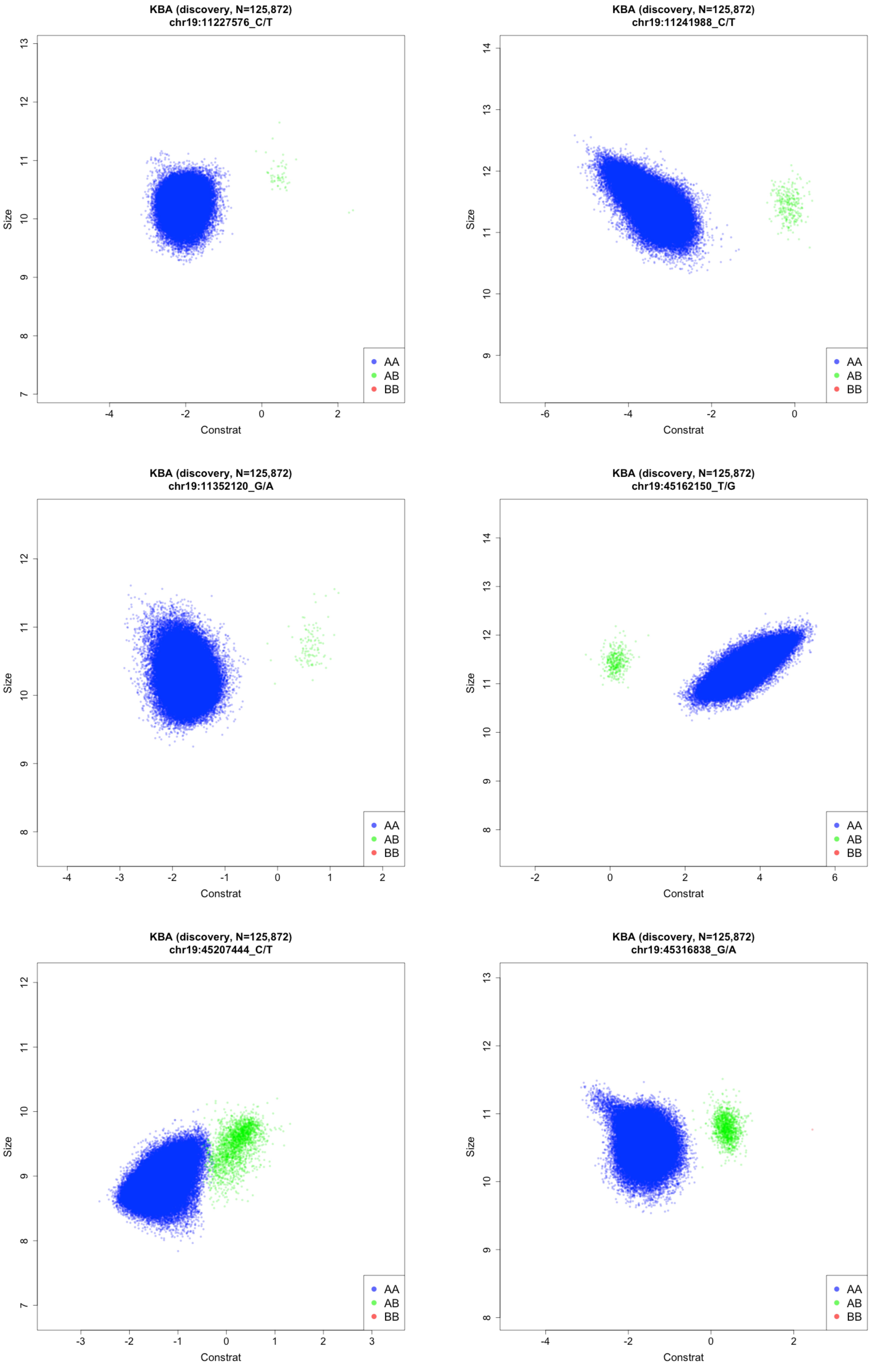

Supplementary Figure 13. Cluster plots of rare variants (Discovery study, KBA)

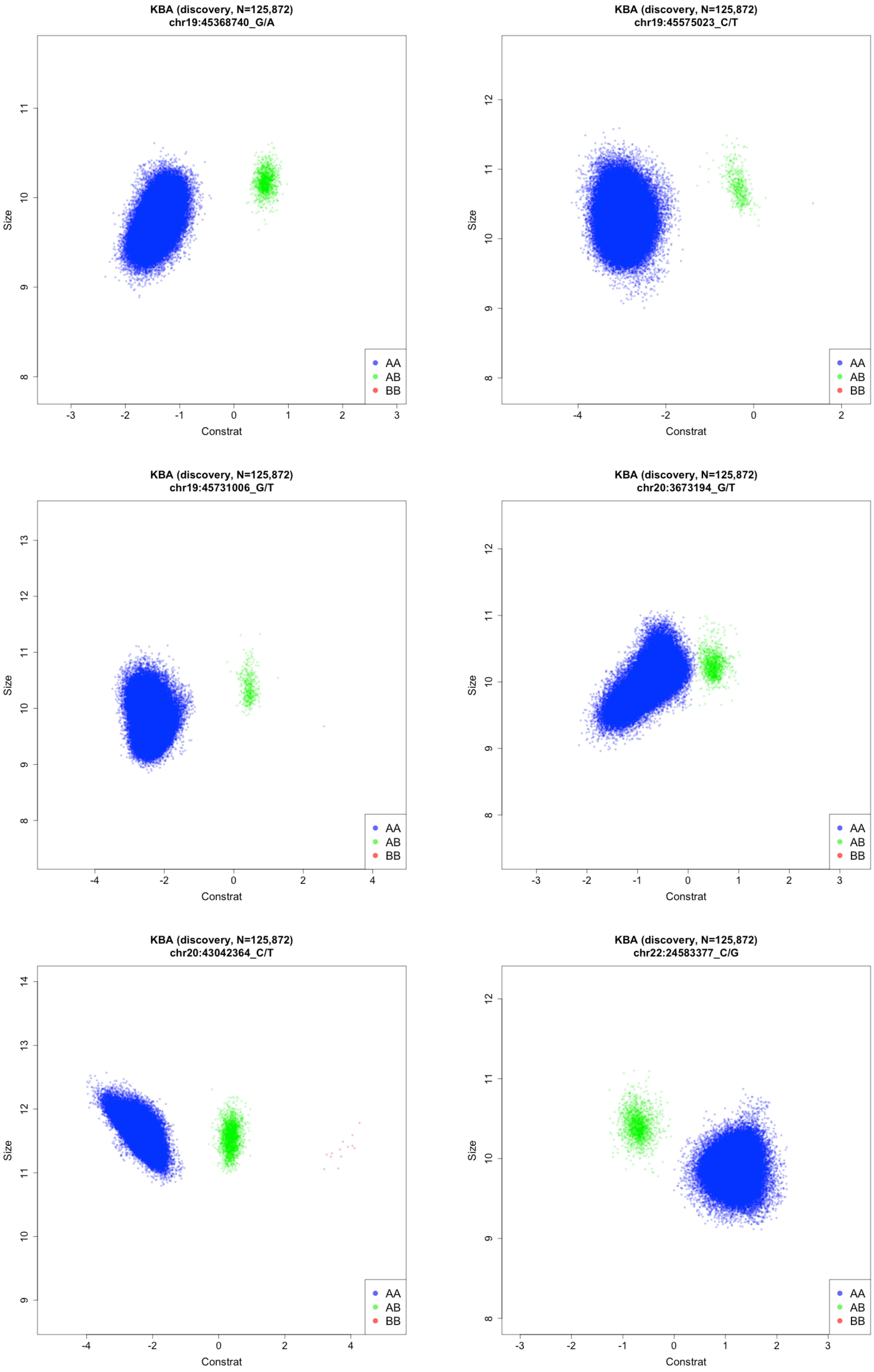

Supplementary Figure 14. Cluster plots of rare variants (Replication study, KBA)

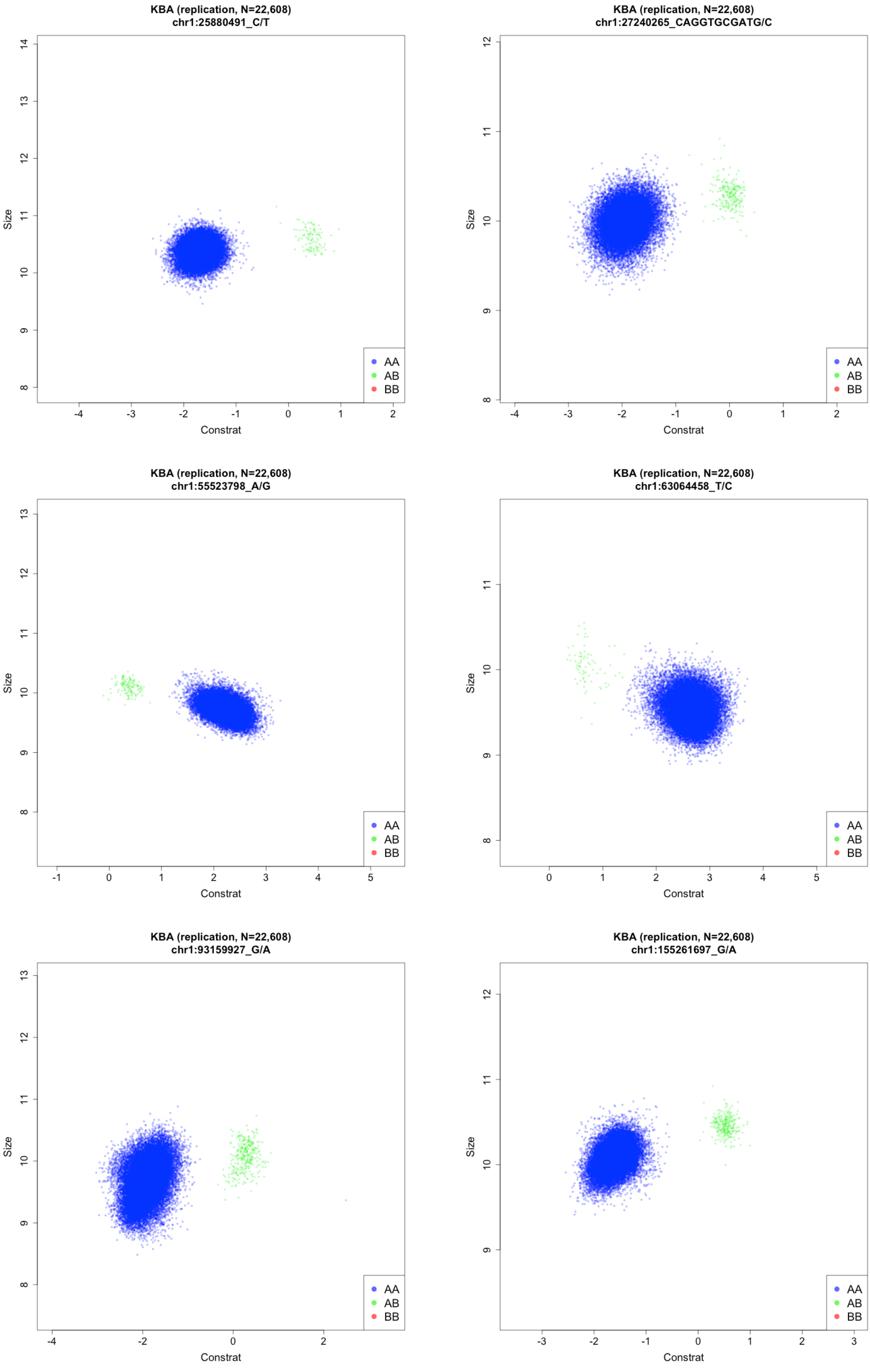

Supplementary Figure 14. Cluster plots of rare variants (Replication study, KBA)

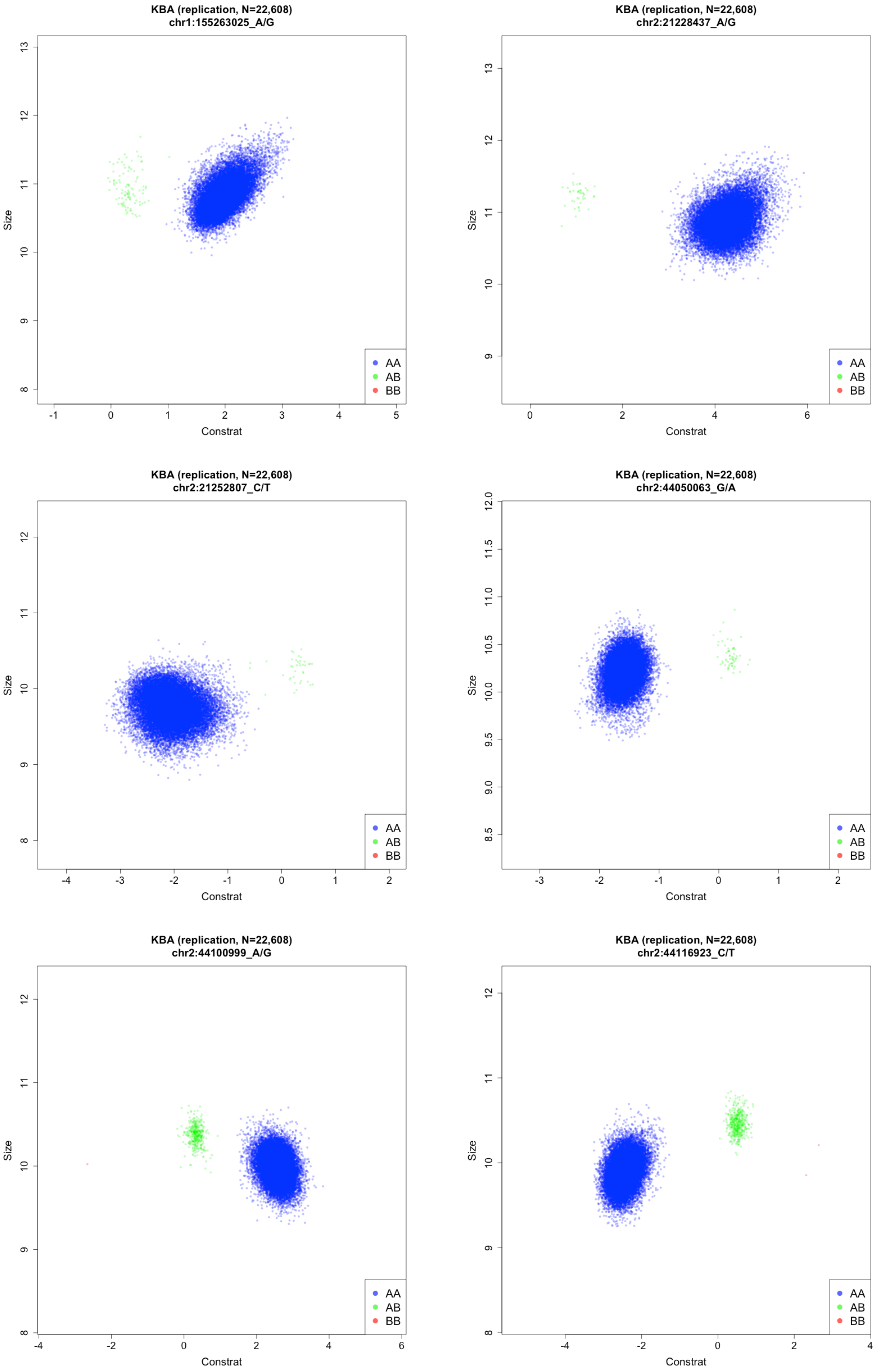

Supplementary Figure 14. Cluster plots of rare variants (Replication study, KBA)

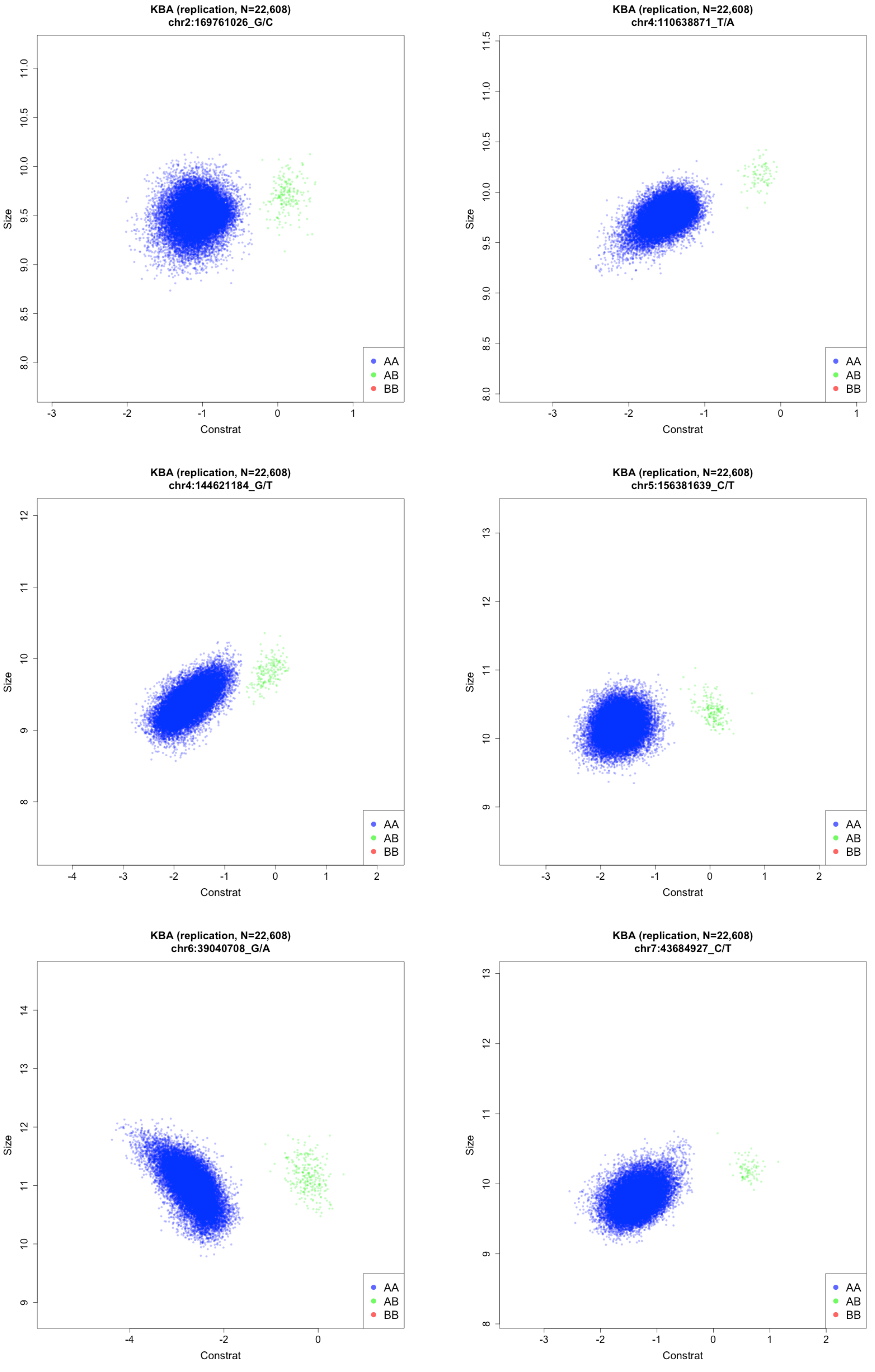

Supplementary Figure 14. Cluster plots of rare variants (Replication study, KBA)

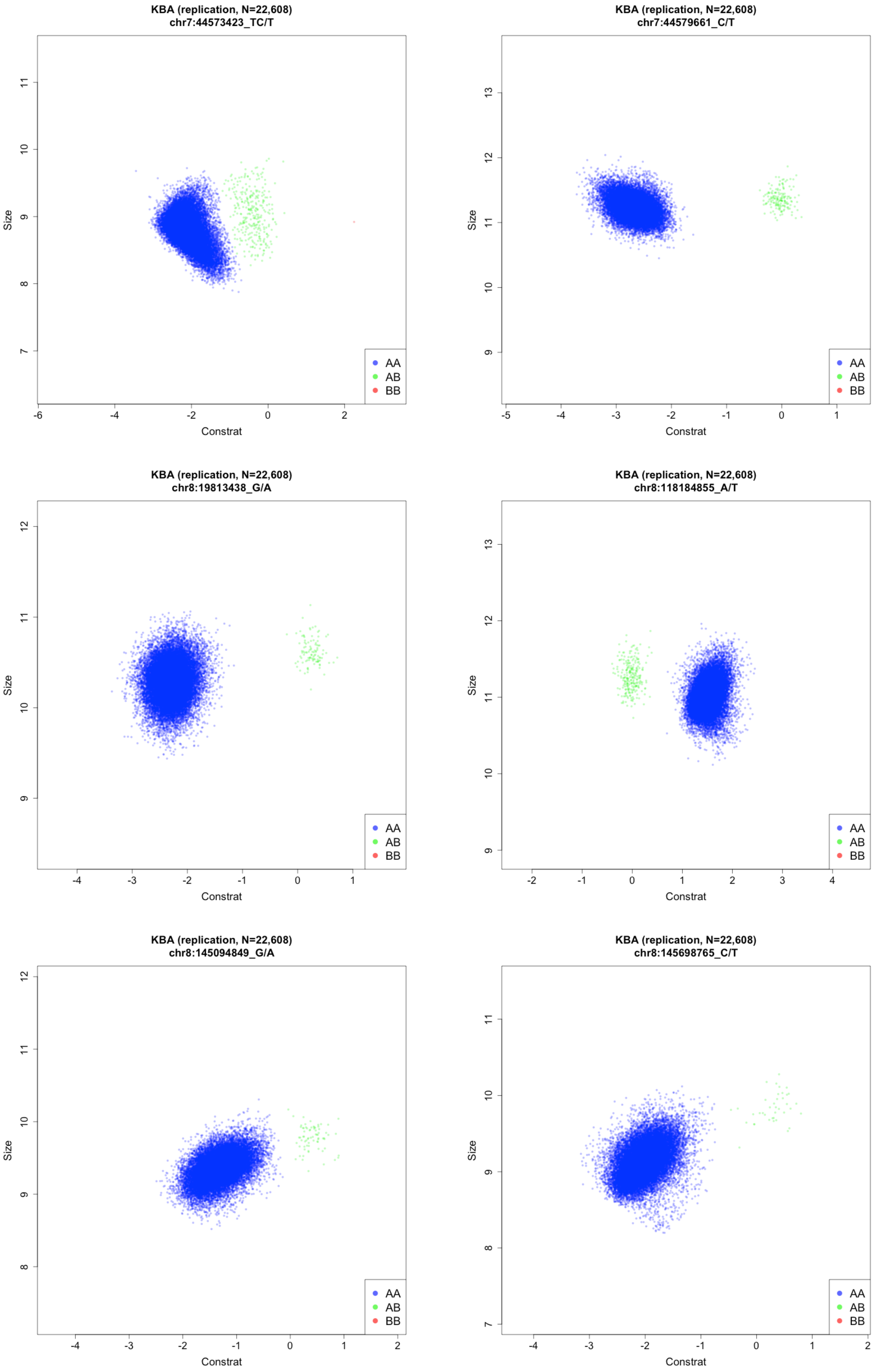

Supplementary Figure 14. Cluster plots of rare variants (Replication study, KBA)

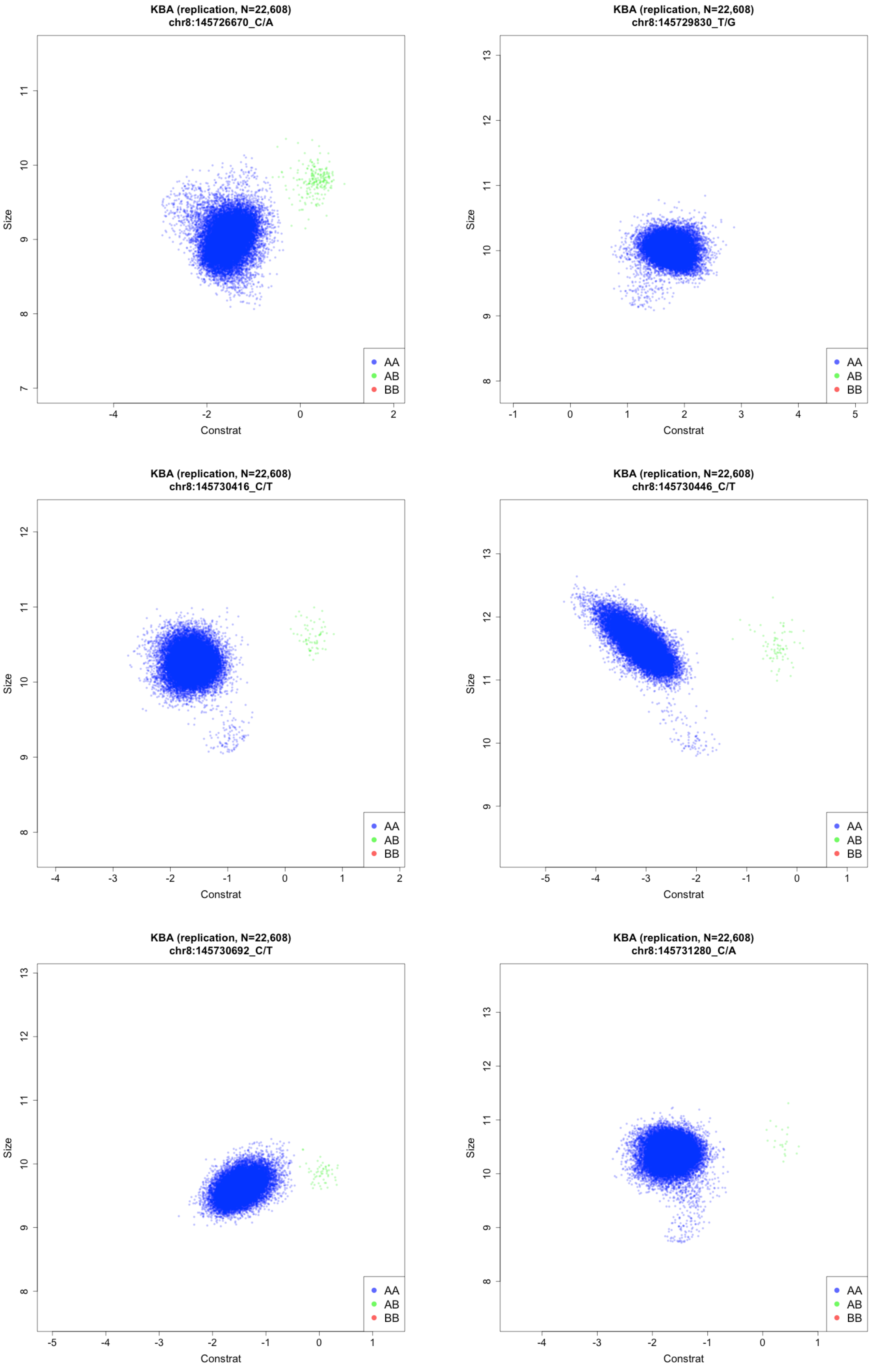

Supplementary Figure 14. Cluster plots of rare variants (Replication study, KBA)

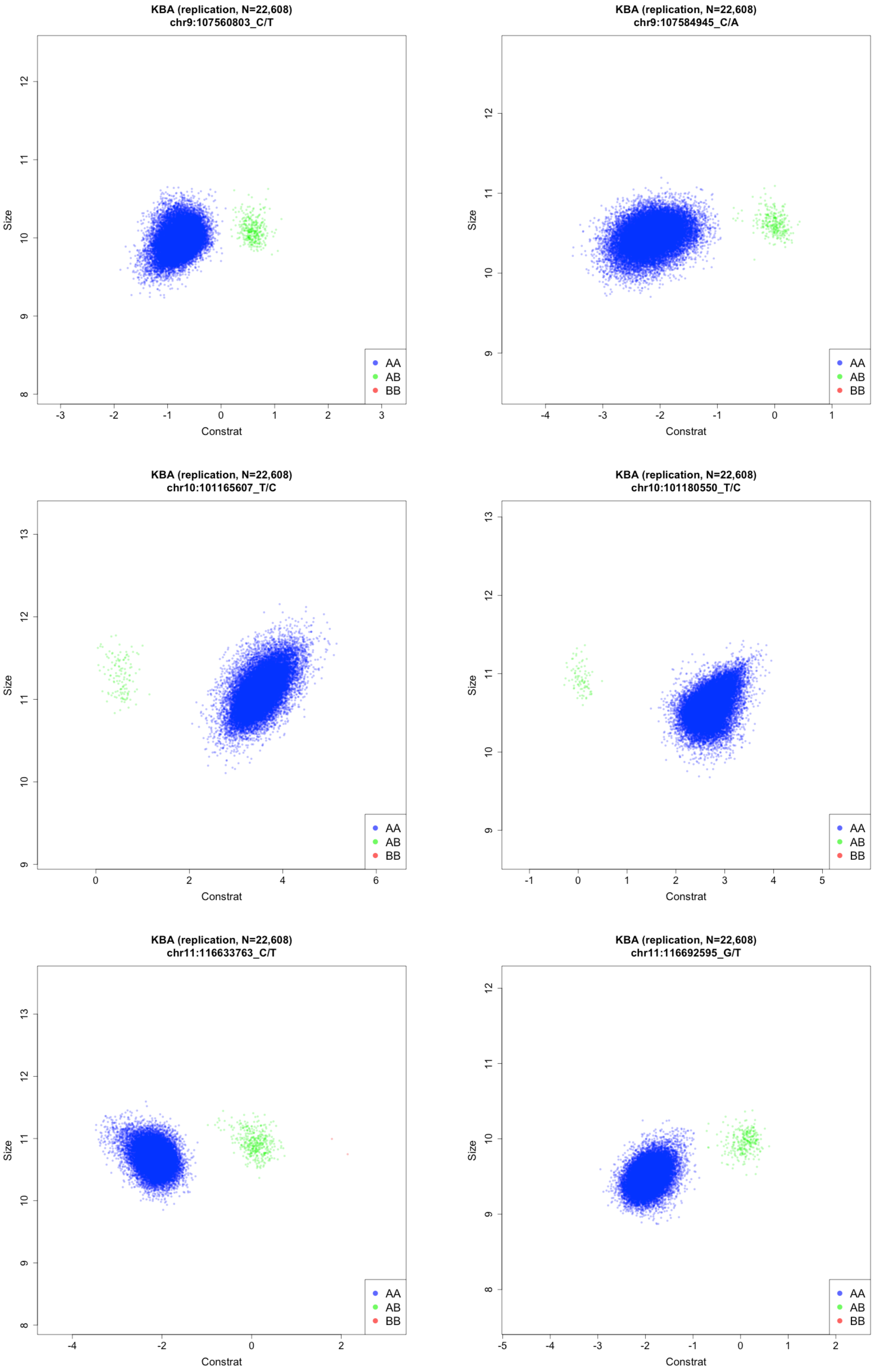

Supplementary Figure 14. Cluster plots of rare variants (Replication study, KBA)

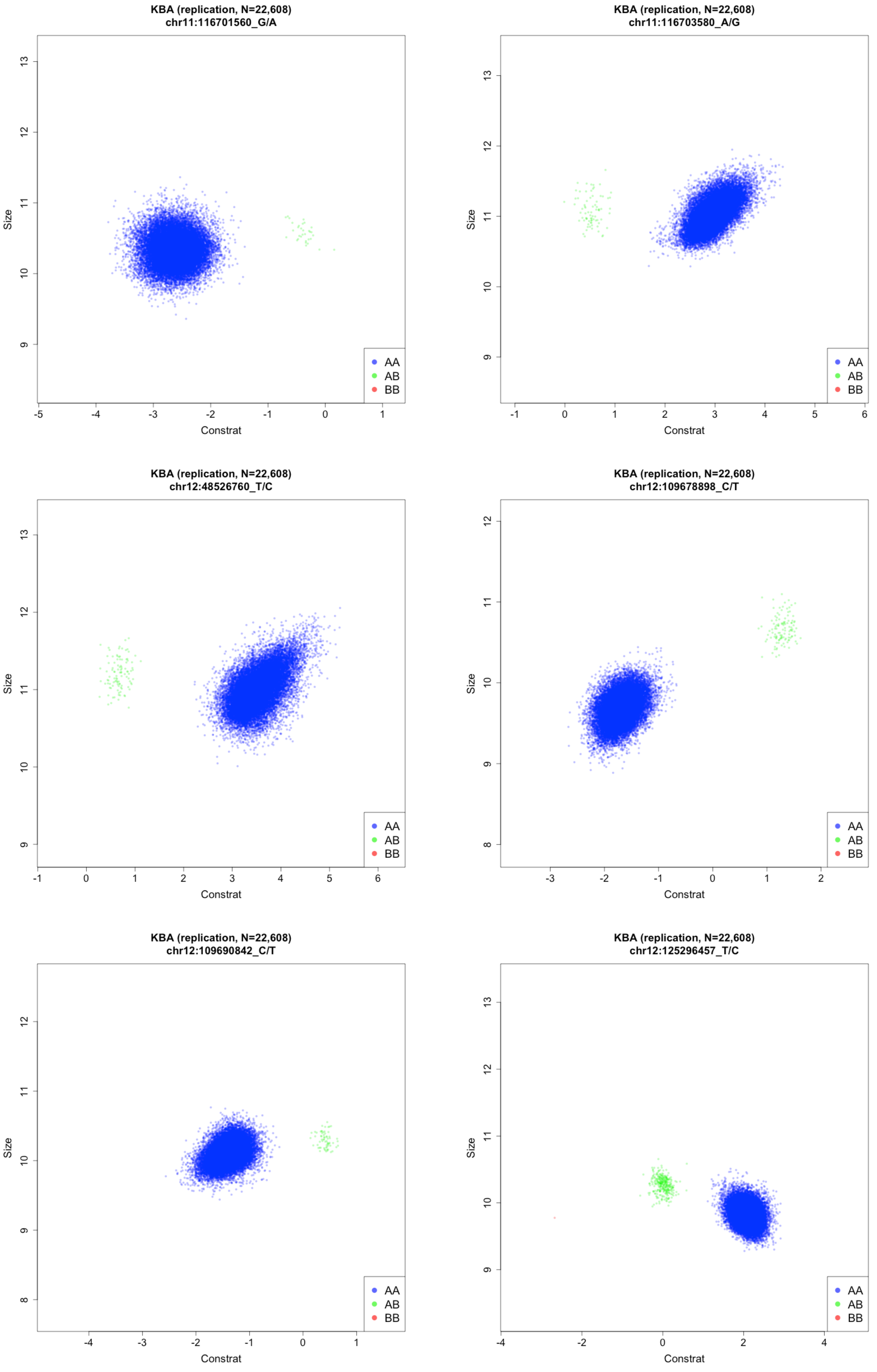

Supplementary Figure 14. Cluster plots of rare variants (Replication study, KBA)

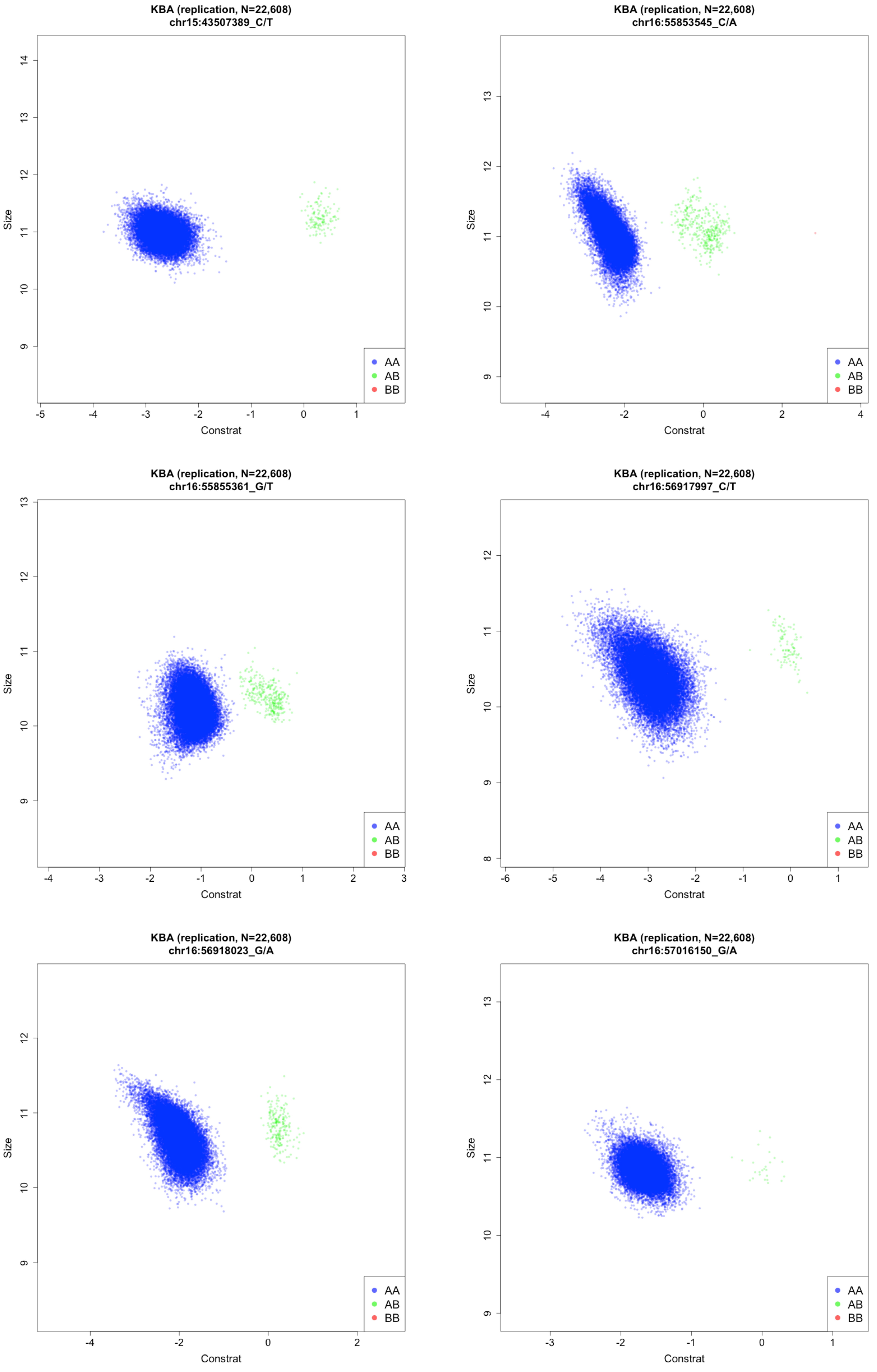

Supplementary Figure 14. Cluster plots of rare variants (Replication study, KBA)

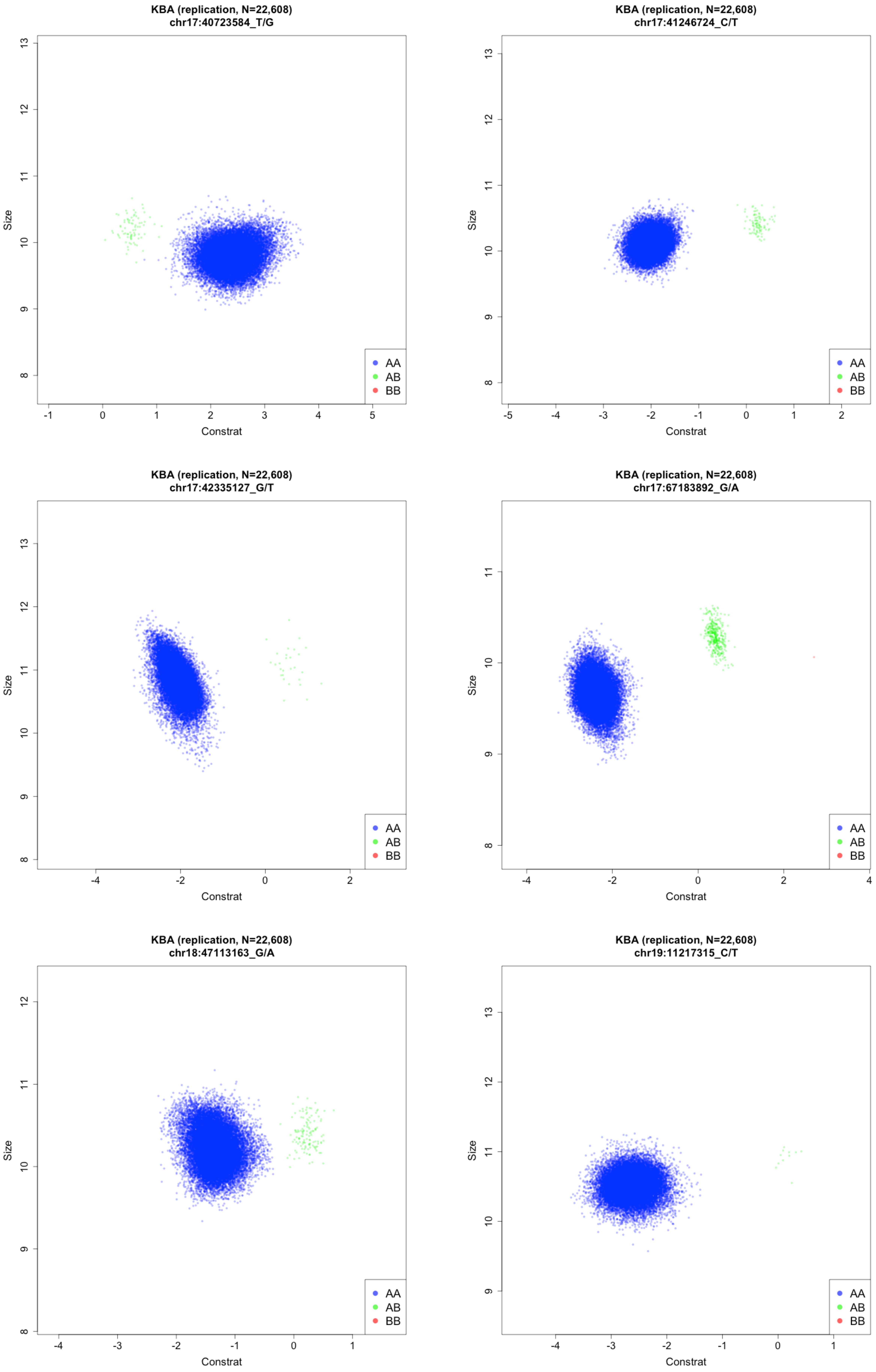

Supplementary Figure 14. Cluster plots of rare variants (Replication study, KBA)

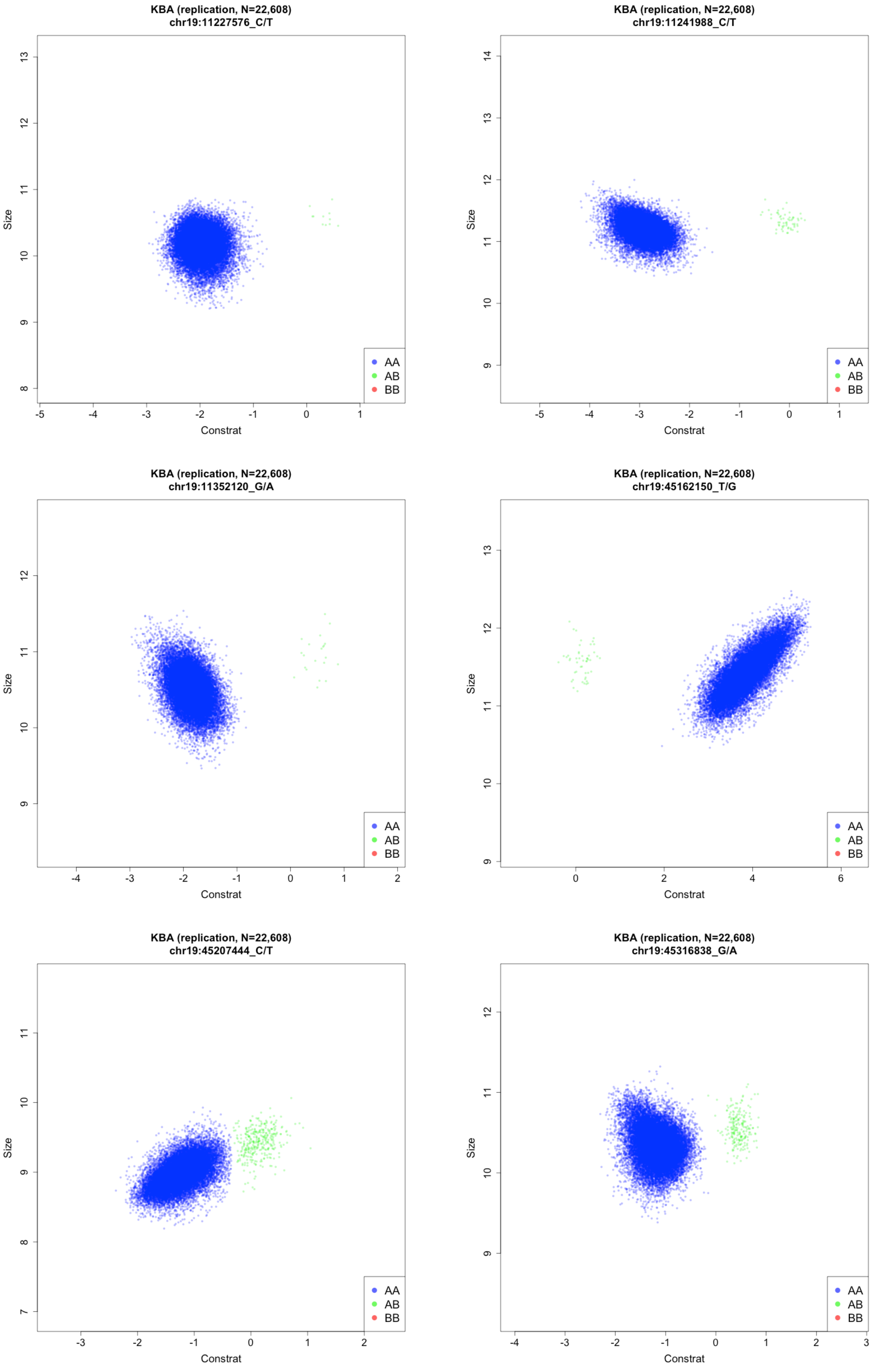

Supplementary Figure 14. Cluster plots of rare variants (Replication study, KBA)

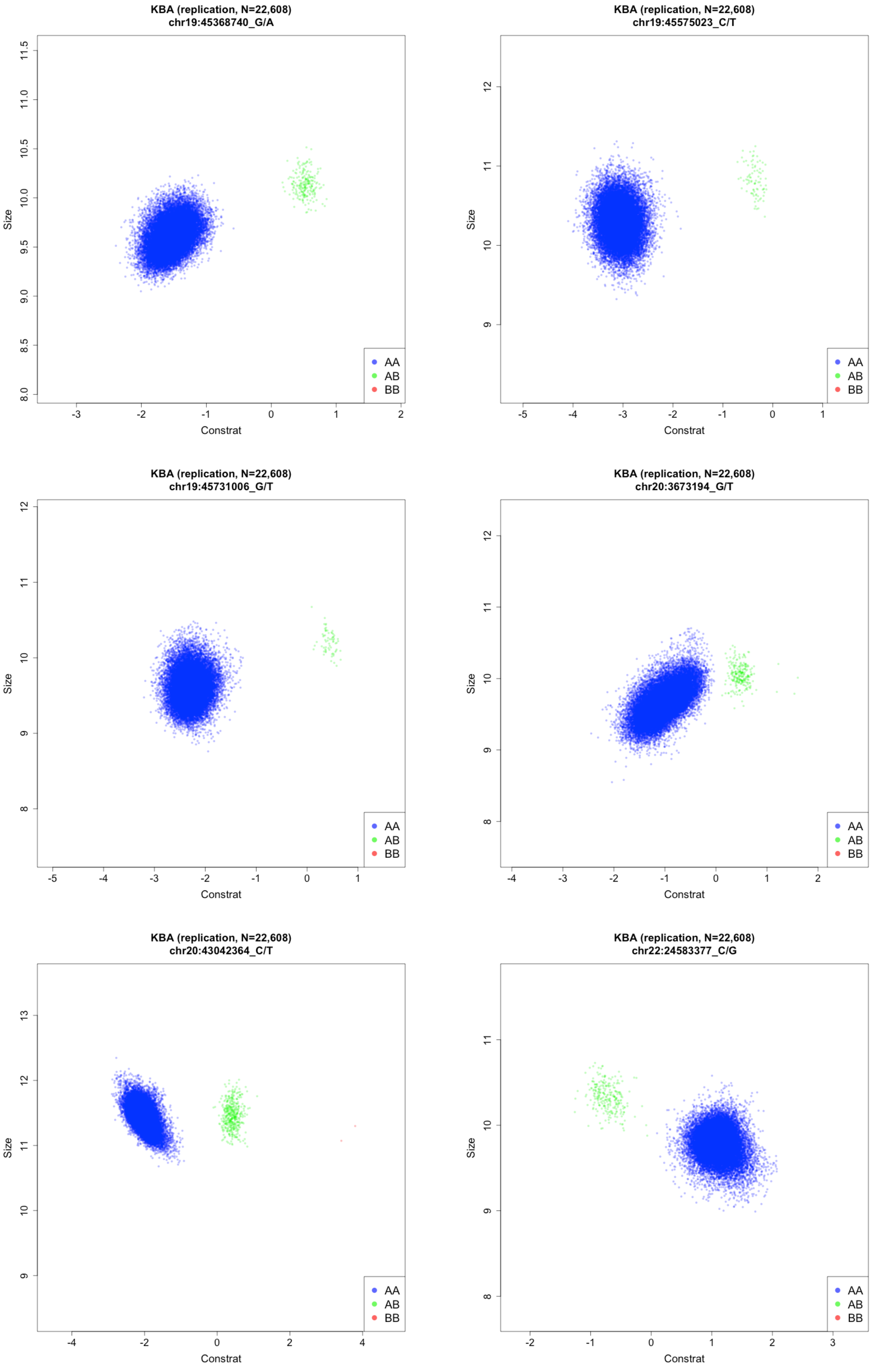

Supplement: Supplementary file 1 — Supplementary Information [file 41467_2022_34163_MOESM1_ESM.pdf]
