# Supplementary material for: The contribution of common and rare genetic variants to variation in metabolic traits in 288,137 East Asians
Source: Nat Commun. 2022 Nov 4;13:6642. doi: 10.1038/s41467-022-34163-2 (PMC9636136; doi:10.1038/s41467-022-34163-2)
Supplement: Supplementary file 2 — Description of Additional Supplementary Files [file 41467_2022_34163_MOESM2_ESM.pdf]

Description of Additional Supplementary Files

File Name: Supplementary Data 1
Description: Demographic characteristics of study samples.
a For meta-analysis, each trait was transformed to follow a normal distriubtion as performed in the Biobank of Japan GWAS study (Kanai et al. Nature Genetics 2018)
b Exclusion criteria were selected as likely influencing biochemical traits (Kim et al. Nature Genetics 2011)

File Name: Supplementary Data 2
Description: Common variants associated with a given metabolic trait in the discovery stage (KBA). Chromosomal positions are based on hg19. Effect size and Effect allele frequency(Eff.freq) are based on effect allele. 'Lost in Stage 2' is marked as 'Lost' if the signal was not significant (P < 5.56e-9) in the 2nd stage of meta-analysis comprising KBA and BBJ GWA results. Locus Start: the first genomic position of the variants (P < 5.56e-9) within the locus, Locus End: the last genomic position of the variants (P < 5.56e-9) within the locus, Locus End: Locus End - Locus Start

File Name: Supplementary Data 3
Description: Genetic correlations of KBA and BBJ. Genetic correlations between KBA and BBJ were estimated using GNOVA software.

File Name: Supplementary Data 4
Description: Common variants associated with a given metabolic trait in meta-analysis combining KBA and BBJ studies. All lead signals were summarized from linear regression analysis results (P <= 5.56e-9, with directional consistency in effect sizes). Chromosomal positions are based on hg19. Effect size and Effect allele frequency(Eff.freq) are based on effect allele. Eff.Freq(Gnomad) indicates a frequency from East Asians of Gnomad database. 'Direction' shows the direction of effect sizes in order of KBA(KV1.0), KBA(KV1.1), and BBJ. HetPval is for heterogeneity p-value from metal software. Locus Start: the first genomic position of the variants (P < 5.56e-9) within the locus, Locus End: the last genomic position of the variants (P < 5.56e-9) within the locus, Locus End: Locus End - Locus Start. LD_r2 shows linkage disequilibrium r2 based on Europeans (1KG P3). P-value(corrected) indicates corrected P based on genomic inflation factors (Supplementary Data 5)

File Name: Supplementary Data 5
Description: Conditional analysis results of common variants. GCTA COJO(conditional and joint regression model) analysis results. Chromosomal positions are based on hg19. Effect size and Effect allele frequency(Eff.freq) are based on effect allele. Eff.Freq(gnomAD) indicates a frequency from East Asians of gnomAD database. Top: the lead signal of the locus, Condi: additional independent signals remained after conditional analysis

File Name: Supplementary Data 6
Description: Summary of association results. Numbers in the table indicate the number of genome-wide significant variants (P <= 5.56e-9 for common variants, P<= 8.12e-8 for rare variants) for a given trait. Number of 'Conditional' is based on GCTA-COJO results(Suppl Data

5). 'Independent' rare variants were counted if a rare variant was independent associated based on conditional analysis results (Supplementary Data 9-12)

File Name: Supplementary Data 7
Description: Association results of rare variants for a given metabolic trait. Chromosomal positions are based on hg19. Effect allele is alternative allele and other allele is reference allele. Effect size and Effect allele frequency(Eff.freq) are based on effect allele. Eff.Freq(gnomAD) indicates a frequency from East Asians of gnomAD database.

File Name: Supplementary Data 8
Description: Previously reported low-frequency or rare variants associated with biochemical traits. Association results were retrieved from previous publications. Only low-frequency (MAF 1-5%) or rare (MAF < 1%) variants were selected. Chromosomal positions were based on hg19.

File Name: Supplementary Data 9
Description: Conditional analysis results for common and rare variants. There were 46 co-incident loci (CL) defined as a cluster of rare variants and common variants from the lead signals in this study or previously reported variants (with P ≤ 1e-4 in this study) within 1Mb apart each other. 8 rare variants were not included in the CLs (1:155263025, 4:144621184, 12:48526760, 15:43507389(for HbA1c), 1:27240265, 20:43042364 (for HDL), 1:155261697, 4:110638871 (for TG)). All analyses were performed using R statistics software. Chromosomal positions are based on hg19. Effect allele is minor allele of the variant. Effect size and Effect allele frequency(Eff.freq) is based on effect allele. Eff.Freq(gnomAD) indicates a frequency from East Asians of gnomAD database. Bold faced associations showed more than 30% decrease in effect size compared to its initial association results after conditional analysis.
1Previously associated variant in GWAS catalogue.

File Name: Supplementary Data 10
Description:. Variants diminished after conditional analysis with other variants resided in the same locus. Chromosomal positions are based hg19. Effect size and Effect allele frequency(Eff.freq) is based on effect allele. Eff.Freq indicates a frequency from popolations in the parenthesis (East Asian and European from Gnomad). The predicted function of a variant was annotated as 'damaging' if two or more prediction softwares interpreted as 'damaging' including LRT, MetaSVM, MutationAssessor, MutationTaster, PROVEAN, Polyphen, and SIFT, retrieved from dbNSFP v2.9 database, 'benign' otherwise.

File Name: Supplementary Data 11
Description: Haplotype analysis results for common and rare variants. Results were summarized from linear regression analysis. Effect was calculated by additive effect of each haplotype based on HAP0 as a reference. Alleles are colored as 'Green', 'Red', and 'Black' for 'Minor allele of common variant', 'Minor allele of rare variant', and 'Major alleles'.

File Name: Supplementary Data 12
Description: Haplotype analysis results using independently associated variants. Results were summarized from linear regression analysis. Effect was calculated by additive effect of each

haplotype based on HAP0 as a reference. Alleles are colored as 'Green', 'Red', and 'Black' for 'Minor allele of common variant', 'Minor allele of rare variant', and 'Major alleles'.

File Name: Supplementary Data 13
Description: Variants used for CV-GRS. Effect size was changed to be positivie value and risk allele was changed accordingly.

File Name: Supplementary Data 14
Description: GRS association with traits using common variants discovered in this study (CV-GRS and ALL-GRS). # of markers indicates the number of common variants and the number of rare variants (in the parenthesis) used for GRS construction. Linear regression analysis was performed to test an association between GRS and trait.

File Name: Supplementary Data 15
Description: Increased level of traits for top high GRS group (CV-GRS). CV-GRS (GRS with common variants) was used for GRS grouping.

File Name: Supplementary Data 16
Description: Variants used for ALL-GRS. Effect size was changed to be positivie value and risk allele was changed accordingly.

File Name: Supplementary Data 17
Description: Interplay of common and rare variants for inherited risk of metabolic traits. Trait quantile: quantile range was assigned if a mean value of the designated group is simmilar to those of a mean value of trait quantiles. Reference group: non-carriers of rare alleles. Risk decreasing: individuals carrying only one or more rare variants decreasing risks in health problem by decreasing levels of metabolic traits or increasing in case of HDL, Risk increaing: individuals carrying only one or more rare variants increasing risks in health problem by increasing levels of metabolic traits or decreasing in case of HDL.

File Name: Supplementary Data 18
Description: CV-GRS association with traits and T2D using all loci discovered in this study
# of markers indicates the number of common variants used for CV-GRS construction. # of samples were used for T2D association is 106,771. Logistic regression analysis was performed to test an association between CV-GRS and T2D.

File Name: Supplementary Data 19
Description: GRSs using Glycemic or erythrocytic variants in association to T2D(CV-GRS)
# of markers indicates the number of common variants used for GRS construction. Logistic regression analysis was performed to test an association between GRS and T2D.

File Name: Supplementary Data 20
Description: FPG associated rare variant tested for an association with T2D. Logistic regression analysis was performed to test an association between rare variants and T2D adjusting for age and sex.

File Name: Supplementary Data 21
Description: Previously reported T2D variants used for T2D-GRS construction. Variants were retrieved from Spracklen et al. Nature 2020. Here variants were listed if available in our dataset. Using T2D cases (N=12,135) and controls(N=94,636) among 126K KBA dataset, Beta and SE were estimated by logistic regression analysis adjusting age and sex.

File Name: Supplementary Data 22
Description: Risk of T2D by T2D-GRS and glycemic traits dervied GRSs. For T2D-GRS construction, previously reported variants by Europeans (Mahajan et al. Nature Genetics 2018) and East Asians (Suzuki et al. Nature Genetics 2019) were used (Supplementary Data 16). A logistic regression model was used to test an association between T2D and GRS adjusted for age and sex. Top GRS groups were compared to median GRS group (40-60%).

File Name: Supplementary Data 23
Description: Ancestry specific and Trans-ethnic weighted CV-GRS. 'N' indicates number of variants used for GRS construction

File Name: Supplementary Data 24
Description: Prevalence of T2D in PRS groups stratified by the presence of a rare protective allele. For PRS groups, T2D prevalence was calculated for non-carriers and carriers of a rare protective allele. For each PRS group, a logistic regression model was used to test an association between T2D and a rare protective variant adjusted for age and sex.
