# Supplementary material for: The contribution of common and rare genetic variants to variation in metabolic traits in 288,137 East Asians
Source: Nat Commun. 2022 Nov 4;13:6642. doi: 10.1038/s41467-022-34163-2 (PMC9636136; doi:10.1038/s41467-022-34163-2)
Supplement: Supplementary file 4 — Reporting Summary [file 41467_2022_34163_MOESM4_ESM.pdf]

nature portfolio

Corresponding author(s): Bong-Jo Kim

Last updated by author(s): 10/06/2022

# Reporting Summary

Nature Portfolio wishes to improve the reproducibility of the work that we publish. This form provides structure for consistency and transparency in reporting. For further information on Nature Portfolio policies, see our Editorial Policies and the Editorial Policy Checklist.

## Statistics

For all statistical analyses, confirm that the following items are present in the figure legend, table legend, main text, or Methods section.

| n/a | Confirmed | |
|---|---|---|
| ☐ | ☒ | The exact sample size (*n*) for each experimental group/condition, given as a discrete number and unit of measurement |
| ☒ | ☐ | A statement on whether measurements were taken from distinct samples or whether the same sample was measured repeatedly |
| ☐ | ☒ | The statistical test(s) used AND whether they are one- or two-sided<br>*Only common tests should be described solely by name; describe more complex techniques in the Methods section.* |
| ☐ | ☒ | A description of all covariates tested |
| ☐ | ☒ | A description of any assumptions or corrections, such as tests of normality and adjustment for multiple comparisons |
| ☐ | ☒ | A full description of the statistical parameters including central tendency (e.g. means) or other basic estimates (e.g. regression coefficient) AND variation (e.g. standard deviation) or associated estimates of uncertainty (e.g. confidence intervals) |
| ☐ | ☒ | For null hypothesis testing, the test statistic (e.g. *F*, *t*, *r*) with confidence intervals, effect sizes, degrees of freedom and *P* value noted<br>*Give P values as exact values whenever suitable.* |
| ☒ | ☐ | For Bayesian analysis, information on the choice of priors and Markov chain Monte Carlo settings |
| ☒ | ☐ | For hierarchical and complex designs, identification of the appropriate level for tests and full reporting of outcomes |
| ☐ | ☒ | Estimates of effect sizes (e.g. Cohen's *d*, Pearson's *r*), indicating how they were calculated |

*Our web collection on statistics for biologists contains articles on many of the points above.*

## Software and code

Policy information about availability of computer code

Data collection

No software was used for data collection

Data analysis

Details are described in the Method section. Software and code include:
Quality control: Plink v1.9, https://www.cog-genomics.org/plink/
Quality control: Qctool v2, https://www.well.ox.ac.uk/~gav/qctool_v2/
Phasing: Eagle v2.3, https://alkesgroup.broadinstitute.org/Eagle/
Imputation: Impute v4, https://jmarchini.org/software/#impute-4
Data format conversion: GEN2VCF v1, https://bitbucket.org/4shin/division-of-genome-research/src/master
Association: EPACTS v2.3, http://genome.sph.umich.edu/wiki/EPACTS
Association: SNPTEST v2.5.2, https://mathgen.stats.ox.ac.uk/genetics_software/snptest/old/snptest.html
Meta-analysis: Metal (March 2011), http://csg.sph.umich.edu/abecasis/Metal/
Conditional analysis: GCTA v1.93, https://cnsgenomics.com/software/gcta
Visualization: R statistics v3.4.4, https://www.r-project.org
Genetic risk score analysis: plink v1.9, https://www.cog-genomics.org/plink/
Functional annotation: FUMA-GWAS, https://fuma.ctglab.nl
Genetic correlation: GNOVA, https://github.com/xtonyjiang/GNOVA
LD score regression: LD SCore v1.01, https://github.com/bulik/ldsc
Annotation: SnpEff, http://pcingola.github.io/SnpEff/
Annotation: SnpSift, http://pcingola.github.io/SnpEff/ss_introduction/
Annotation: dbNSFP v2.9, https://sites.google.com/site/jpopgen/dbNSFP

For manuscripts utilizing custom algorithms or software that are central to the research but not yet described in published literature, software must be made available to editors and reviewers. We strongly encourage code deposition in a community repository (e.g. GitHub). See the Nature Portfolio guidelines for submitting code & software for further information.

# Data

Policy information about availability of data

All manuscripts must include a data availability statement. This statement should provide the following information, where applicable:

- Accession codes, unique identifiers, or web links for publicly available datasets
- A description of any restrictions on data availability
- For clinical datasets or third party data, please ensure that the statement adheres to our policy

Overall meta-analyses summary level results are available through the Korea Biobank Array website (http://koreanchip.org/kba130k/).
The dbNSFP v2.9 file is available through the dbNSFP website (https://sites.google.com/site/jpopgen/dbNSFP)

# Field-specific reporting

Please select the one below that is the best fit for your research. If you are not sure, read the appropriate sections before making your selection.

☒ Life sciences ☐ Behavioural & social sciences ☐ Ecological, evolutionary & environmental sciences

For a reference copy of the document with all sections, see nature.com/documents/nr-reporting-summary-flat.pdf

# Life sciences study design

All studies must disclose on these points even when the disclosure is negative.

| | |
|---|---|
| Sample size | For discovery study, we used the largest available samples with East Asian ancestry including 125,872 Koreans and 162,255 Japanese (BioBank Japan GWAS summary statistics). Our sample size was sufficiently enough to discover numerous common and rare associations at 700 loci (611 common and 89 rare associations). |
| Data exclusions | Genotypes were called per each batch and quality control (QC) of samples and SNPs was conducted by batches. Quality control was conducted as follows: (1) samples QC: exclusion of gender inconsistency, low call rate (< 97%), excessive heterozygosity, and outliers of principle component analysis results. (2) SNP QC: exclusion of poorly clustered SNPs based on the SNPolisher analysis results, missing rate > 5%, and HWE failure P < 1e-6. Furthermore, by analyzing all the batches together, 2nd-degree relatives were removed to secure unrelated genotype data for further analysis. Then, all QCed batches were combined. For common variants (MAF >= 1%), SNPs were further excluded if the missing rate > 10%, allele frequency difference > 0.2 when compared to 1,000 Genomes Project phase 3 East Asians (n = 504) or Korean Reference Genome (n = 397), MAF < 1%, and HWE failure P < 1e-6. For rare variants (MAF < 1%), the putative poorly genotyped clustered rare variants were further excluded based on the following criteria: 1) all batches with large difference in allele frequency (> 0.5%) iteratively compared to mean allele frequency of all other batches, 2) variants with MAF > 1% or minor allele count (MAC) < 30, 3) variants with HWE P < 1e-6, 4) variants with a missing rate > 30%. |
| Replication | For replication study, about 24,000 samples from Health Examinee (HEXA) cohort were additionally genotyped using Korea Biobank Array. After quality control on samples, 22,608 samples were remained and the variants discovered in this study were assessed for replication analysis. Common variants were imputed if the variant is not directly genotyped. Among the quality controlled and imputed data from UK Biobank, we further removed individuals with non-European ancestry and non-independent samples using Data-Field 22006 and 22020. As a result, 337,475 individuals were remained for further analysis. Prior to association analyses, samples with diseases or taking medications likely influencing metabolic traits were removed for further analysis. For validating rare variants, exome sequencing data of UK Biobank was analyzed (N=138,032). Filtering criteria for each trait are summarized in Supplementary Data 1. |
| Randomization | This study performed association analyses and did not require randomization. |
| Blinding | This study performed association analyses and did not require binding. |

# Reporting for specific materials, systems and methods

We require information from authors about some types of materials, experimental systems and methods used in many studies. Here, indicate whether each material, system or method listed is relevant to your study. If you are not sure if a list item applies to your research, read the appropriate section before selecting a response.

## Materials & experimental systems

| n/a | Involved in the study |
| --- | --- |
| ☒ | ☐ Antibodies |
| ☒ | ☐ Eukaryotic cell lines |
| ☒ | ☐ Palaeontology and archaeology |
| ☒ | ☐ Animals and other organisms |
| ☐ | ☒ Human research participants |
| ☒ | ☐ Clinical data |
| ☒ | ☐ Dual use research of concern |

## Methods

| n/a | Involved in the study |
| --- | --- |
| ☒ | ☐ ChIP-seq |
| ☒ | ☐ Flow cytometry |
| ☒ | ☐ MRI-based neuroimaging |

# Human research participants

Policy information about studies involving human research participants

Population characteristics

In the discovery stage, this study included data 125,872 Korean particpants from Korean Genome and Epidemiology Study(KoGES) and 162,255 Japanese from BioBank Japan (BBJ). Sample characteristics for Koreans is described in Supplementary Data 1. For BBJ samples, we used publicly available summary statistics. Sample characteristics for BBJ samples has been described elsewhere (Kanai et al. Nature Genetics 2018).

Recruitment

The Korean Genome and Epidemiology Study (KoGES) was initiated in 2001 to investigate genetic and environmental factors responsible for complex diseases in Koreans. A detailed description of KoGES has been described previously (Kim et al. Int J Epidemiol. 2017). In the three population-based cohorts, 10,030, 173,357, and 28,338 participants were independently recruited from the KoGES_Ansan and Ansung study, the KoGES_health examinee (HEXA) study and the KoGES_cardiovascular disease association study (CAVAS), respectively. Among them, 125,872 Korean samples in the discovery study were genotyped and passed the quality control criteria. Details of genotyped participants are described in Supplementary Data 1. Prior to association analyses, individuals with ongoing medication or therapy that likely influencing metabolic traits, were excluded from the analysis.

Ethics oversight

This study was approved by the institutional review board of the Korea Disease Control Prevention and Control Agency, Republic of Korea. All participants provided written informed consent.

Note that full information on the approval of the study protocol must also be provided in the manuscript.
